# Experimental determination and data-driven prediction of homotypic transmembrane domain interfaces

**DOI:** 10.1016/j.csbj.2020.09.035

**Published:** 2020-10-07

**Authors:** Yao Xiao, Bo Zeng, Nicola Berner, Dmitrij Frishman, Dieter Langosch, Mark George Teese

**Affiliations:** a Center for Integrated Protein Science Munich (CIPSM) at the Lehrstuhl für Chemie der Biopolymere, Technische Universität München, Weihenstephaner Berg 3, 85354 Freising, Germany; b Department of Bioinformatics, Wissenschaftszentrum, Weihenstephan, Maximus-von-Imhof-Forum 3, Freising 85354, Germany; c Department of Bioinformatics, Peter the Great Saint Petersburg Polytechnic University, St. Petersburg 195251, Russian Federation; d TNG Technology Consulting GmbH, Beta-Straße 13a, 85774 Unterföhring, Germany

**Keywords:** Protein-protein interaction, TMD interactions, Machine learning, Transmembrane, GxxxG, Co-evolution

## Abstract

Interactions between their transmembrane domains (TMDs) frequently support the assembly of single-pass membrane proteins to non-covalent complexes. Yet, the TMD-TMD interactome remains largely uncharted. With a view to predicting homotypic TMD-TMD interfaces from primary structure, we performed a systematic analysis of their physical and evolutionary properties. To this end, we generated a dataset of 50 self-interacting TMDs. This dataset contains interfaces of nine TMDs from bitopic human proteins (Ire1, Armcx6, Tie1, ATP1B1, PTPRO, PTPRU, PTPRG, DDR1, and Siglec7) that were experimentally identified here and combined with literature data. We show that interfacial residues of these homotypic TMD-TMD interfaces tend to be more conserved, coevolved and polar than non-interfacial residues. Further, we suggest for the first time that interface positions are deficient in β-branched residues, and likely to be located deep in the hydrophobic core of the membrane. Overrepresentation of the GxxxG motif at interfaces is strong, but that of (small)xxx(small) motifs is weak. The multiplicity of these features and the individual character of TMD-TMD interfaces, as uncovered here, prompted us to train a machine learning algorithm. The resulting prediction method, THOIPA (www.thoipa.org), excels in the prediction of key interface residues from evolutionary sequence data.

## Highlights

• Homotypic TMD interfaces identified by different techniques share strong similarities.
• The GxxxG motif is the feature most strongly associated with interfaces.
• Other features include conservation, polarity, coevolution, and depth in the membrane
• The role of each of each feature strongly depends on the individual protein.
• Machine-learning helps predict interfaces from evolutionary sequence data

## 1 Introduction

Bitopic (single-pass) proteins make up ~40% of all integral membrane proteins in mammals [1]. Sequence-specific interactions between their transmembrane domains (TMDs) frequently contribute to the formation of homomeric or heteromeric dimers or multimers in cellular membranes, with consequences for the functionalities of these proteins.

Currently, the structures of only ~20 TM homodimers have been solved by NMR spectroscopy and X-ray crystallography [2], [3], [4], and some of these have homologous sequences. Other TMD-TMD interfaces have been characterised in a biological membrane using methods which we collectively term *E. coli* TM reporter assay (ETRA) techniques, such as the ToxR assay [5], TOXCAT [6], the recently developed dsTβL [7] or the GALLEX assay [8]. In combination with scanning mutagenesis, these assays have exhaustively explored several additional TM helix-helix interfaces. There are many reports where limited mutagenesis has provided sparse information on interface residues. Most TMD-TMD interfaces remain unexplored.

To close the gap between the numbers of well characterised TMD-TMD interfaces and the unknown ones, various methods have been devised previously to predict them from primary structure. These approaches rest on the known structural and evolutionary properties of TMD-TMD interfaces. These properties have been primarily derived from polytopic proteins where heterotypic TMD-TMD interactions support folding. Combined structural and bioinformatic approaches have shown that the TMD-TMD interfacial (i.e. buried) residues are generally more conserved and more polar than lipid-facing residues [9], [10], [11], [12]. Further, sequence coevolution, also known as covariance or evolutionary couplings, is an indicator of contacting residues in both soluble and membrane proteins [13], [14], [15], [16]. TMD-TMD interfaces are generally well packed and display a preference for small residues such as Gly, Ala, Ser and Cys [11], [12], [17]. These residues are thought to contribute to helix-helix interaction by supporting Van der Waals interactions and by allowing for inter-helical C_α_ H-bonding [18], [19], [20]. In comparison to the relatively abundant information on polytopic membrane protein folding, the factors stabilising homotypic TMD-TMD interfaces in non-covalent membrane protein assembly are less understood, and rest on a few case studies. These have emphasised the role of simple sequence motifs, including GxxxG and (small)xxx(small) motifs (small = Gly, Ala, Ser and Cys) [5], [21], [22], [23], [24], [25], [26]. The GxxxG motif is also a dominant feature of many artificial TMDs selected for self-affinity [27], [28], [29], [30], [31], [32], [33]. These motifs are overrepresented in TMD sequences [34]. Their overabundance at natural homotypic TMD interfaces is often assumed but has never been proven via statistical analyses. Based on case studies alone, the presence of these motifs is often assumed to indicate self-interaction or the presence of an interface. As a consequence, motifs are usually the first residues to be targeted in mutagenesis experiments [35], [36], [37], [38], [39]. There is a strong need for statistical analyses to objectively define the importance of these motifs and other sequence properties in homotypic TMD interaction. A major impediment is the lack of appropriate data.

There are several automated methods that identify TMD homodimer structures from TMD sequences alone using energy functions: PREDDIMER [40], [41], CATM [42], EFDOCK-TM [43], TMDOCK [44], TMDIM [45], and TMHOP [46]. The PREDDIMER algorithm works by establishing the maximal complementarity of hydrophobic or hydrophilic surfaces of TMD homodimers. This is followed by geometry optimisation and structure refinement. CATM is a specialised method that is only applicable to dimers driven by (small)xxx(small) motifs. The EFDOCK-TM prediction pipeline incorporates evolutionary data based on the output of the LIPS algorithm [47] and also coevolution scores. LIPS was originally designed to predict lipid-facing residues in polytopic proteins and can identify a helix face with high conservation and polarity. EFDOCK-TM then identifies residue pairs via “evolutionary constraints”, as derived from sequence coevolution in the LIPS interface. Random combinations of evolutionary constraints are finally used to guide modelling via Rosetta membrane [48]. The TMDOCK algorithm threads a target amino acid sequence through several structural templates, followed by local energy minimisation. TMHOP utilises an experimentally determined hydrophobicity scale and ROSETTA modelling; it is a purely energy-based predictor and can also predict higher-order oligomers.

As yet, none of the above predictors incorporate any machine-learning components for contact or interface recognition from evolutionary data. Machine learning predictors are available for related problems including the prediction of contacting residues within folded polytopic proteins [16], [49], and the prediction of homodimer interface residues of membrane proteins based on a submitted protein structure [49], [50], [51]. Unfortunately, the latter algorithms are not applicable to self-interacting TMDs of bitopic proteins, for which structures are rarely available.

There is a strong need for algorithms that help identify putative homotypic TM interface residues, in order to guide experimental approaches. The current generation of energy-based predictors is poorly suited to this task, due to several key challenges. Firstly, there are only a few well-characterised homotypic TMD-TMD dimer structures by which the above algorithms have been validated. Secondly, rather than reproducing residue-residue contacts, validation has been conducted using the C_α_ root mean square deviation (RMSD) for all [40], [41], [42], [44] or subsets [43] of TMD residues. In other cases, interface prediction has been validated using a “percentage of native contacts” method [43] that may be biased by factors such as the length of TMD or percentage of interface residues, and does not specify the improvement above a random selection. While the validation of protein–protein interaction (PPI) site predictions for soluble proteins has been standardised in the Critical Assessment of PRediction of Interactions (CAPRI) initiative [52], [53], there are no such guidelines for membrane proteins, nor have comparative assessments of predictive success been conducted. Thirdly, each of the above prediction algorithms generates an ensemble of possible dimer structures, which the user must interpret subjectively. As we have commented previously [36], wetlab researchers typically identify potential TMD interface residues in a subjective manner based on simple sequence-motifs (e.g. GxxxG) and/or sequence conservation, rather than the output of energy-based prediction algorithms. Taken together, the challenges in the automated prediction of homotypic TMD interfaces remain daunting, even without considering the complex effects of cellular location, membrane properties, membrane inhomogeneity, attached soluble domains, or the interfering presence of other proteins.

Here, we generated and characterised a comprehensive dataset of 50 homotypic TMD interfaces. We show that interface residues tend to exhibit higher conservation, polarity, coevolution and depth in the bilayer, and a lower proportion of β-branched residues. We also affirm the predictive power of the known helix-helix interaction motif, GxxxG. We then created Transmembrane HOmodimer Interface Prediction Algorithm (THOIPA), a machine-learning-based method that compares favourably in its ability to predict TMD homodimer interfaces from primary structure.

## 2 Results

**Fig. 1:**
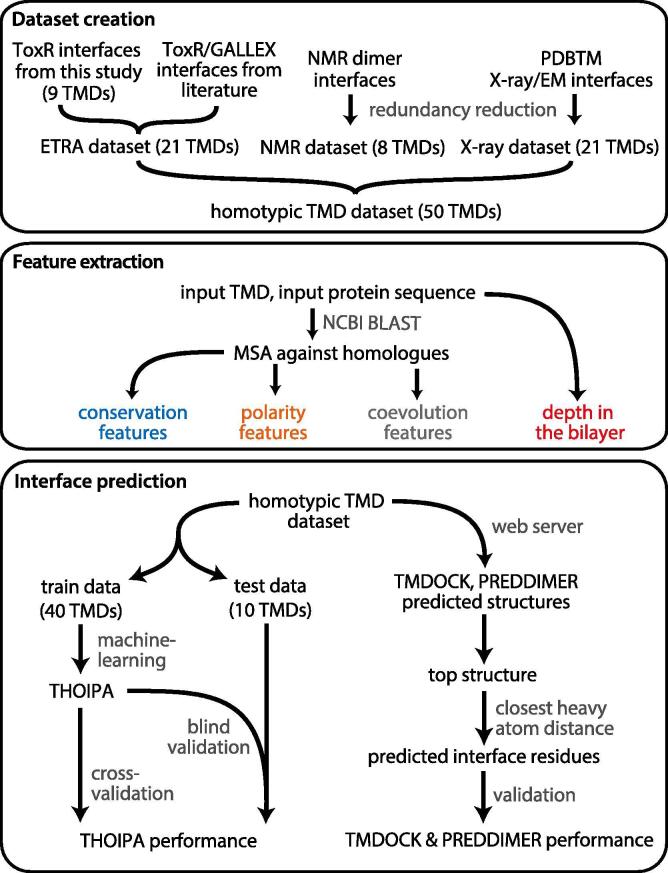
Overview of dataset creation, feature extraction, and interface prediction. Dataset creation: The interface residues of 9 self-interacting TMDs were obtained by experimental analysis in this study using ToxR, an *E. coli* TM reporter assay (ETRA) technique. Other homotypic TM interfaces investigated by ETRA, NMR, and structural techniques were derived from literature or structure databases. Data from these sources were normalised and combined to form a single dataset derived from 50 non-homologous, self-interacting TMDs. Feature extraction: For each interface or non-interface residue in each of the 50 TMDs, we extracted features (properties) based on conservation, polarity, co-evolution, and depth in the bilayer. To determine which of these features are associated with homotypic TMD interaction, we compared their values between interface and non-interface residues for all residues in the homotypic TMD dataset. The features comprise the input for a machine-learning algorithm to predict homotypic TM interface residues. Interface prediction: The dataset was split into train data and test data. The train data was used for machine learning, yielding THOIPA. Interface prediction was validated for THOIPA, as well as the automated structural predictors TMDOCK and PREDDIMER. For TMDOCK and PREDDIMER, we extracted the predicted interface residues from the top-ranked 3D dimer structure.

The aims of this study are laid out in Fig. 1. First, we assembled a set of 50 well-characterised interfaces from a broad range of self-interacting TM helices (Table 1). The full homotypic TMD dataset comprises 21 TMDs investigated by ETRA techniques, 8 TMDs investigated by NMR and 21 TMDs from structure databases that were mostly investigated by X-ray crystallography. Second, a quantitative analysis of interface residue properties was conducted. Third, we developed THOIPA and compared its performance to TMDOCK and PREDDIMER.

**Table 1:** Interface residues of the homotypic TMD dataset.

#	Protein (acc^a^) [ref]	TMD sequence^b^
**ETRA TMDs**		
**1**	**Ire1 (O75460)^f^**	**ATIILSTFLLIGWVAFIITY**
**2**	**ATP1B1 (P05026)**[38]**^f^**	**LLFYVIFYGCLAGIFIGTIQVMLLTI**
**3**	**PTPRG (P23470)**[39]	**IIPLIVVSALTFVCLILLIAVLV**
**4**	**Tie1 (P35590)**[37]	**LILAVVGSVSATCLTILAALLTLV**
**5**	**DDR1 (Q08345)**[37]	**ILIGCLVAIILLLLLIIALML**
**6**	**PTPRO (Q16827)**[39]	**VVVISVLAILSTLLIGLLLVTLIIL**
**7**	**Armcx6 (Q7L4S7)**[35]	**REVGWMAAGLMIGAGACYCV**
**8**	**PTPRU (Q92729)**[39]	**LILGICAGGLAVLILLLGAIIVII**
**9**	**Siglec7 (Q9Y286)**[35]	**VLLGAVGGAGATALVFLSFC**
10	GpA (P02724) [7]	LIIFGVMAGVIGTIL
11	ErbB2 (P04626) [7], [56]	LTSIISAVVGILLVVVLGVVFGIL
12	ITGB3 (P05106) [57]	VLLSVMGAILLIGLAALLI
13	ITGA2B (P08514) [58]^f^	WVLVGVLGGLLLLTILVLAMW
14	FtsB (P0A6S5) [59]	TLLLLAILVWLQYSLWF
15	GP1BB (P13224) [60]	GALAAQLALLGLGLLHALLL
16	MPZ (P25189) [61]	YGVVLGAVIGGVLGVVLLLLLLFYVV
17	PTPRJ (Q12913) [39]	ICGAVFGCIFGALVIVTVGG
18	BNIP3 (Q12983) [62]^f^	LLSHLLAIGLGIYIG
19	QSOX2 (Q6ZRP7) [63]	CVVLYVASSLFLMVMY
20	ADCK3 (Q8NI60) [64]	LANFGGLAVGLGFGALA
21	NS4A (Q99IB8) [65]	TWVLAGGVLAAVAAYCLAT

**NMR TMDs**		
22	CD3ζζ (P20963, 2hac)^f^	LCYLLDGILFIYGVILTALFL
23	EphA1 (P21709, 2k1k)	IVAVIFGLLLGAALLLGILVF
24	TYROBP (O43914, 2l34)	LAGIVMGDLVLTVLIALAVYFL
25	APP (P05067, 2loh)	AIIGLMVGGVVIATVIVITLVML
26	PDGFRB (P09619, 2l6w)	VVVISAILALVVLTIISLIILIMLW
27	FGFR3 (P22607, 2lzl)	VYAGILSYGVGFFLFLLVVAAVTLC
28	TLR3 (O15455, 2mk9)^f^	FFMINTSILLI̲FIFIVLL
29	DR5 (O14763, 6nhw)	SGIIIGVTVAAVVLIVAVFVCKSLL

**X-ray TMDs**		
30	KvAP (P01837, 1orqC4)	GKVIGIAVMLTGISALTLLIGTVSNMFQ
31	Bacteriorhodopsin (Q8YSC4, 1xioA4)	GFLMSTQIVVITSGLIADL
32	PSII-M (Q8DHA7, 2axtM1) ^d^	ATALFVLVPSVFLIILYV
33	Mgst1 (P08011, 2h8aA2)	HLNDLENIVPFLGIGLLYSL
34	Wza (Q9X4B7, 2j58A1)^f^^d^	SQLVPTISGVHDMTETVRYI
35	p2X purinoceptor (Q6NYR1, 3h9vA2)	KFNIIPTLLNIGAGLALLGLVNVICDWIV
36	GluCl α (G5EBR3, 3rifA2)	IPARVTLGVTTLLTMTAQSAGIN
37	KCNJ12 (F1NHE9, 3spcA2)	PLAVFMVVVQSIVGCIIDSFMIGAIMAKM
38	fn ATPase F0 c-ring (Q8RGD7, 3zk1A1)^f^	LGCSAVGAGLAMIAGLGPGIGEG
39	CRCM1 (Q9U6B8, 4hksA1)	SWTSALLSGFAMVAMVE
40	CorA (Q9WZ31, 4i0uA1)	TIIATIFMPLTFIAGIYGMNF
41	pntAB (Q72GR9, 4o9pC1)	WSALYI̲FVL̲T̲AF̲L̲GYE̲L̲
42	AbgT (Q0VR69, 4r0cA7)	ITAMEVTMASMAGYLVLMFF̲AAQFVAWF
43	TspO (Q81BL7, 4ryiA2)^f^	PGMTIGMIWAVLFGLIALSVA
44	TMEM16 (C7Z7K1, 4wisA1)	LKAWGLLLSILFAEHFYLVVQLAVR
45	Trpv1 (O35433, 5irzD6)^e^	KAVFIILLLAYVILTYILLLNMLIALM
46	CRCB TM1 (Q7VYU0, 5nkqA1)^f^	F̲IAI̲G̲IGAT̲LGAW̲LRW̲VLG
47	CRCB TM3 (Q7VYU1, 5nkqA3)	AAVTGFLGGLTTFSTFSAETV
48	PC2 (Q13563, 5t4dA6)^e^	RVLGPIYFTTFVFFMFFILLNMFLAIIN
49	BCNG-1 (O60741, 5u6oA6)^e^	ITMLSMIVGATCYAMFVGHATALI
50	NadC (Q9KNE0, 5uldA9)	WKEIQKTADWGILLLFGGGLCL

^a^Accession number (acc) from the UniProt database. The X-ray identification code (e.g. 1orqC4) consists of the PDB accession (e.g. 1orq), the protein chain (e.g. C), and the TMD number in the protein (e.g. 4).

^b^Homotypic interface residues in the TMD sequences are underlined.

^c^Bold text indicates new interfaces identified in the current study. In these cases, the reference indicates the ETRA study in which the TMD was first tested, rather than the source of the mutagenesis data.

^d^TMDs in the X-ray dataset derived from bitopic proteins.

^e^TMD investigated by high-resolution electron microscopy.

^f^TMDs included in the blind test data for THOIPA validation.

### 2.1 The ETRA dataset of TMDs self-interacting in a membrane

Nine novel non-homologous interfaces were determined experimentally in this study, using scanning mutagenesis in combination with the ToxR assay (proteins shown in bold in Table 1, Fig. S1). The nine TMDs included two receptor tyrosine kinases (DDR1, Tie1), three receptor tyrosine phosphatases (PTPRU, PTPRG, PTPRO), and four other human TMDs of unrelated protein families (Siglec7, Armcx6, ATP1B1, and Ire1). All have a high level of self-affinity. In the ToxR assay, the mean level of affinity was 153% of the well characterised high-affinity Glycophorin A TMD, GpA [5]. To identify interface residues, we tested the effects of 263 mutations at 203 positions (29 mutations per TMD, Fig. S2), mostly to Ala (160 mutations) or Ile (51 mutations).

A detailed assessment of these nine TMD interfaces is available in Text S2. They include three that are dominated by (small)xxx(small) motifs (siglec7, Armcx6), one dependent on a key aromatic residue (Ire1) and two dependent on aliphatic residues (DDR1, PTPRO). For Ire1, our data independently corroborates the key role of W457 as proposed in a recent functional study [54]. The remaining interfaces were more difficult to classify, being composed of a mixture of small and aliphatic residues (PTPRU, PTPRG), or a mixture of small, aromatic, and strongly polar residues (ATP1B1). For DDR1 and ATP1B1 (Na/K-ATPase β‑subunit), some interfacial residues had previously been proposed after limited mutagenesis. We confirm that the DDR1 interface relies on a Leu/Ile-rich “leucine zipper” motif [37], [55]. We determined the interface not only of DDR1, but also its homologue DDR2 [37], to whom it shared 71% identity in the TMD region. Scanning mutagenesis of DDR2 revealed a highly similar interface to DDR1 (Fig. S3). This confirms the evolutionary conservation of TMD interfaces, which in turn emphasises the importance of using non-redundant datasets for statistical and machine-learning analyses. We therefore excluded DDR2 from the overall dataset for analysis. For ATP1B1 we confirm that the interface includes a GxxxG motif, as previously proposed by Barwe et al. [38].

We then combined the experimental data from this study and from the literature (Table 1) to create the complete ETRA dataset that includes 21 TMDs, with data from 862 mutations at 432 positions (Fig. S2). For each mutation, we calculated the disruption to dimerisation [35] as described in the methods. Disruption is positive for mutations that decrease dimerisation, and negative for mutations that increase dimerisation [66]. A cut-off value for disruption was then chosen to define interface and non-interface residues in all TMDs. Since there is no precedent, and the data from different studies was quite heterogeneous, we used a cut-off value (0.24) that yielded 3–10 interface residues for each TMD. At this cut-off, the mean number of interface residues in each TMD was 5.2, and interface residues comprised 21% of the total (Fig. S4A). Summing up the numbers of interface residues at each position for all 21 TMDs shows that they tend to follow a pattern of α-helical periodicity and accumulate at the centre of the TMDs (Fig. S4B).

### 2.2 Creation of the complete homotypic TMD dataset

We combined the ETRA dataset with homotypic interfaces derived from NMR and X-ray structures. The NMR dataset consisted of TMD dimers from literature, after removing redundant sequences as well as three TMDs already investigated by ETRA techniques (GpA, BNIP3, and ErbB2). For GpA and ErbB2 the study of Elazar [7] supplied the interface residues with unprecedented precision, based on over 100 mutations in each TMD, and where the effect of each mutation on dimerisation was tested in a natural membrane environment. Although many more high-quality NMR analyses of dimers were available, the TMD sequences all showed strong sequence homology to an existing TMD in the dataset and could not be included. The self-interacting TMDs of bitopic proteins examined by ETRA and NMR studies (29 in total) were deemed insufficient for an extensive analysis of interface residue properties. A third dataset was therefore created by identifying 21 self-interacting and parallel TM helices derived from the structural database PDBTM [67]. This “X-ray” dataset was primarily derived from experimental crystal structures but included three high-resolution structures derived from electron microscopy. Most TMDs in the X-ray dataset correspond to identical TMDs from polytopic subunits whose interaction supports the latter’s non-covalent homo-oligomerisation. The X-ray dataset also contains two bitopic proteins (TMDs 32 and 34, Table 1). Interface residues in the NMR and X-ray structures were then defined using a 3.5 Å cut-off in closest heavy-atom distance. In all datasets, interfaces were found to follow an α-helical pattern (Fig. S5).

In total, the database of 50 TMDs contains 1091 residues, of which 304 are interface residues and 787 are non-interface residues (mean = 6.1 interface residues per dimer, equivalent to 28% of the total residues). The complete dataset is non-redundant at the 20% and 40% amino acid identity level for the full-length and TMD sequences, respectively. The structural TMD dimers show a high level of symmetry. In the NMR and X-ray structures, 25% and 27% of the interface residues contact the same residue (i,i contact) or a direct neighbour (i,i + 1) in the opposite chain (Fig. S6), respectively.

### 2.3 Interface residues tend to be conserved, polar, coevolved, and centrally located

**Fig. 2:**
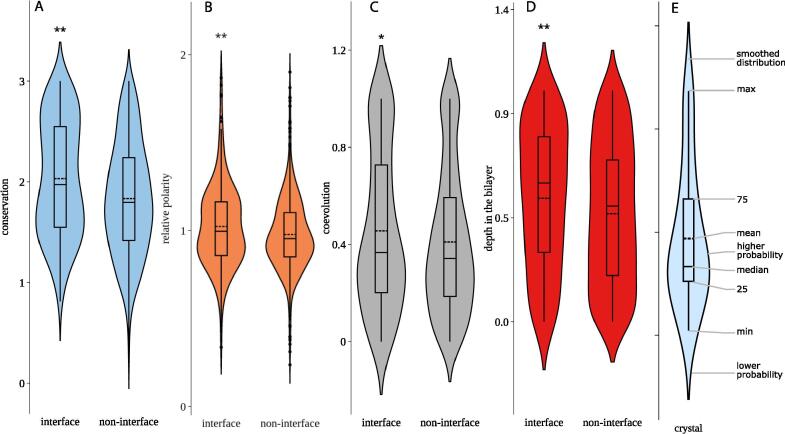
Interface residues exhibit higher conservation, coevolution, relative polarity and depth in the bilayer than non-interface residues. (A) Conservation. (B) Relative polarity. (C) Coevolution (DImax; see Text S1). (D) Depth in the bilayer. (E) Components of the violin plot. Statistical significance was measured using a bootstrapped t-test (*, p<0.05. **, p<0.01).

The evolutionary conservation of residues was calculated from multiple sequence alignments (MSAs) against homologues. For interfacial residues, the average conservation is significantly higher than that of their non-interface counterparts (Fig. 2A*,* p < 0.00001, Student’s *t*-test). Note that the conservation data and most other data examined herein had a non-normal distribution. To obtain more accurate estimations of statistical significance, all *p*-values in this study were calculated using bootstrapped data. The strong difference in conservation between interface and non-interface residues shows that interfaces are less likely to change during evolution than the remainder of a TMD. Although this finding seems intuitive, it contrasts with studies of PPI interfaces in soluble proteins, where a higher conservation at interfaces has been disputed [68], and may only exist in selected conditions [69]. We also found that the interface residues are distinguished by high polarity relative to the surrounding six residues (relative polarity, Fig. 2B; p = 0.0014).

Although these are all true PPI interfaces, the importance of conservation and polarity shown here is consistent with the known importance of these factors for polytopic membrane protein folding [9], [10], [11], [12], [47].

Another feature that is a strong predictor of polytopic membrane protein folding is residue coevolution [70], [71]. On the assumption that coevolution is also a predictor of PPI interfaces, it has been previously suggested that residue coevolution can also help predict homotypic TM interfaces [43]. Here, we tested a number of different measures of coevolution (Text S1) that do not require *a priori* knowledge of the interface and are thus termed “predictive” measures. Briefly, pairwise mutual information (MI) and direct information (DI) scores were calculated from multiple sequence alignments (MSAs) using EVfold [14], [70]. We then developed several different coevolution measures that comprised the mean or maxima of different pairwise coevolution values. Note that unlike the approach taken previously [43], we did not use the pairwise coevolution values directly. For example, the coevolution measure “DImax” determines whether the residue-of-interest has a very strong signal with some other (unspecified) residue in the TMD. As another example, the “DImean” determines whether the residue-of-interest has a high average coevolution with its immediate neighbouring residues. The advantage of such residue-specific rather than pair-specific coevolution values is that they are easily incorporated into a machine-learning algorithm that takes residue properties as an input. When both normalised and raw values were taken into account, a total of 52 coevolution measures were tested, of which DImax is used as an example in the respective figures. DImax is simply the maximum coevolution value between the residue of interest and all other residues in the TMD. The DImax is typical of many DI coevolution features in that it was slightly higher for interface residues in comparison to non-interface residues (Fig. 2C, Student’s *t*-test, p = 0.031). Overall, 34 of the 52 coevolution features differed significantly between interface and non-interface residues (Student’s *t*-test, p <= 0.05, Table S1), with the most significant difference seen for DItop4mean (p = 0.0013). Typically, DI values were higher at interfaces, while MI values were lower (Table S1). This could reflect the fact that the MI values are artificially low at positions of high conservation (Fig. S7). We also noticed that MI values and their distribution in the TMD were affected by the number of homologues (Fig. S8). Due to these effects, it is difficult to compare DI and MI values between different TMDs. We therefore normalised the DImax values in the statistical analyses. As detailed below, however, we included both raw and normalised values of all coevolution features in the initial machine-learning analysis.

A previous study compared DI values of pairs of known interface residues and pairs of non-interface residues [43] (see: Fig. S6A). Since this approach requires *a priori* knowledge of the interface, we refer to it here as a “retrospective” coevolution analysis. We emphasise here that the metrics used for retrospective analyses cannot be used for interface prediction. In a detailed analysis of retrospective coevolution (legend to Fig. S6), we found it difficult to confirm whether pairwise coevolution scores are higher between interface residues than between non-interface residues as previously described [43]. Instead, we found that the retrospective method used previously is biased by the non-random distribution of interface residues. Simply put, homotypic interfacial residues are often neighbours (Fig. S6) and neighbouring residues have high coevolution scores [14], [70], [72]. The coevolution of any residues that are close to each other in the sequence (interface or not) will always appear high, even if this group of “interface-like” residues is chosen randomly (Fig. S6). In predictors of interacting residues within polytopic membrane proteins, this effect is avoided by focusing on “long-range” interactions between residues that are spatially close in the 3D structure, but distant in sequence [16]. For self-interacting TM helices there are no such long-range interactions. Until a mathematical framework is developed to remove the “neighbour effect,” the proposed higher coevolution of interface residues in retrospective analyses can neither be proven nor disproven. In contrast, the predictive coevolution measures used here are free of the neighbour effect, as they do not rely on a particular distribution of interface residues. Therefore, the moderately higher DI measures at interfaces shown here (Fig. 2C, Table S1) provide the first evidence of enhanced coevolution between homotypic TMD interface residues.

**Fig. 3:**
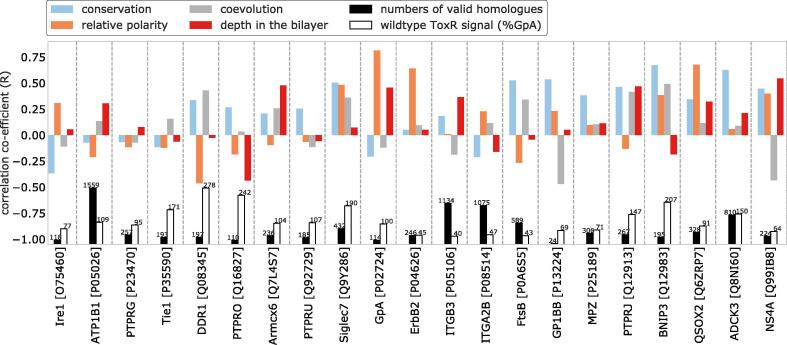
The properties of interfaces are highly TMD-specific. Data is shown for the ETRA dataset. The correlation co-efficient (R) indicates the relationship between the importance for self-affinity (i.e. disruption) and the relevant residue property, within the data for that TMD. R values are shown rather than R^2^, in order to indicate the direction of the correlation. The number of valid homologues in the respective MSA and the relative affinity of the wildtype homodimer is shown in the lower section of the graph. Note that the TMDs of the ETRA dataset tended to have few homologues (Fig. S11). As a result, coevolution (e.g. DImax, shown here) tended not to be highly indicative of interfaces.

Separate analyses of the ETRA, NMR, and X-ray sub-datasets confirmed the general trends given in Fig. 2 (Fig. S9, Fig. S10). We show that the preferential coevolution of interface residues is strongest for TMDs of the X-ray dataset. We attribute this to the relatively high number of available homologues (Fig. S11), a factor known to improve the usefulness of coevolution values [14], [70], [73]. In fact, the bitopic proteins in the ETRA and NMR datasets often contained few valid homologues (Fig. 3, Fig. S11), which presumably increased the variability of all evolutionary features.

The large dataset of TMDs also helped offset the high variability that we observed in the evolutionary data, possibly attributable to the small number of valid homologues found for many TMDs of bitopic proteins (Fig. 3, Fig. S11).

A new feature discovered in this study is that interfacial residues also tend to be located deeply in the membrane (Fig. 2D; p = 0.002). The “depth in the bilayer” (feature name: residue_depth) has not previously been examined for homotypic TMD interfaces. The importance of the depth in the bilayer was particularly noticeable in the ETRA dataset (Fig. S4B), whose data were sourced from experiments in a natural membrane environment.

A different way of presenting the data shown in Fig. 2 is to calculate the percentages of TMDs where the mean value of a given property is higher for interface *vs*. non-interface residues. This method minimises the biases of TMD-specific variables such as TMD lengths, overall conservation, and overall polarity. Accordingly, the interface residues of most TMDs in the homotypic TMD dataset had higher interface conservation, coevolution, relative polarity and depth in the membrane than non-interface residues of the same TMD (Fig. S10A). The situation is similar when the sub-datasets were analysed separately (Fig. S10B). The results confirm the trends in Fig. 2 but highlight a strong individuality in interface properties between different TMDs.

To understand this high variance, we examined the interface residue properties of individual TMDs more closely. In one approach, we calculate the correlations between the properties of individual residues and their role at an interface, as defined by the disruptive effect of mutations on self-interaction (Fig. 3). We restricted this analysis to the ETRA dataset because the disruption by mutation provides a graded and more direct measure of residue importance than the heavy-atom distances used to classify interface residues from NMR and X-ray structures. The results confirm a strong variability in interface residue properties between different TMDs. Most correlation coefficients are positive, confirming the overall trends established in Fig. 2. In another approach, we compared the average values of residue properties for interface and non-interface residues for individual TMDs. Evidently, some interfaces display highly elevated values, while interface/non-interface differences are small in other cases or even inverted (Fig. S12). We also visualise properties of individual interface residues in heatmaps for each TMD (Fig. S13) which confirms the differential contributions of conservation, coevolution and relative polarity to TMD-TMD interfaces of different biological functions.

### 2.4 Gly, GxxxG motifs, and strongly polar amino acids are over-represented at interfaces

**Fig. 4:**
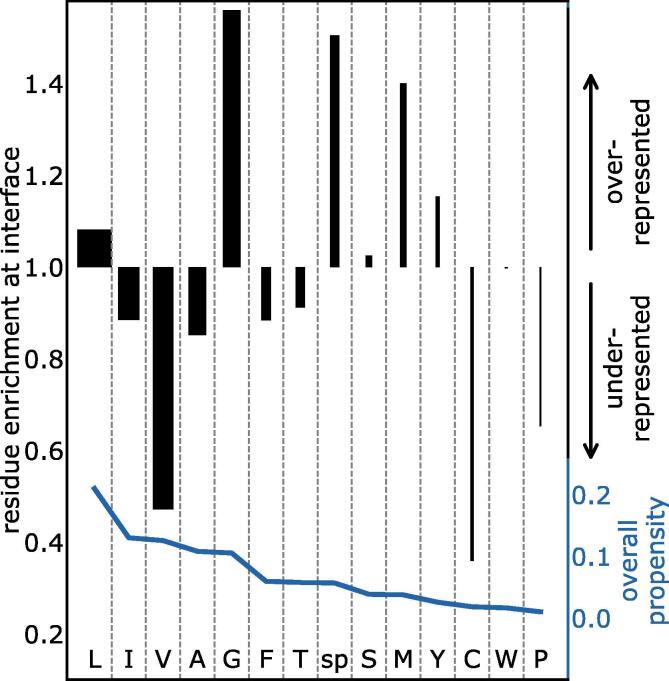
Interfaces are enriched in Gly, strongly polar residues, and Met, and are deficient in Val, Ala, and Phe. An analysis of the residues in the homotypic TMD dataset was conducted to detect the enrichment of particular residues at interfaces (black bars). The strongly polar residue types (sp = Asp, Glu, Lys, Arg, Asn, Gln, and His) were combined, due to lack of data when analysed individually. The residue enrichment at the interface equals the proportion of the residue type at interfacial positions, divided by the proportion of the residue type within all TMD sequences. In this analysis, residues with values much larger than 1.0 (e.g. Gly) are proposed to be enriched at interfaces, and therefore to drive homotypic TMD interactions. Residues with values much lower than 1.0 (e.g. Val) have a lower than expected abundance at interfaces, and therefore do not typically drive TMD interactions. Residues with values close to 1.0 are neither over- nor under-represented, and therefore drive TMD interactions no more than expected based on their abundance in the TMDs. The accuracy of this analysis is heavily dependent on the amount of data available for each residue, represented by the overall propensity in the TMDs (blue line and also bar-chart width). Strong conclusions should only be drawn for residues with a high overall propensity.

The residues Gly, strongly polar residues (Asp, Glu, Lys, Arg, Asn, Gln, His), Leu, and Met were found to be enriched at interfaces (Fig. 4). The enrichment of strongly polar and Gly residues is most pronounced for interfaces of the ETRA dataset (Fig. S14).

**Fig. 5:**
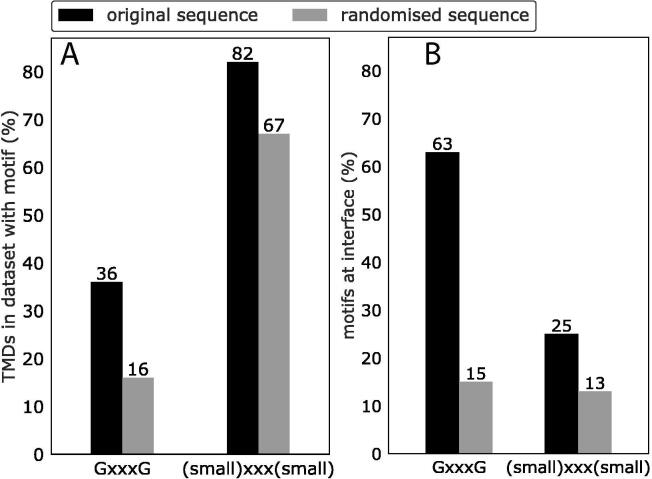
The GxxxG motif is not only overrepresented in TMDs, but also at interfaces. (A) Motif abundance in the TMD sequences of the homotypic TMD dataset. The higher value (black) in comparison to random (grey) shows that GxxxG and (small)xxx(small) motifs are more abundant than expected based on the proportion of these residues in the sequences. (B) Motif abundance at the experimentally determined interfaces of the homotypic TMD dataset. The bar shows the percentage of motifs where both residues reside at the interface. The much higher value (black) in comparison to random (grey) shows that GxxxG motifs are found at interfaces at a much higher rate than expected by chance and are powerful drivers of homotypic TMD interaction. This trend was much weaker for (small)xxx(small) motifs, which include GxxxG motifs. This finding suggests that (small)xxx(small) motifs in general are not a powerful indicator of homotypic interfaces of natural TMDs, and that much of their abundance at interfaces can be attributed to glycines or the GxxxG motif. To obtain the abundances expected by chance, random sequences were created with the same amino acid propensity and length as each original sequence. The mean result for 100 randomised sequences is shown. Values higher than in the randomised control show that the motif is overrepresented.

It has been long speculated that GxxxG motifs are over-represented at homotypic TMD interfaces. This hypothesis is based on early studies that firstly showed that the GxxxG motif drives GpA dimerisation [24], and furthermore that the GxxxG motif is found in single-pass proteins far more often than expected by chance [34]. Our dataset was no exception. GxxxG motifs were indeed more abundant in the TMDs than would be expected based on the percentage of Gly residues (Fig. 5A*)*. However, we show for the first time that GxxxG motifs are also more abundant at homotypic TMD interfaces than would be expected by random chance (Fig. 5B). Of all GxxxG motifs, 63% are interfacial, which is far above the proportion expected by chance (15%, Fig. 5B). On the other hand, the usefulness of the GxxxG motif as a general predictor of self-interaction or interface location is clearly limited. Most TMDs do not contain the motif. Furthermore, half of the GxxxG motifs in our dataset are not found at the interface, consistent with many case studies where the motif did not support TMD-TMD interaction [36], [74]. Enrichment of the GxxxG motif at interfaces is seen to a greater or lesser extent in TMDs of all subsets, whether investigated by ETRA, NMR, or X-ray crystallography techniques (Fig. S15). The overabundance of GxxxG motifs in the interfaces of the X-ray dataset is especially important, because these self-interacting helices were chosen without any human bias towards the presence of the motif. In contrast, the literature suggests that some TMDs were chosen for previous ToxR or NMR analysis partially due to the presence of the GxxxG motif. Interestingly, the more inclusive (small)xxx(small) motif exists within 76% of our sequences but is barely overrepresented in TMD sequences (Fig. 5A) or at their interfaces (Fig. 5B).

Consistent with the preeminent role of Gly at interfaces, sequence positions that are occupied by Gly TMD tend to be conserved, polar, co-evolved and located deep in the membrane (Fig. S16).

### 2.5 Development of THOIPA for interface prediction

Experimental investigation of self-interacting TMDs is difficult. In many studies, the available resources were sufficient to test the role of only a few selected residues in the interaction. There is therefore a strong need for algorithms to help predict such key residues, and to assist in the modelling of putative TM homodimer structures. We therefore developed a Transmembrane HOmodimer Interface Prediction Algorithm (THOIPA), which was trained as a classifier to predict the “interface” or “non-interface” designation of the residues derived from the homotypic TMD dataset.

THOIPA uses extremely randomised trees [75], an ensemble technique similar to a random forest. As input, THOIPA requires only the sequences of the TMD and of the full-length protein. As there are no other quantitative studies showing which residue features are important for homotypic TMD interfaces, we gathered a number of features that might be useful, including the proportion of each amino acid in MSAs against homologues (position specific scoring matrix, PSSM), and several variants of conservation, polarity, and coevolution (Fig. S17, Table S1). When roughly grouped by type, there were 52 features related to coevolution, 25 related to the PSSM, eight features related to conservation, eight features related to polarity, five features related to residue position or TMD properties, three features related to motifs (e.g. GxxxG), and two features related to physical properties of the corresponding residue (e.g. branched). Feature reduction was applied as described in the methods (Text S1), resulting in 27 features than were used for prediction and validation. The train data comprised 40 TMDs, and test data comprised 10 TMDs. The algorithm was tuned by automatically splitting the train data into further train/validation subsets, and choosing the parameters associated with the highest average precision score. Validation procedures included cross-validation within the train data, and blind-validation against the test data. THOIPA performance validation was conducted using three methods that focus on precision.

The THOIPA output score for each residue represents the probability that it lies at a homotypic interface. The algorithm is highly economical. For a TMD of interest, homologue downloads, feature extraction, and THOIPA prediction takes only few minutes on a standard office computer. A webserver (www.thoipa.org) and dockerised standalone software is available.

Depending on the method used to measure feature importance, either the GxxxG motif or the PolarxxxPolar motif were the most important features for THOIPA prediction (Fig. S17). Also important were several different features related to conservation, coevolution, and the absence of branched amino acid residues (e.g. V, branched).

To our knowledge, there are no other comparative algorithms designed to predict the most likely interface residues of self-interacting TMDs. There are, however, well-established algorithms designed to automatically predict TM homodimer structures, such as PREDDIMER [40] and TMDOCK [44]. We therefore analysed the top-ranked predicted structure from these algorithms, inferred interface residues based on heavy-atom distances using the same methods as applied to experimental structures, and validated them alongside THOIPA as predictors of interface residues.

**Fig. 6:**
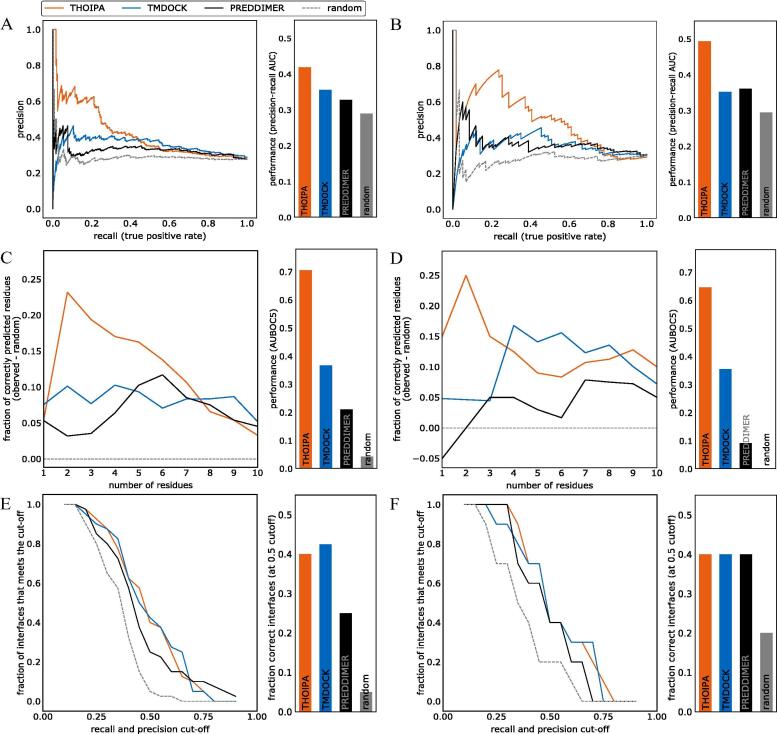
Performance validation reveals that THOIPA is a powerful predictor of the most important residues homotypic TMD interaction, but only weakly predicts the role of a larger number of residues. Validation data is shown for cross-validation (plots (A), (C), and (E)) and also blind-validation against a test-dataset (plots (B), (D), and (F)). (A, B) Precision-recall curve. The higher values at the left side of the chart show that THOIPA is far superior at identifying the small number of most-important residues in the dataset driving TMD interaction. The barchart shows the area under the precision-recall curve. Higher values indicate better performance, when all cut-offs are regarded equally. (C, D) Performance according to best overlap (BO) validation, a method developed here to report the number of residues at which peak performance is obtained (see Text S1). The line-chart shows the data for the top 10 residues according to the predictor, and the bar-chart shows the area under the curve for the top 1-5 residues (AUBOC5). The fraction of correctly predicted residues is analogous to precision. Higher values indicate better performance. As with (A), the higher values at the left side of the chart show that THOIPA excels at identifying the small number of most-important residues driving TMD interaction. (E, F) Fractions of interfaces that meet cut-off for precision-recall (FIMCO-PR) as per Lensink & Wodak of CAPRI [52]. Precision-recall plots were made for each TMD separately. The x-axis indicates the cutoff value for both precision and recall that was applied. The y-axis indicates the fraction of TMDs whose precision level was above this cutoff. For example, around 40% of the TMDs in cross-validation submitted by THOIPA had recall and precision levels higher than 0.5, but only around 10% of TMDs had recall and precision higher than 0.7. The barchart corresponds to the y-value where x equals 0.5. Note that the predictive power of TMDOCK is slightly over-estimated in all the above analyses, due to the automated truncation of some longer TMDs [44].

Validation revealed that THOIPA is vastly superior to TMDOCK and PREDDIMER for predicting the small number of most important residues in the interaction (i.e. high precision, Fig. 6A, B). This effect was seen for all TMD subsets, regardless of the experimental method used to define the interface (Fig. S18). In the assumption that users of THOIPA are only interested in the top 5 predicted interface residues, we developed our own “best-overlap” (BO) validation method (see Text S1). BO-validation somewhat resembles algorithms from the field of information-retrieval used to measure performance of internet search engines, such as precision@k, but also takes into account the high random precision associated with short, ~20-residue TMDs. BO-validation showed that THOIPA performance peaked when the top two residues from the predictor were considered (Fig. 6C, 6D). The overall performance for the top one to five residues (AUBOC5) was far higher for THOIPA than the other algorithms tested. THOIPA performance did not greatly differ between cross-validation (6*A,* 6*C,* 6*E),* and blind-validation (6*B,* 6*D,* 6*F)*, confirming that overfitting has been successfully avoided.

In predicting the entire interface region, THOIPA showed modest performance. To measure overall performance in predicting all interface residues, we adopted a Critical Assessment of Predicted Interactions (CAPRI) method developed by Lensink and Wodak [52]. The fraction of interfaces that meet the cut-off of precision recall (referred to here as FIMCO-PR) method revealed similar performance for THOIPA and TMDOCK. In general, the newer TMDOCK algorithm predicted interfaces better than PREDDIMER (Fig. 6, Fig. S18).

In the CAPRI study [52], a precision-recall cutoff of 0.5 demarked a successfully predicted interface. At this cutoff, THOIPA and TMDOCK correctly predicted over 40% of all interfaces in the train and test data. By comparison, the best automated predictor of soluble interfaces, HADDOCK, had correctly predicted a fraction of only 0.38 of 20 CAPRI targets [52]. Thus, the performance of THOIPA and TMDOCK is comparable to that of automated predictors of PPI in soluble proteins [52], [53], despite the challenges associated with the membrane environment and the severe paucity of experimental data. We also compared THOIPA to the simple LIPS algorithm [47] using a more rigorous MCC validation than the “percentage of native contacts” applied previously [43]. THOIPA clearly out-performed LIPS (Fig. S19). Nevertheless, we could confirm that the simple combination of conservation and polarity in LIPS works surprisingly well for many TMDs. Accordingly, features derived from LIPS often differed between interface and non-interface residues (Table S1) and proved useful as THOIPA features (Fig. S17).

THOIPA clearly achieved its goal by providing an objective predictor of homotypic TM interface residues to guide wet-lab experiments or energy-based modelling approaches. However, a validation of predictions for each TMD individually (Fig. S20) clearly shows that all fully-automated algorithms tested in this study give highly inconsistent results. Further understanding of TMD interfaces is necessary in order to enable any de-novo prediction of protein function.

## 3 Discussion

This study represents the most comprehensive analysis to date of homotypic TMD-TMD interfaces. Overall, we find the PPI interfaces shared many properties with the TM interfaces in folded polytopic membrane proteins. For membrane proteins, this suggests that there is a strong overlap between the forces and mechanisms underlying both PPI and protein folding. Of the numerous features that have been previously associated with homotypic TMD interfaces in case studies and artificial selection [3], [36], [74], [76], we only find evidence for a select few.

The lack of experimental data seriously impedes our understanding of homotypic TMD interfaces. Here we show that classical ToxR-based methods can be used to determine more novel homotypic TM interface residues than any previous single study. Importantly, we also show that datasets from multiple sources can be combined, and that the TMD interfaces typically share the same trends, regardless as to whether the experimental data was derived from ETRA (ToxR-like), NMR, or X-ray crystallography techniques.

We report for the first time the statistical overabundance of the GxxxG motif at natural homotypic TMD interfaces. This has been long suspected, ever since the GxxxG motif was shown to occur more often in TMD sequences than expected by random chance [34]. In addition to the GxxxG motifs, that do not suffice as predictors of TMD interfaces [36], we describe a number of other predictive features, including conservation, polarity, strongly-polar residues, co-evolution, depth in the membrane, (small)xxx(small) motifs, and a lack of β-branched residues. Of all these features, however, the GxxxG motif remained the strongest predictor of a homotypic TM interface. Nevertheless, the contribution of these factors to different interfaces is highly diverse and we emphasise that the structural individuality has been under-appreciated in previous studies.

Why are Gly residues so important? We found that sequence positions occupied by Gly residues are particularly well conserved, coevolved, and prefer a deep location in the bilayer. Based on the importance of polarity and residue-depth for interaction, we propose that the dominant role of the medium-polarity Gly in TMD interactions results from the best trade-off. Specifically, the structure of Gly may endow these residues with the most favourable contribution to helix-helix interaction [19], [20] for the least disruption to membrane insertion. All in all, these findings and interpretations are in line with what is known of the mostly heterotypic TMD-TMD interfaces that support the folding of polytopic membrane proteins [9], [47], [77], [78], [79].

The under-representation of Ile and Val at homotypic interfaces is in good agreement with a recent genetic screen of artificial self-interacting TMDs [80]. Possibly, the restricted side-chain mobility of these β-branched amino acids makes them less suitable to form a densely packed helix-helix interface, compared to the highly flexible side chains of the over-represented Leu or Met [81]. It should be noted that this contrasts an earlier view where Val had been identified as an interfacial residue of the GpA TMD dimer [25]. There, it had been speculated that the restricted side-chain mobility of Val might limit the entropy loss associated with the fixation of side chains within a helix-helix interface. However, mutation of these Val residues tends not to disrupt the self-interaction of GpA in biological membranes [5], [7], arguing against a central role in the interaction.

Our analysis shows for the first time a clear bias for interface residues to lie at the centre of the membrane. Conceivably, this reflects the fact that the points of closest helix-helix contact tend to localise near the centre of the acyl chain region. There, polarity drops to a minimum, thus optimising the contribution of polar forces to an interface [20], [82]. The central position in the TMD may also preventing the snorkelling of polar residues to the water–lipid boundary. According to this theory, we would expect that polarity is a poor predictor of PPI interfaces in juxtamembrane regions, where residues are free to interact with either water or lipid molecules.

Our data supports the hypothesis of Wang and Barth [43] that residues coevolve within a homotypic TMD-TMD interface. However, we argue that the retrospective scoring method used in the previous analysis has over-estimated the preferential coevolution of interface residues. Instead, our unbiased predictive coevolution measures show only a modest increase in coevolution scores at interfaces. Why would coevolution scores be such weak predictors of homotypic TMD interaction, when they so strongly predict contacts in the field of membrane protein folding? We attribute this difference to several issues specific to TM homodimer interfaces: (i) Coevolution can only be calculated for pairs of non-identical residues. It cannot detect the contribution of pairs of identical residues [2], [3], [83], which made up 25% of the interface contacts in the NMR and X-ray datasets. (ii) There is a high background of co-evolution between neighbouring residues in the sequence (Fig. S6). This background is particularly relevant to the highly symmetric homotypic TMD interactions. In contrast, the heterotypically interacting residues that determine the folding of polytopic membrane proteins are distant in sequence but close in spatial proximity, which enhances the value of their coevolution scores for fold prediction [16], [70], [84]. (iii) There is a high background of coevolution between residues on the same side of an α-helix, visible as peaks at spacing of i,i + 4 and i,i + 7, as shown here for TMDs (Fig. S6) and previously for soluble helices [72]. While this sidedness of coevolution might relate to interface formation, part of it might reflect side-chain/side-chain interactions determining the conformational flexibility of TMD helices [85].

Our machine-learning predictor, THOIPA, is the first of its kind for predicting homotypic TMD interfaces. Machine learning is already a common technique applied to related problems, including the prediction of PPI interface residues between membrane proteins with a known structure [49], [50], [51], or the prediction of contacting residues within a folded polytopic membrane protein [16], [84], [86]. THOIPA is well-placed to prioritise TMD residues in mutational analyses of given functions, assuming that they contribute to quaternary structure formation. A further advantage of THOIPA is that it is completely agnostic to the oligomerisation state, which is usually unknown. An interesting question for future studies is how evolutionary predictors such as THOIPA or EFDOCK-TM [43], and energy-based predictors, such as TMHOP [46], TMDIM [45], or TMDOCK [44] can be most effectively combined to improve the blind prediction of interface residues and oligomeric structures. For interface prediction, we present two rigorous methods by which models can be validated. Firstly, the AUBOC5 measures the ability to predict the top 5 residues involved in the interaction. Secondly, the FIMCO-PR measures the ability to predict a larger number of interface residues. Although the prediction power of all currently available algorithms appears modest, the accuracy of machine-learning predictors, such as THOIPA, will increase with the size of the training set, which is certain to rise in the future. In addition, since the accuracy of coevolution measures strongly depends on the number of homologues, the performance of THOIPA will also benefit from the exponential increase in publicly available sequence data.

## 4 Materials and methods

### 4.1 ToxR assay

The ToxR reporter assay in *E. coli* was conducted as previously described [5], [23]. Single amino acids were mutated using Q5 site-directed mutagenesis (NEB). All residues in the TMD were initially mutated to Ala, except for positions containing Gly or Ala, which were mutated to Ile. Further mutagenesis was done at mutation-sensitive positions, as identified in the first round of scanning mutagenesis. Disruption (*d*) to dimerisation for each mutation was measured as follows:

(1) $$d=\frac{w-m}{w}$$

where *w* is the dimerisation signal measured for the wildtype TMD, and *m* is the dimerisation signal for the TMD containing that particular mutation. The disruption at a residue position was measured as follows

(2) $$\overset{¯}{d}=\frac{\sum d}{n}$$

and consisted of the mean disruption for all available mutations at that position. Full details are in Text S1.

### 4.2 The homotypic TMD dataset

The ETRA dataset includes new scanning mutagenesis data from this study (9 TMDs), and previous ETRA scanning mutagenesis data from the literature (12 TMDs). The initial NMR dataset consisted of the 13 default dimer structures included in the validation by Wang et al. [43], plus the recently published TM dimer structure of toll-like receptor 3 (PDB 2mk9, UniProt O15455, ref. [87]), and death receptor 5 (PDB 6nhw, [88]). Proteins already present in the ETRA dataset were not considered. Interacting residues were defined as residues that contain a pair of heavy (non-hydrogen) atoms, one from each amino acid, being less than 3.5 Å apart. The use of heavy-atom distances is a standard method to define contacting residues and has been previously applied in a study of PPI in membrane proteins [51], and indeed most of the case studies that comprised the NMR dataset. Interface positions were defined as TMD residues that interact with any other TMD amino acid in the opposing helix. The X-ray dataset consists of self-interacting TM helices extracted from crystal structures or high-resolution electron microscopy. The dataset “Non-redundant alpha” was downloaded from PDBTM [67]. Structures with a poor resolution (above 3.5 Å) were excluded. Interface residues were identified as described above for the NMR TMDs. Only self-interacting TM helix pairs that had at least four interface residues were retained.

The “homotypic TMD” dataset consists of the combined ETRA, NMR and X-ray structure datasets. The homotypic TMD dataset was non-redundant at the 20% amino acid identity level for the full-length sequence. The helices in the X-ray dataset not only interact homotypically, but also with other chains or proteins in the membrane-protein complex. This contrasts with the ToxR/NMR data, where the residues are either involved with homotypic interactions, or lipid interactions. We therefore split the TMD residues of the X-ray dataset into three groups: (A) residues involved in TMD self-interaction (126 residues), (B) residues assumed to be in contact with lipids (306 residues), and (C) residues involved in non-homotypic TMD interactions (i.e. protein folding, 47 residues). Residues in group (C) were determined objectively based on heavy-atom contacts, exactly as for group (A), as described in detail in the methods (Text S1). Our statistical analyses comparing interface and non-interface residues examined the properties of (A) against (B), and ignored group (C). Similarly, the THOIPA machine-learning algorithm was trained on a dataset that excluded group (C). Unlike the statistical analyses, however, all validation of prediction algorithms was carried out by considering group (A) as interacting residues, and groups (B) + (C) as non-interacting residues. As the goal of THOIPA is the prediction of interface residues in interacting TMDs of bitopic proteins, in theory THOIPA could also be validated in a dataset that excluded group (C). However, we included group (C) in THOIPA validation in order to allow a fair comparison with the structural algorithms TMDOCK and PREDDIMER, for which the exclusion of single residues is not possible. Similarly, the residues of group (C) could not be excluded in our analyses of motif abundance, for which the non-interacting residues were assumed to be groups (B) + (C). Further details on the methods are available in Text S1.

### 4.3 Calculation of residue properties

A total of 103 residue features (properties) were extracted from the TMD sequences and evolutionary data. Homologues were obtained by BLAST against the NCBI non-redundant dataset using the TMD plus 20 surrounding residues as the query. Conservation was based on Shannon entropy, but inverted to yield positive values that increased with a decreasing rate of evolution. In other features, conservation was based on the result from rate4site [89]. Polarity was calculated using the GES scale [90] and corresponds to the mean hydrophobicity at that position of the MSA. Relative polarity was the polarity score of a particular position, relative to the surrounding six residues. Residue depth refers to the relative position of the residue in the TMD, which range from 0 (first or last residue) to 1 (central residue). Coevolution features were calculated based on the FreeContact implementation [91] of EVfold [15]. Further residue properties are detailed in Text S1.

### 4.4 Machine learning and evaluation

THOIPA is a machine-learning classifier that uses extremely randomised trees [75], a method distinguished by high performance and interpretable feature importances [92]. Of the 50 TMDs in the homotypic TMD dataset, 40 were used as train data, and 10 were used as blind test data (2j58A1, 3zk1A1, 4ryiA2, 5nkqA1, P20963, O15455, O75460, P08514, Q12983, and P05026). TMD homodimer structure predictions from PREDDIMER and TMDOCK were obtained by submitting the TMD sequence to the relevant online server. The top ranked structure according to the respective algorithm was used for validation. Full details are in Text S1.

### 4.5 Statistical significance

Pairwise comparisons were conducted using an independent Student’s *t*-test assuming equal variance. To allow for comparison of data with non-normal distributions, *t*-tests were conducted on bootstrapped data. *P*-values were represented as follows: *, *p* < 0.05. **, *p* < 0.01, ***, *p* < 0.001.

## Author contributions

Y.X., B.Z., D.F., D.L., and M.G.T designed research; Y.X., B.Z., M.G.T and N.B. performed research; Y.X., B.Z., D.F., D.L., and M.G.T wrote the paper.

## Funding

This work was supported by 10.13039/501100001659Deutsche Forschungsgemeinschaft (grants La699/13_2 and FR 1411/14_1) and the Center for Integrated Protein Science Munich (CIPSM). Y.X. and B.Z. were each a recipient of a China Scholarship Council Postgraduate Research Scholarship.

## Competing interests

The authors declare no conflict of interest.

## CRediT authorship contribution statement

**Yao Xiao:** Conceptualization, Investigation, Methodology, Visualization. **Bo Zeng:** Conceptualization, Investigation, Methodology, Software. **Nicola Berner:** Investigation. **Dmitrij Frishman:** Conceptualization, Writing - review & editing, Supervision. **Dieter Langosch:** Conceptualization, Writing - review & editing, Supervision. **Mark George Teese:** Conceptualization, Methodology, Software, Visualization, Supervision.

## Data Availability

All data are accessible via a repository of the Open Science Foundation (https://osf.io/txjev/).
