# Supplementary material for: Experimental determination and data-driven prediction of homotypic transmembrane domain interfaces
Source: Comput Struct Biotechnol J. 2020 Oct 7;18:3230–42. doi: 10.1016/j.csbj.2020.09.035 (PMC7649602; doi:10.1016/j.csbj.2020.09.035)
Supplement: Supplementary data 1 [file mmc1.pdf]

Text S1. Supporting methods

ToxR assay and scanning mutagenesis

The ToxR reporter assay in *E. coli* was conducted as previously described [1, 2]. Cell volumes were either 5 ml in glass tubes, or 0.3 ml in 96-well plates as described for the BlaTM assay (see supplementary methods of ref. [3]). For novel TMDs with unknown dimer affinity, we measured the dimerisation in the ToxR assay of four consecutive, 20-residue sequence frames. Scanning mutagenesis was performed on the frame with the highest self-affinity. Single amino acids in the dimerising TMDs were mutated using Q5 site-directed mutagenesis (NEB). In several cases we used degenerate codons encoding multiple amino acids, such as the codon NRS, which yields an equal distribution of Pro, Leu, Ala or Val. For mutation-sensitive positions, we confirmed correct membrane insertion using the MalE complementation assay as previously described [2].
We first identified nine TMDs with strong self-affinity by testing those previously reported to have strong self-affinity in literature. We also measured the self-affinity of other human TMDs that had strong sidedness of conservation [4], and had not previously been tested in an ETRA assay. The former approach proved to be more successful and contributed eight from nine of the TMDs with high affinity that were investigated further by scanning mutagenesis. For the latter approach, ToxR assays revealed only moderate self-affinity for the TMDs with a high sidedness of conservation: Ire1 (77% of GpA), THSD7A (76% of GpA), SHISA9 (67% of GpA), CADM3 (63% of GpA), calnexin (56% of GpA), PLXDC1 (50% of GpA) and NDST3 (47% of GpA). Of the TMDs with a high sidedness of conservation, Ire1 was chosen for scanning mutagenesis.

Collection and normalisation of ETRA data from literature

We collected all data from previously published studies that aimed to identify natural TMD homodimer interfaces by disruptive mutations. We retained only TMDs with mutations in at least 75% of the TMD residues, including mutations in at least 15 consecutive residues. ETRA interface residues were defined as any position with a mean disruption value above 0.24. The ETRA dataset (and all other datasets in this study) were made non-redundant by clustering full-length protein sequences with CD-HIT [5] using an amino acid sequence identity cut-off of 40%. A second round of CD-HIT redundancy reduction was conducted based only on the TMD sequences, using a sequence identity cut-off of 60%.

The GALLEX assay had been used for the analysis of the NS4A TMD of Hepatitis C [6]. All other TMDs from the literature had been investigated with a ToxR-based assay. These included GpA [7], ErbB2 [7], BNIP3 [8], ADCK3 [9], QSOX2 [10], FtsB [11], integrin αIIb a.k.a. ITGA2B, [12], integrin β3 a.k.a. ITGB3 [13], GP1BB [14], MPZ [15], and PTPRJ [16]. The scanning mutagenesis values were either taken from supplementary data of the relevant article or calculated from the bar heights in figures. The dsTβL data for GpA and ErbB2 were kindly provided by Assaf Elazar, and correspond to Figure 5 of Elazar et al. 2016 [7]. We normalised the dsTβL ΔΔGapp association data by dividing by the highest value (L13T for GpA, I18G for ErbB2). The normalised values were considered equivalent to the fraction of wildtype activity obtained from other studies. In comparison to ToxR signals, the GALLEX signal is inverted and is typically presented using a logarithmic axis. To identify interface residues from GALLEX data, for each GALLEX mutation we took the cube root of the fraction of wildtype activity. We then normalised the data from 0 to 1, so that the pBR322 control was equal to 0, and the wildtype was equal to 1. In this way, mutations with higher dimerisation than the wildtype gave scores larger than 1. This resulted in the fraction of wildtype dimerisation for each mutation, which was further processed as for the ToxR data. For ITGA2B, the reported wildtype value was unusually high in comparison to values derived from mutations [12]. To obtain comparative values to all other studies, the signal for each mutation was

normalised to the median signal of all mutations, rather than to the wildtype. All data is available in the OSF data repository (https://osf.io/txjev).

Display of alpha helicity in figures

To examine the α-helical periodicity of the interface residues, we fitted the disruption index to a sinusoidal formula $y = a*\sin(bx+c)+d$ using the leastsq function of the scipy.optimize module. Fitting was conducted assuming perfect α-helicity (periodicity of 3.6) by keeping b constant at $2\pi/3.6$.

Identification of heterotypic contacts in X-ray dataset

The "homotypic TMD" dataset consists of the combined ETRA, NMR and X-ray structure datasets. As described in the methods, we identified 47 "heterotypic contacts" within the 21 TMDs of the X-ray dataset. Heterotypic contacts are known to have similar properties to the PPI contacts described here, such as high conservation, polarity, and a predominance of small residues [17-20]. The heterotypic contacts were defined based on heavy-atom distances to non-self TM helices using a 3.5 Å cut-off. Our primary aim was to better understand the properties of self-interacting TMDs from bitopic proteins, which do not have heterotypic contacts. To ensure that the X-ray dataset closely resembled the bitopic ETRA & NMR datasets, we removed the X-ray heterotypic contacts from all statistical analyses except motif analyses. We also removed them from the THOIPA training set. Heterotypic contacts were still included in all THOIPA validation procedures to allow a fair comparison between THOIPA and the other prediction algorithms tested here, as THOIPA is the only algorithm for which individual residues in a TMD can be excluded during prediction.

Calculation of a standardised "interface score" for each residue

To allow the relative importance of the residues to be directly compared between mutagenesis and structural experimental data (Fig S5, Fig S13), for each residue we calculated a normalised interface score. The interface score ranged from 0 (low importance for dimer) to 1 (high importance for dimer). For ETRA TMDs, the interface score was obtained by normalising data between disruption values of -0.4 and +0.4. The interface score was set at 0 for disruption values of -0.4 or lower, and set at 1 for disruption values of +0.4 or higher. For NMR and X-ray TMDs, the interface score was obtained by normalising the closest heavy-atom distances in an inverted fashion between 10 Å and 2 Å. The interface score was set at 0 for distances of 10 Å or higher, and set at 1 for distances of 2 Å or lower. For BO-validation and the analysis of interface helicity (Fig 6, Fig S18, Fig S5), it was necessary to identify the "most important" position for the dimer. For ETRA TMDs, we used the position with the highest average disruption. For NMR and X-ray TMDs, we used the position with the lowest heavy atom distance.

Sequence alignments and extraction of key predictive features

Sequence homologues were obtained via BLASTp against the NCBI non-redundant (nr) database. The BLAST query sequence consisted of the predicted TMDs plus 20 adjacent N- and C-terminal residues. BLAST was conducted using the relatively permissive default settings to recruit the largest possible number of candidate homologues, including those distantly related to the query sequence. The TMD in the match sequence was identified based on the alignment to the query. The number of false positive hits was reduced by keeping only the alignments with fewer than 6 gaps and at least 20% sequence identity in the TMD region. Homologues with gaps in the query sequence were excluded. The remaining sequences were used to derive a multiple sequence alignment (MSA) from the original BLASTp pairwise alignments. For lipophilicity calculations that required information outside the immediate TMD region, the TMD plus five surrounding residues were extracted from the alignments, and the filtering procedure repeated as described above. Based on a

careful manual verification of the resulting alignments we estimate that the number of false positives (i.e. non-homologues) in our data does not exceed 2%.

Calculation of residue properties

In this section we describe the calculation of physico-chemical, structural and evolutionary properties of amino acid residues at each sequence position (designated i) of transmembrane domains (TMD). In total, 103 features were considered. Some of the properties were derived from multiple sequence alignments (MSA). These were gathered by searching the NCBI non-redundant database for related sequences using BLASTp. Homologues were filtered by keeping only the alignments with fewer than 6 gaps and at least 20% sequence identity in the TMD region. Only homologues with unique TM sequences were retained (non-redundant to 100% sequence identity). In addition, we calculated position specific scoring matrices (PSSM) to quantify the evolutionary profile of each amino acid in a TMD. A PSSM contains the frequencies of all 20 amino acids in each MSA column.

Features based on residue coevolution

We employed the FreeContact implementation [21] of EVfold [22] to calculate coevolution scores between all possible residue pairs in the TMDs. For each residue pair, the EVfold output includes the values of mutual information (MI) and direct interaction (DI). Mutual information is a standard measure of coevolution between two residues but is known to be prone to several biases [22, 23]. For example, high scores can be seen for indirect contacts, e.g. when residues B and C are not in contact, but both make contacts and coevolve with residue A. A second bias in MI is the low score associated with high conservation (Fig S7). Direct coupling analysis (DCA) is a global statistical inference method that aims to disentangle direct and indirect contacts and counter the effects of high conservation. DCA yields an adjusted DI score for each residue pair. For prediction in THOIPA, and to understand interface properties, it was necessary to convert the pairwise coevolution scores to a single representative value at each residue position. We included 16 such coevolution measures, comprising nine MI and nine DI values, respectively. In all cases, the predictive coevolution value was the maximum or mean from a selected number of residue pairs that included the residue of interest.
Briefly, for a pair of residues *i and j,* MI *was* calculated as:

$$\mathrm{MI(i,j)}=\sum_{A_i,A_j=1}^{q} f_{ij}(A_i,A_j)\,ln\,(\frac{f_{ij}(A_i,A_j)}{f_i(A_i)f_j(A_j)})$$

where $f_{ij}(A_i,A_j)$ is the observed frequency of amino acid pairs A_i, A_j jointly occurring at positions i and j of an MSA, $f_i(A_i)$ and $f_j(A_j)$ are the overall probabilities of residue A at position i and residue A at position j, and q is the number of all possible residue pairs (A_i , A_j).

DI was calculated according to the following equation:

$$\mathrm{DI(i,j)}=\sum_{A_i,A_j=1}^{q} P_{ij}^{Dir}(A_i,A_j)\ln\,(\frac{P_{ij}^{Dir}(A_i,A_j)}{f_i(A_i)f_j(A_j)})$$

Here, the local pair probability f_{ij}(Ai,Aj) used in MI is replaced by the global pair probability $P_{ij}^{Dir}(A_i,A_j)$. The latter is calculated based on a global probability model using the entropy

maximisation approach, which calculates correlation scores for each pair of residues while considering all other pairs [22, 24, 25].
MItop{x}mean, DItop{x}mean. The coevolution scores (MI or DI) between the residue of interest and all other residues in the TMD were calculated and ranked from highest to lowest. The mean was then calculated for the top-scoring x residue pairs, where x is 4 or 8..
MI{x}mean, DI{x}mean. Mean coevolution (MI or DI) between two residue pairs, i and i+x, as well as i and i-x, where x represents a distance of 1-5 residues.
MI4value, DI4value. The MI or DI coevolution value between two residue pairs, i+4 and i-4, which lie approximately on the same side of an alpha helix adjacent to the central residue, i.
MImax, DImax. The maximum coevolution value (MI or DI) between the residue of interest and all residues in the TMD.
MI4_inclusive_max, DI4_inclusive_max. The maximum coevolution value (MI or DI) between the residue of interest and the eight neighbouring residue positions (i-4 to i+4).
MI4_inclusive_mean, DI4_inclusive_mean. The mean coevolution value (MI or DI) between the residue of interest and the eight neighbouring residue positions (i-4 to i+4).
MI4cum, DI4cum. The coevolution values (MI or DI) between all possible residue pairs in the TMD were measured and sorted from highest to lowest. All unique residues in the top 4 residue pairs were identified. A boolean value was then created, describing whether the residue of interest was among these residues.

Normalisation of coevolution-based features

The mean MI coevolution values were found to decrease with an increasing number of homologues (Fig S8). For both MI and DI, the standard deviation of the values decreased with the number of homologues (Fig S8). To minimise these effects, we normalised the coevolution features described above between 0 and 1 within each TMD. Statistical analyses were carried out using normalised coevolution data. Features using both normalised and raw coevolution data were used for machine learning. Features with raw coevolution data are denoted by the suffix "_raw".

Features based on homologues and evolutionary sequence conservation

n_homologues. The cubed root of the number of homologues in the MSA.
conservation. Shannon entropy ($S_{entropy}$) was inverted and converted to positive values as follows:

$$S_{entropy} = -\sum_{i=1}^{20} p_i \log p_i$$

$$\text{conservation} = -S_{entropy} + 3$$

where p_i represents the observed frequency of amino acid *i* in the given MSA column. This yields a conservation score based on entropy, which comprises only positive values that increase with a decreasing rate of evolution.
cons4mean. Mean conservation of the three residue positions i, i-4 and i+4.
rate4site. Conservation was also calculated using the rate4site algorithm [26]. The input MSA was used as described above, including the 20 residues before and after the TMD region. Rate4site is limited to a maximum of 200 sequences in the MSA, whereas BLAST yielded in many cases thousands of unique homologue sequences. Therefore, successive rounds of CD-HIT [5] at lowering thresholds were applied to remove redundant sequences until the MSA contained less than 200 sequences. The lowest CD-HIT cutoff applied was 0.2 (20% AA identity). For TMDs that still contained more than 200 homologues at this CD-HIT threshold, the rate4site input consisted of the first 200 sequences in the alignment. Rate4site values were multiplied by -1 to yield a conservation score.
rate4site4mean. Mean rate4site value of the three residue positions i, i-4 and i+4.

LIPS_surface. The binary LIPS output score [27].
LIPS_L*E. LIPS_polarity * log(LIPS_entropy)
LIPS_surface_ranked. LIPS_surface - (LIPS_L*E / 20). This yields the binary LIPS score, adjusted so that conserved and polar residue positions have a slightly higher score than poorly conserved and nonpolar residue positions, without overriding the original binary output.

Polarity

polarity. Polarity was calculated for each position in the MSA. The PSSM of amino acid frequencies was first adjusted to exclude gaps, ensuring that the sum of the amino acid frequencies was 1. The lipophilicity at each position (l) was the sum of the proportion of each residue type (p_r) multiplied by the respective value in the GES (Engelman) hydrophobicity scale (h_r) [28] as follows:

$$l = \sum p_r \, h_r$$

The GES scale was chosen as it offered consistently high performance during THOIPA development and validation. According to the GES scale, higher values correspond to higher polarity (e.g. positions rich in Lys or Glu). The final polarity (p) was calculated as follows

$$p = \sqrt[4]{-l + 3.7}$$

yielding positively charged values with a roughly normal distribution.
relative polarity. Polarity at position i divided by the mean polarity of the 6 surrounding residues (i-3 to i+3, excluding i).
polarity1mean, polarity4mean. Mean polarity of three positions i, i-x, and i+x, where x is equal to one or four.
polarity3Nmean, polarity3Cmean. Mean polarity of the 3 N-terminal and 3 C-terminal residues relative to the residue of interest, respectively.
LIPS_polarity. The polarity at each residue position, as calculated by the LIPS algorithm using an amino acid alignment against homologues as input.

Features based on presence or absence of helix interaction motifs

GxxxG. A boolean variable describing whether a given residue participates in a GxxxG motif.
SmxxxSm. A boolean variable describing whether a given residue participates in a (small)xxx(small) motif, with small residues defined as GASC.
PolarxxxPolar. A boolean variable describing whether a given residue participates in a (polar)xxx(polar) motif, with polar residues defined as DKERQPHNSGYTWAMC.

Features based on amino acid and di-peptide composition

A, C, D, E, F, G, H, I, K, L, M, N, P, Q, R, S, T, V, W, Y, CS, QN, KR, DE, LIV. The evolutionary profile (position-specific scoring matrix, PSSM) comprised the fraction of each residue at that position in the multiple sequence alignment (MSA). In addition, the propensities for residue groups with highly similar properties were combined, such as positively charged (KR), negatively charged (DE), strongly polar uncharged (QN), and large aliphatic (LIV) residues. For example, the feature "LIV" is the combined fraction of Leu, Ile, and Val residues at that position in the multiple sequence alignment.
mass. Mass of the amino acid in the TMD of interest, taken from AAindex [29].
branched. A boolean variable indicating whether or not a residue is classified as a β-branched amino acid, according to AAindex [29]. β-branched residues comprised Ile, Val, and Thr.

Features based on structural properties

residue depth. Relative position of the residue in the TMD, where 1 represents a central residue, and 0 represents either the most N-terminal or C-terminal residue. This was rounded to one significant figure (0, 0.1, 0.2 etc) to prevent machine learning methods from remembering exact residue positions, rather than learning general interface properties.

RelPos_TMD. Relative position of the residue in the TMD, where 0 represents an N-terminal residue, and 1 represents the most C-terminal residue, rounded to one significant figure as described above.

RelPos_fullseq. Relative position of the residue in the full protein sequence, where 0 represents the most N-terminal residue, and 1 represents the most C-terminal residue, rounded to one significant figure as described above.

n_TMDs. The number of TM helices in the full protein, as predicted by Phobius [30]. This variable can take the following values: 0, 1, 2, 3, and 4. For the training dataset of well-studied membrane proteins, the value 0 indicated an erroneous prediction by Phobius that the sequence encoded a soluble protein. The values 1, 2 and 3 represent the predicted number of TM helices, and 4 represents the prediction of 4 or more TM helices in the protein.

THOIPA parameters and validation

THOIPA was trained as a classifier using extremely randomised trees [31], a.k.a. extra-trees, as implemented in python (scikit-learn ExtraTreesClassifier). Extra-trees is a tree ensemble method similar to the popular random forest algorithm. Like the random forest method, extra-trees randomises the selection of *K* features at each node, from which the best is chosen for the split. However, extra-trees also randomises the cut thresholds for each feature, leading to increased variance within each tree, but improved performance for the ensemble of trees [31].

For validation, 10 of the 50 TMDs were randomly assigned as test data for blind-validation (2j58A1, 3zk1A1, 4ryiA2, 5nkqA1, P20963, O15455, O75460, P08514, Q12983, and P05026). The remaining 40 TMDs were used as train data and cross-validation. The proportion of TMDs investigated by ETRA, NMR, and X-ray techniques was roughly similar between the test and train data.

The training and validation pipeline included removal of duplicate features, feature reduction, hyperparameter tuning, crossvalidation of the train subset, calculation of feature importances, and blind-validation of the test data. The residues within the train data were used for all steps except for the final blind-validation.

In several stages, hyperparameters of the extra-trees predictor were tuned. This was conducted using GridSearchCV by varying the score criteria (gini or entropy), the number of trees (50-200), the number of features tested per node ("automatic" or square-root of total features), the maximum depth of the trees (5-10, or no limit), or the tree size limitation based on the number of features in each leaf (3-7). To improve the accuracy of later measures of feature importance, each feature was only used once in the tree (bootstrap=False).

For the removal of duplicate features, we initially identified all feature pairs with a linear correlation coefficient above 0.6, based on the data from all residues in the train data. We tuned an extra-trees predictor as described above with all features, and calculated the feature importances using the mean decrease in impurity. For each duplicate feature pair, we removed the feature with the least importance for the algorithm.

After initial tuning, feature reduction was conducted to retain the 20 most important features according to the extra-trees algorithm, using the recursive feature elimination function of python sklearn package and average_precision as a scoring method. To aid in later statistical analyses, we also kept the 20 features that most distinguished interface residues according to an ANOVA analysis. Feature reduction for ANOVA was conducted using the SelectKBest function of the sklearn package in python in combination with the "f_classif" ANOVA function as the scoring method. Furthermore, we retained a select number of features analysed in this manuscript in detail, including GxxxG, conservation, DImax, relative_polarity, residue_depth, and branched.

Since there was some overlap between these feature lists, the final THOIPA predictor contained 27 features in total. The final hyperparameters for THOIPA were then obtained by repeating the tuning as described above.

Crossvalidation of the train subset was conducted using a variant of the "leave-one-out" method, where the validation subset comprised the residues of the TMD being tested, and the train subset comprised all other residues of all other TMDs. To remove any possibility that THOIPA was "remembering" the interface residues of homologous TMDs rather than "learning" interface residue properties, we ensured validation of a TMD was never performed with a possible distant homologue in the training set. This was conducted by sorting the TMDs into clusters based on a pairwise amino acid identity of at least 15% between the full protein sequences. Pairwise amino acid identity was calculated by a global alignment using the BioPython pairwise2 align function, with a gap-open penalty -40 and a gap extension penalty of -0.5. During leave-one-out crossvalidation, the TMD of interest and any possible homologues were left out of the train data.

The feature importances of THOIPA were obtained from train data using a mean decrease in impurity and also by measuring the mean decrease in accuracy with two validation methods. The mean decrease in impurity was calculated using the entropy formula, and relates to the proportion of correctly classified samples at nodes containing that feature. To calculate the mean decrease in accuracy, the data for each feature being investigated was shuffled between all samples. A tenfold crossvalidation was performed by splitting the residues of the train subset, and the decrease in prediction performance was calculated using the precision-recall AUC and AUBOC5 validation method described below.

Since there are no other algorithms similar to THOIPA for the prediction of homotypic TMD interfaces, we compared performance with two automated predictors of TM homodimer structures, PREDDIMER [32, 33], and TMDOCK [34]. Each TMD sequence was submitted to the respective PREDDIMER or TMDOCK online server. The top ranked structure according to the respective algorithm was used for validation. PREDDIMER required TMDs to be at least 20 residues. For TMDs shorter than 20 residues, we therefore extended the sequence by one residue at each end (starting at the C-term) until the length reached 20. In contrast, TMDOCK automatically truncated many of the TMDs. Therefore, we validated each TMD separately for all residue positions with both experimental data and a predicted structure. The mean TMD length used for validation was 21.8 residues for THOIPA, 23.0 for PREDDIMER, and 20.8 residues for TMDOCK.

Best overlap (BO) validation

During THOIPA development, we aligned our validation method to the goal of predicting the small number of key residues involved in homotypic TMD interactions. This is especially appropriate for ToxR data, where a few key residues were usually mutation-sensitive, and the remaining residues comprise a noisy background. The validation method required the following properties:

1) to measure precision (true-positive rate) for a small number of residues in each TMD with the highest prediction scores
2) to indicate the number of top residues at which performance is best
3) to give a measure of individual performance for each TMD
4) for overall performance, to apply equal weights to each TMD, rather than to each residue

We therefore developed our own validation method, referred to here as best overlap (BO) validation, which fulfilled all the above requirements. This method somewhat resembles the precision@k methodology that is used in the field of information retrieval to validate document retrieval by internet search engines. The identification of TMD interface residues differs significantly from most information retrieval tasks in that the total number of possible residues is small, and the precision resulting from a random selection can be quite significant. This random precision is strongly dependent on the length of each TMD in the dataset (15 to 29 residues).

BO-validation is based on the overlap of two groups of selected residues from the TMD:

1) the sample of residues corresponding to the top (top 1, top 2, top 3, etc) residues according to experimental data
2) the sample of residues corresponding to the top (top 1, top 2, top 3, etc) residues according to a predictor

We rigorously tested BO-validation using randomly generated predictions. It is illustrated by the following example where we validate THOIPA prediction against experimental ToxR data represented by disruption after mutation (interface score). Dark shading indicates high disruption, or high THOIPA score.

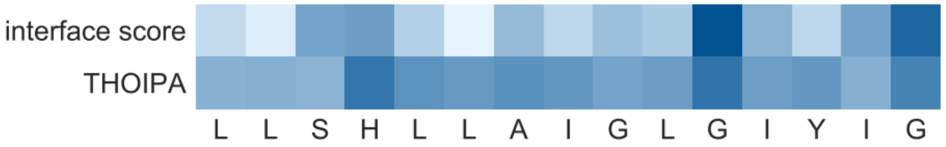

We first rank the experimental and prediction scores, where 1 represents the most important residue for the TMD interaction.

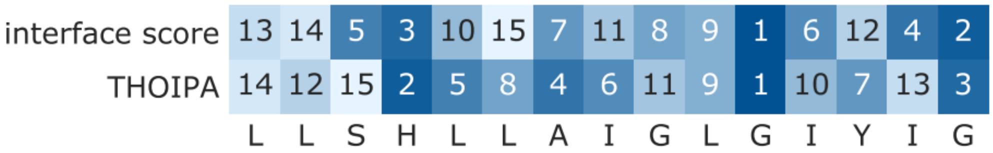

We assess the overlap in residues between the experimental and prediction data for a particular sample size. The sample size represents the number of "top" residues examined according to the experiment or predictor. For example, at sample size 5, we determined how many of the top 5 residues according to experimental data were among the top 5 predicted by THOIPA. We calculated the observed overlap, and the expected overlap by random chance as follows

$$f = \frac{n}{s}, \; r = \frac{s}{l}$$

where f is the observed fraction of overlapping residues, r is the expected random fraction of overlapping residues, n is the observed number of overlapping residues at that sample size, s is the sample size, and l is the length of the TMD. The performance is calculated as follows:

$$p = f - r$$

where p is simply the fraction of correctly predicted overlapping residues (i.e., precision), minus the fraction expected by random chance. This is visually demonstrated below for sample sizes 1 to 4, where the "overlap" row represents whether the experiment and predictor have an overlap at that position:

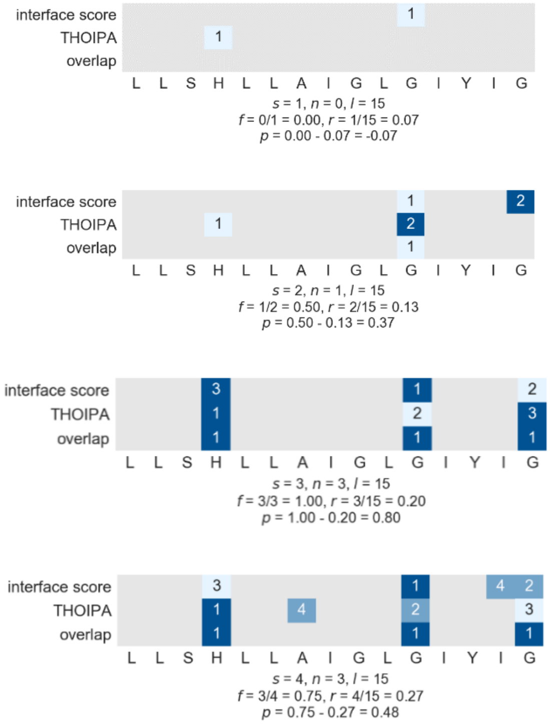

A plot of f, r and p over the entire TMD length of a single protein shows that the random values rise quickly, limiting the possible performance above random.

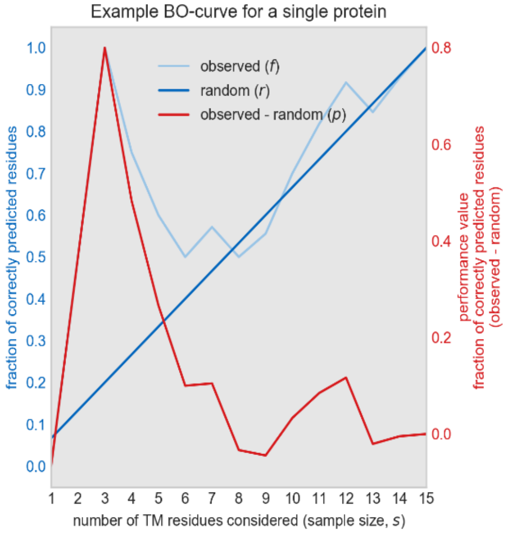

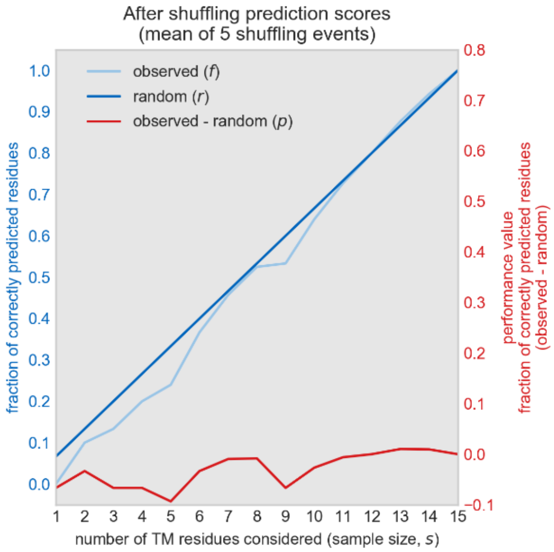

The accuracy of the calculated random scores is confirmed by processing shuffled prediction values (above right). The performance of shuffled predictions clearly conforms to calculated random values. For datasets of multiple proteins, we typically plotted the performance above random, *p* (observed – random), for sample sizes of 1 to 10.

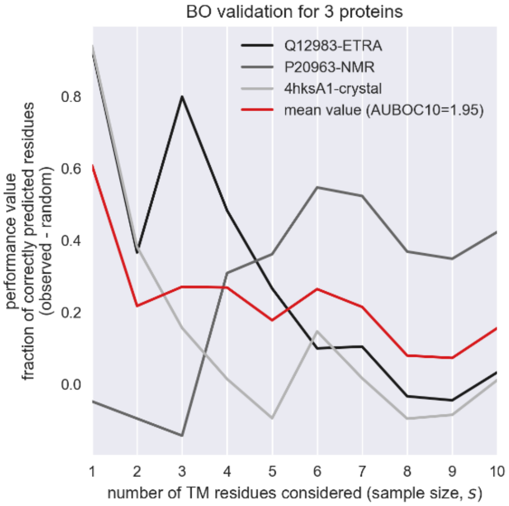

To visualise the overall performance for a dataset of multiple TMDs, we typically calculated the mean performance value for all proteins in the dataset at each sample size. As a performance value for individual TMDs or a dataset, we took the area under the curve of p for the desired sample size (e.g. area under the BO curve from 1 to 5, AUBOC5). This is appropriate considering that there were approximately 6.1 interface residues per TMD in our datasets, and that the end users of automated TMD interface prediction algorithms are primarily interested in the top 1-5 residues according to the predictor.

Note that the maximum AUBOC5 depends on the distribution of TMD-lengths in the dataset. The AUBOC5 measure is therefore a relative value that is appropriate for comparing multiple predictors against a single dataset. The AUBOC values cannot be used to compare performance between different datasets.

Text S2 Supporting discussion

Novel TM homodimer interfaces identified in this study.

The receptor tyrosine kinases DDR1 and DDR2 were the only homologues in the ETRA dataset, showing significant (71%) amino acid identity in the TM region. Previous studies have established that DDR TMD-TMD interaction does not depend on the N-terminal GxxxA motif and it was hypothesised to depend on a leucine zipper [35, 36]. Our data supports this, showing that the self-interaction both DDR1 and DDR2 TMDs indeed relies on long, Leu/Ile dominated interfaces, being IxxxxLxxIIxxLxxIIxxML and LIxxLxxxIxxLxxIIxxIL respectively. The interfaces are therefore similar but not identical.

For ATP1B1 (Na/K-ATPase β-subunit) we identify an interface composed of diverse residue types, comprising GxxxGxFxxxxQ. Of these, G17 was by far the most mutation-sensitive residue. This reveals the remarkable prescience of Barwe et al. [37], who suggested this in a previous study based on limited experimental data. An unusual feature of ATP1B1 is the presence of three mutations (Y4A, Y8L, and V21A) that increased the dimerisation to ~150% of the wildtype. The two tyrosines (Y4, Y8) lie on the same helix face, whereas V21 lies on the side of the helix containing the GxxxG motif. Mutations to Y8 were unique in that they could significantly increase (Y8L, Y8V) or decrease (Y8A) the ToxR signal. This complex response may indicate a secondary homodimer interface.

The expression level of siglec7 is strongly related to liver injury which makes it a potential mortality prediction biomarker [38]. In a recent study of the largest clusters of bitopic proteins within the human genome [39], siglec7 showed the highest ToxR activity of all 33 representative TMDs tested. Although some siglec proteins have been proposed to exist as disulfide-linked homodimers [40], little is known about the non-covalent self-interaction of their TMDs. The TMD of siglec7 is rich in small residues, with 10/20 residues being G, A, S or C. Mutagenesis revealed a clear interface of GxxxGAxxxAxxFxS.

The Armcx6 proteins are a recently evolved protein family within eutherian mammals. They are expressed in the nervous system, and have been proposed roles, such as in mitochondrial trafficking in neurons [41]. The TMD of Armcx6 has previously been shown to dimerise strongly in the ToxR system [39]. The TMD is relatively polar, containing two charged residues and ten small residues, several of which are highly conserved. Of these, we confirm the key role of the central GxxxG motif, however the expanded interface of RxxxxMxxGLxIG involves diverse residue types and has a strong α-helical periodicity.

The ToxR data of this study indicates that the Ire1 TMD has medium strength self-interaction, based around a key conserved Trp residue. Ire1 is an important signalling protein that clusters in the ER membrane under ER stress conditions [42, 43]. It contains an N-terminal lumenal domain that detects unfolded proteins, a cytoplasmic kinase domain, and also a cytoplasmic mRNA endonuclease domain. Unfolded proteins in the lumen are thought to increase dimerisation or oligomerisation of Ire1 [44]. This leads to autophosphorylation of the kinase domain in the cytoplasm, and subsequent activation of the endonuclease domain [44], which then splices the mRNA of XBP1 [45]. This signalling cascade is central to the unfolded protein response, an important process in age-related diseases. XBP1 overexpression is known to reduce the expression levels of APP [46], the precursor to the toxic Aβ42 fragment implicated in Alzheimer's disease. Deletion of the Ire1 endonuclease domain dramatically reduces Alzheimer-related symptoms in mice [47]. From the ToxR data, the motif WVxxxxT was found to be important for the interface. The importance of the central Trp residue was confirmed with mutations to three different residues. This independently confirms the results of a recent, detailed study of the Ire1 TMD, which suggests that the central Trp residue (W457) is involved in palmitate-induced oligomerisation [48].

Tie1 is a member of the endothelial-specific receptor tyrosine kinase family which is important for cell proliferation, migration and survival during angiogenesis. Genetic studies in mice have

demonstrated that Tie1 is required for normal embryonic vascular development. Tie1 associated with Tie2 performs complementary functions which are mediated by a broad surface area within its basic region [49]. Targeted disruption of the Tie1 gene results in a lethal phenotype with severe disruption to the normal integrity of the vasculature [50]. No ligand has yet been identified for Tie1. The defined interface LxVxxxxxxxxLxxL does not fit α-helical periodicity. It may be consistent with a kinked structure of the helix which would allow for close proximity of both partial motifs in the dimer or alternate states of a dimer containing different interfaces.

Signalling by receptor protein-tyrosine phosphatases (RPTPs) has been proposed to depend on TMD dimerisation [16]. In this study, we investigated the interfaces of three human RPTPs that showed strong self-interaction in the TOXCAT assay according to a previous study [16]. PTPRU shows a clear, defined interface of LxxGxxxxxxAxxxxxL. PTPRG interacts via an interface strongly dependent on TxxxLxxxxxxL, but is clearly supported by other residues on the same helix face, whose disruption values were below the 0.24 threshold. The residues of this entire helix face are exceptionally well conserved. For PTPRO, single-amino acid mutations only weakly disrupted the homodimer and disruptive mutations were found at both ends of the TMD, giving an apparent long interface of VIxxLxxxxxxxxxxLxxxxIxL. An α-helical periodicity was seen in the disruption pattern of the N-terminal residues (VVIxxLAxLS), but not the C-terminal residues (LxxxxIxL). The TMD interfaces from the three RPTPs identified in this study (PTPRU, PTPRG, and PTPRO) were therefore all distinct from each other, and from the GxxxGxxG-based interface previously proposed for DEP1 [16]. This diversity is consistent with the large evolutionary distance separating these proteins (amino acid identity below 40%).

Supporting Figures

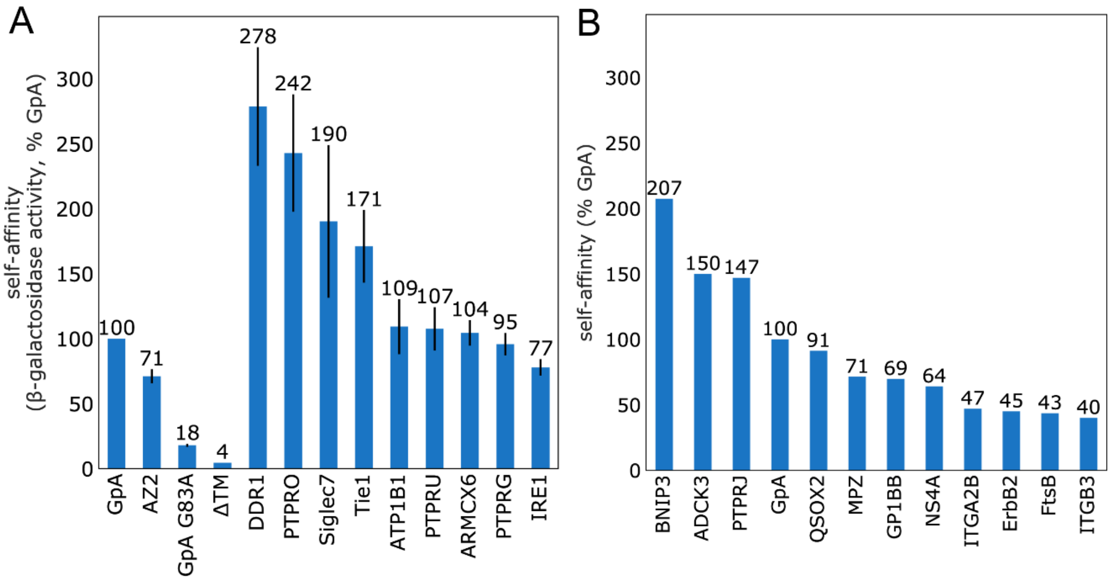

Fig S1. The ETRA dataset is primarily comprised of TMDs with strong self-affinity. (A) ETRA TMDs with scanning mutagenesis data from this study. The references glycophorin A (GpA), AZ2, GpA G83A and ΔTM represent strong, moderate, weak and no homodimers respectively. Means ± SEM of ≥3 replicates. (B) ETRA TMDs with scanning mutagenesis data from literature. Note that the dsTβL method used for ErbB2 mutagenesis does not provide a value for relative affinity [7]. For ErbB2 the dimer affinity was therefore derived from an earlier TOXCAT study [51]. NS4A GALLEX data was normalised as described in the Text S1. Supporting methods.

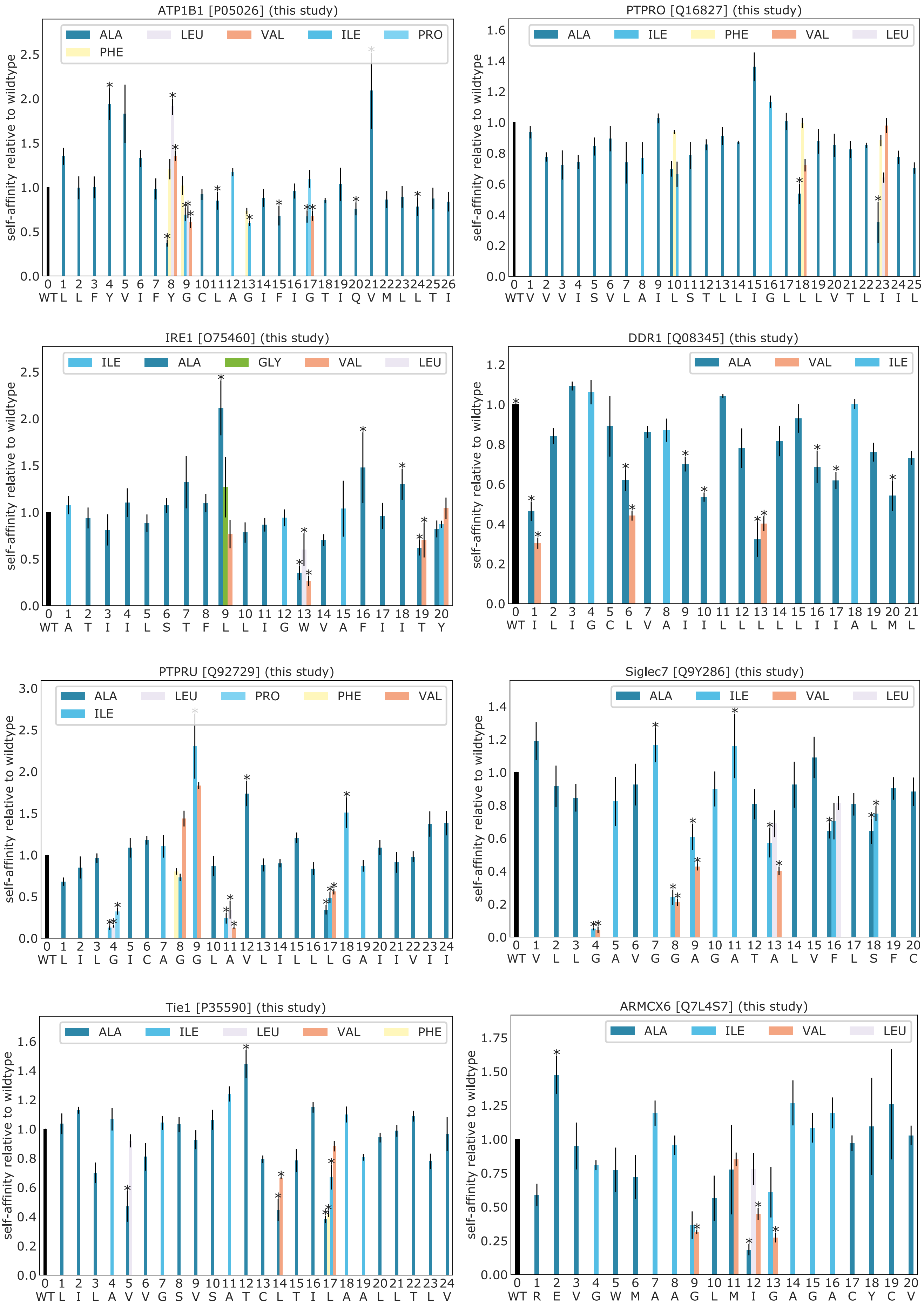
ATP1B1 [P05026] (this study)
ALA LEU VAL ILE PRO PHE
self-affinity relative to wildtype
WT L L F Y V I F Y G C L A G I F I G T I Q V M L L T I
PTPRO [Q16827] (this study)
ALA ILE PHE VAL LEU
WT V V V I S V L A I L S T L L I G L L L V T L I I L
IRE1 [O75460] (this study)
ILE ALA GLY VAL LEU
WT A T I I L S T F L L I G W V A F I I T Y
DDR1 [Q08345] (this study)
ALA VAL ILE
WT I L I G C L V A I I L L L L L I I A L M L
PTPRU [Q92729] (this study)
ALA LEU PRO PHE VAL ILE
WT L I L G I C A G G L A V L I L L L G A I I V I I
Siglec7 [Q9Y286] (this study)
ALA ILE VAL LEU
WT V L L G A V G G A G A T A L V F L S F C
Tie1 [P35590] (this study)
ALA ILE LEU VAL PHE
WT L I L A V V G S V S A T C L T I L A A L L T L V
ARMCX6 [Q7L4S7] (this study)
ALA ILE VAL LEU
WT R E V G W M A A G L M I G A G A C Y C V

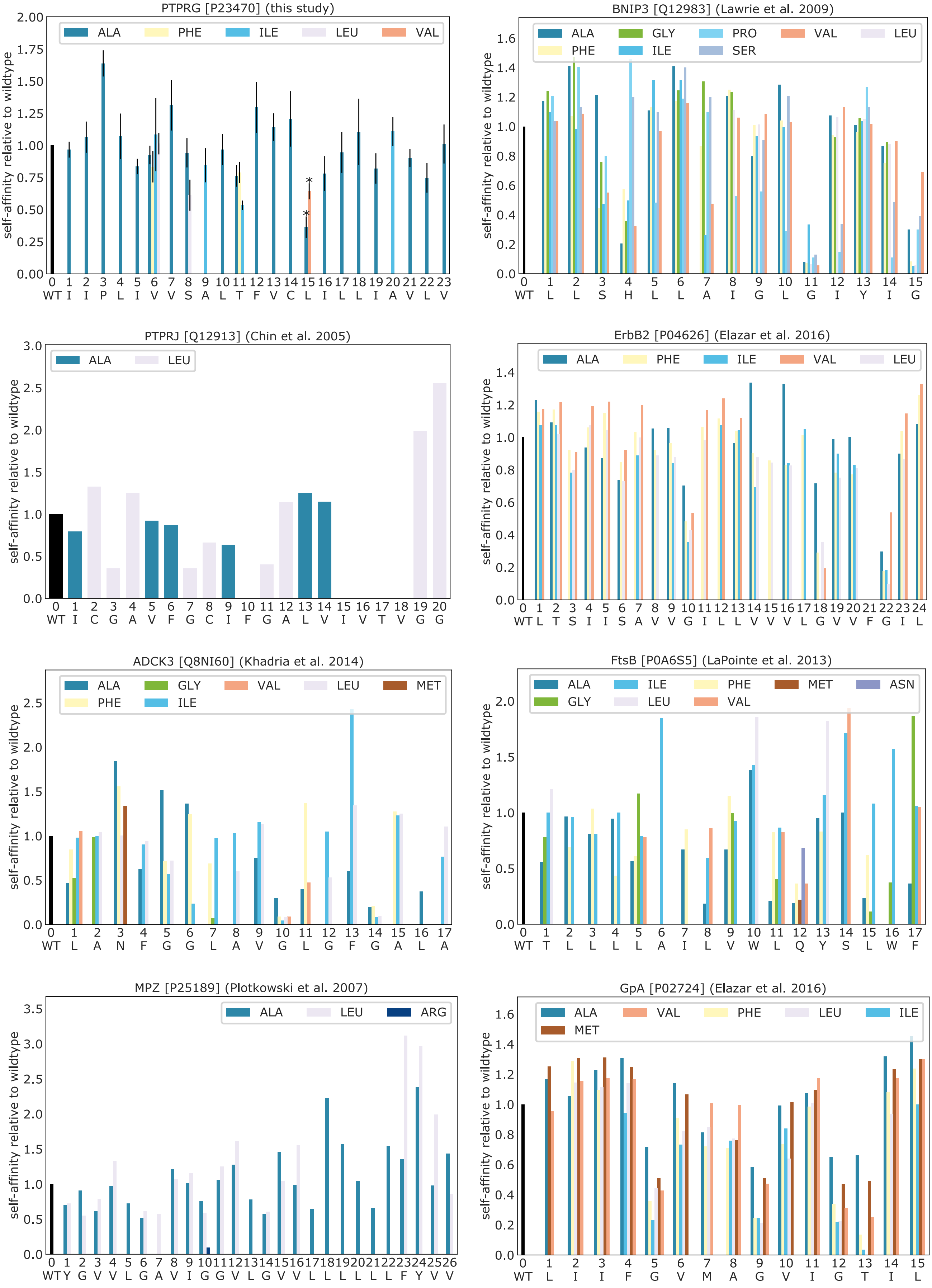

PTPRG [P23470] (this study)
ALA PHE ILE LEU VAL
self-affinity relative to wildtype
0.00 0.25 0.50 0.75 1.00 1.25 1.50 1.75 2.00
0 1 2 3 4 5 6 7 8 9 10 11 12 13 14 15 16 17 18 19 20 21 22 23
WT I I P L I V V S A L T F V C L I L L I A V L V
*
*
BNIP3 [Q12983] (Lawrie et al. 2009)
ALA GLY PRO VAL LEU PHE ILE SER
self-affinity relative to wildtype
0.0 0.2 0.4 0.6 0.8 1.0 1.2 1.4 1.6
0 1 2 3 4 5 6 7 8 9 10 11 12 13 14 15
WT L L S H L L A I G L G I Y I G
PTPRJ [Q12913] (Chin et al. 2005)
ALA LEU
self-affinity relative to wildtype
0.0 0.5 1.0 1.5 2.0 2.5 3.0
0 1 2 3 4 5 6 7 8 9 10 11 12 13 14 15 16 17 18 19 20
WT I C G A V F G C I F G A L V I V T V G G
ErbB2 [P04626] (Elazar et al. 2016)
ALA PHE ILE VAL LEU
self-affinity relative to wildtype
0.0 0.2 0.4 0.6 0.8 1.0 1.2 1.4
0 1 2 3 4 5 6 7 8 9 10 11 12 13 14 15 16 17 18 19 20 21 22 23 24
WT L T S I I S A V V G I L L V V V L G V V F G I L
ADCK3 [Q8NI60] (Khadria et al. 2014)
ALA GLY VAL LEU MET PHE ILE
self-affinity relative to wildtype
0.0 0.5 1.0 1.5 2.0 2.5
0 1 2 3 4 5 6 7 8 9 10 11 12 13 14 15 16 17
WT L A N F G G L A V G L G F G A L A
FtsB [P0A6S5] (LaPointe et al. 2013)
ALA ILE PHE MET ASN GLY LEU VAL
self-affinity relative to wildtype
0.0 0.5 1.0 1.5 2.0
0 1 2 3 4 5 6 7 8 9 10 11 12 13 14 15 16 17
WT T L L L L A I L V W L Q Y S L W F
MPZ [P25189] (Plotkowski et al. 2007)
ALA LEU ARG
self-affinity relative to wildtype
0.0 0.5 1.0 1.5 2.0 2.5 3.0 3.5
0 1 2 3 4 5 6 7 8 9 10 11 12 13 14 15 16 17 18 19 20 21 22 23 24 25 26
WT Y G V V L G A V I G G V L G V V L L L L L L F Y V V
GpA [P02724] (Elazar et al. 2016)
ALA VAL PHE LEU ILE MET
self-affinity relative to wildtype
0.0 0.2 0.4 0.6 0.8 1.0 1.2 1.4 1.6
0 1 2 3 4 5 6 7 8 9 10 11 12 13 14 15
WT L I I F G V M A G V I G T I L

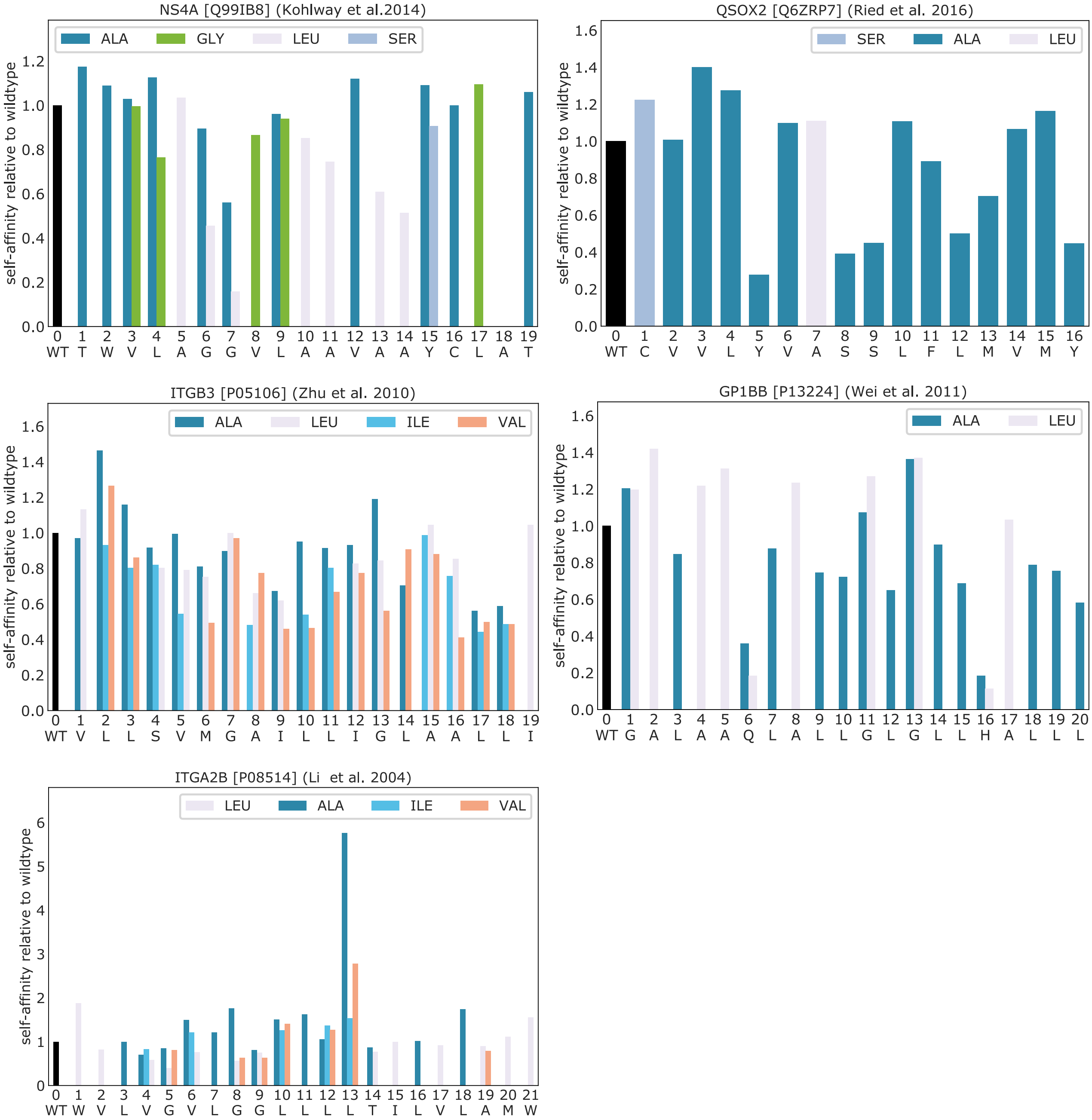

NS4A [Q99IB8] (Kohlway et al.2014)
ALA GLY LEU SER
self-affinity relative to wildtype
0.0 0.2 0.4 0.6 0.8 1.0 1.2
0 1 2 3 4 5 6 7 8 9 10 11 12 13 14 15 16 17 18 19
WT T W V L A G G V L A A V A A Y C L A T
QSOX2 [Q6ZRP7] (Ried et al. 2016)
SER ALA LEU
self-affinity relative to wildtype
0.0 0.2 0.4 0.6 0.8 1.0 1.2 1.4 1.6
0 1 2 3 4 5 6 7 8 9 10 11 12 13 14 15 16
WT C V V L Y V A S S L F L M V M Y
ITGB3 [P05106] (Zhu et al. 2010)
ALA LEU ILE VAL
self-affinity relative to wildtype
0.0 0.2 0.4 0.6 0.8 1.0 1.2 1.4 1.6
0 1 2 3 4 5 6 7 8 9 10 11 12 13 14 15 16 17 18 19
WT V L L S V M G A I L L I G L A A L L I
GP1BB [P13224] (Wei et al. 2011)
ALA LEU
self-affinity relative to wildtype
0.0 0.2 0.4 0.6 0.8 1.0 1.2 1.4 1.6
0 1 2 3 4 5 6 7 8 9 10 11 12 13 14 15 16 17 18 19 20
WT G A L A A Q L A L L G L G L L H A L L L
ITGA2B [P08514] (Li et al. 2004)
LEU ALA ILE VAL
self-affinity relative to wildtype
0 1 2 3 4 5 6
0 1 2 3 4 5 6 7 8 9 10 11 12 13 14 15 16 17 18 19 20 21
WT W V L V G V L G G L L L L T I L V L A M W

Fig S2. Scanning mutagenesis data for ETRA TMDs. Data is normalised to the wildtype sequence of a given TMD. For the ToxR data obtained during this study, data represents mean ± SEM of ≥3 replicates. The dsTβL data (GpA, ErbB2), GALLEX data (NS4A), and ITGB3 data were normalised as described in the *Text S1. Supporting methods*. All data is available in the OSF data repository (https://osf.io/txjev).

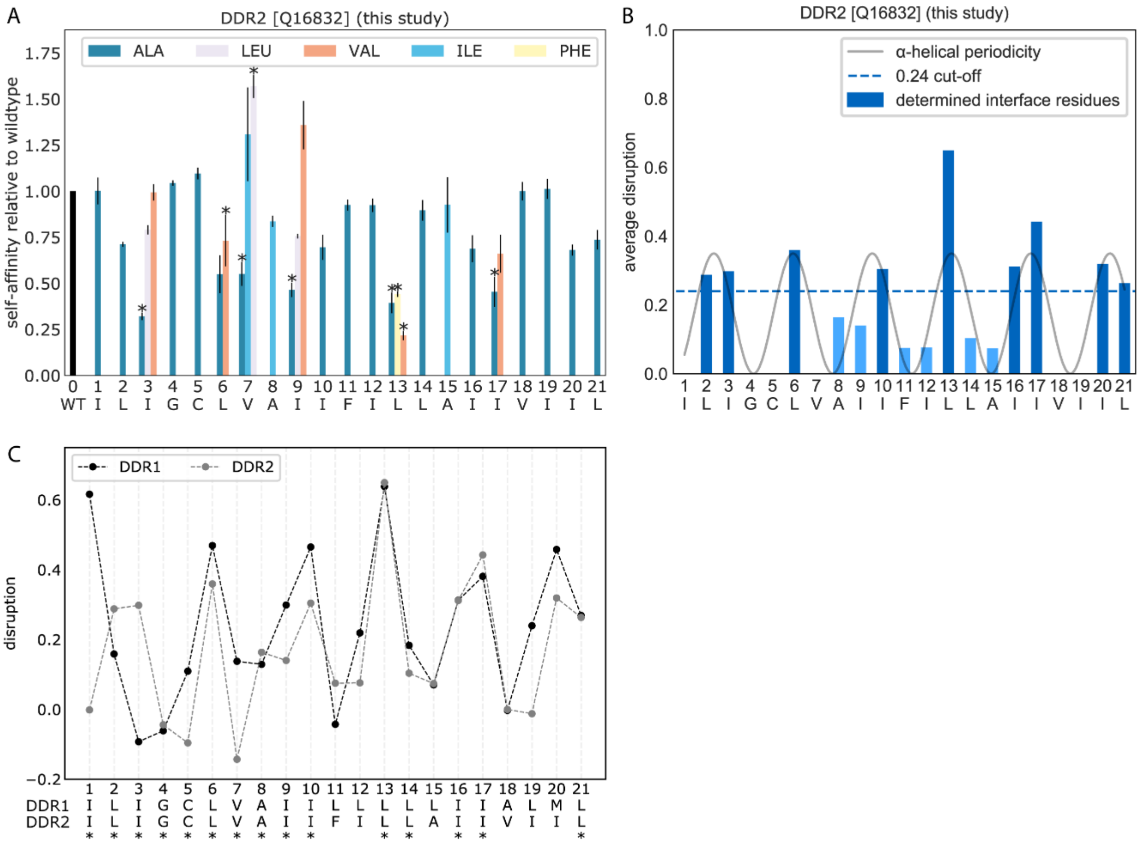

Fig S3. The DDR2 homotypic TMD interface is similar to that of its homologue, DDR1, as shown by scanning mutagenesis. Note that DDR2 was not included in the ETRA dataset, as it was redundant with DDR1 due to 71% amino acid identity in the TMD region. (A) Scanning mutagenesis data for DDR2. (B) DDR2 average disruption and identified interface residues. The interface is defined as described for the TMDs of the ETRA dataset. (C) Line chart showing mean disruption after mutation for DDR2, in comparison to DDR1. Stars on the x-axis indicate identical residues of DDR1 and DDR2 TMD sequences in an amino acid alignment.

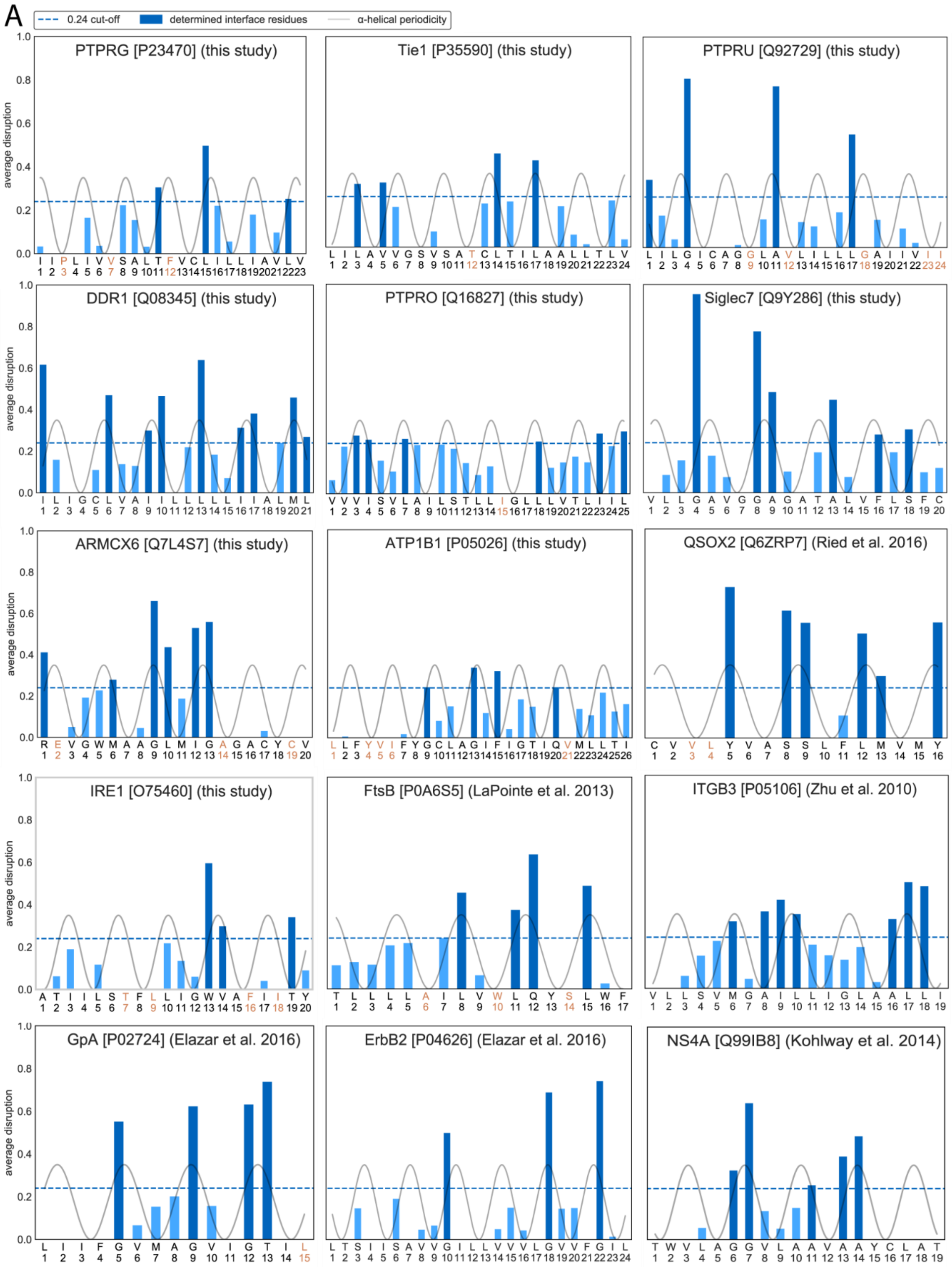
A
0.24 cut-off
determined interface residues
α-helical periodicity
average disruption
PTPRG [P23470] (this study)
Tie1 [P35590] (this study)
PTPRU [Q92729] (this study)
DDR1 [Q08345] (this study)
PTPRO [Q16827] (this study)
Siglec7 [Q9Y286] (this study)
ARMCX6 [Q7L4S7] (this study)
ATP1B1 [P05026] (this study)
QSOX2 [Q6ZRP7] (Ried et al. 2016)
IRE1 [O75460] (this study)
FtsB [P0A6S5] (LaPointe et al. 2013)
ITGB3 [P05106] (Zhu et al. 2010)
GpA [P02724] (Elazar et al. 2016)
ErbB2 [P04626] (Elazar et al. 2016)
NS4A [Q99IB8] (Kohlway et al. 2014)

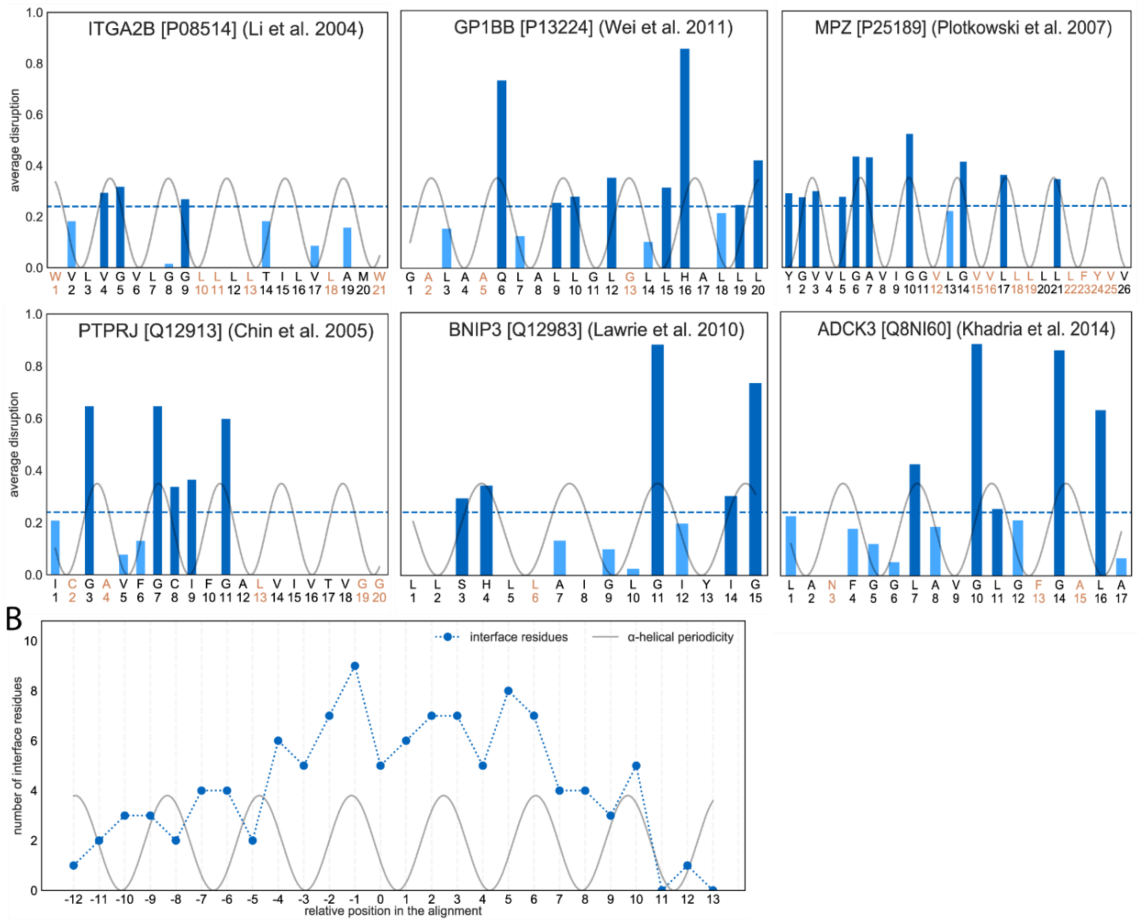

Fig S4. Interface residues determined for ETRA TMDs. Average disruption after mutation was calculated for each residue position, based on the data in Fig S2. Positions with a disruption >0.24 (i.e. >24% decrease in dimer signal relative to the wildtype, dotted horizontal line) were classified as interface residues (dark blue bars). To indicate an α-helical pattern, we fitted the mean values to a sine curve with a periodicity of 3.6 (grey). (A) Barcharts showing average disruption at each position. To improve readability, bars are only shown for positions with positive disruption (i.e. where mutation decreased self-affinity). Positions with strongly negative disruption (< -0.24) are indicated by orange residue labels on the x-axis. (B) Distribution of interface residues as seen after aligning the sequences based on their most central amino acid and summing up the numbers of interface residues at each position. For TMDs whose length was an even number, the N-terminal residue to the left of centre was considered the central one. A sine wave with α-helical periodicity (3.6 residues per turn) was fit to the data.

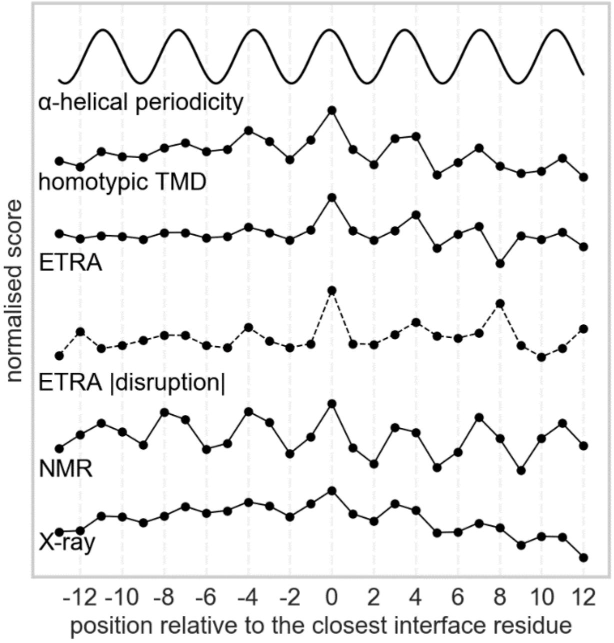

Fig S5. Interface residues show α-helical periodicity. The TMD sequences of each dataset were aligned centrally according to the most important residue for the interaction. The most important residue was the residue with the highest disruption (ETRA) or lowest heavy-atom distance (NMR, X-ray). The disruption and heavy-atom distances were converted to a method-independent "interface score" as described in the *Text S1. Supporting methods*. After alignment of the TMDs, the mean values of the interface score were calculated for each position in the alignment. (A) Full homotypic TMD dataset, (B) ETRA dataset, (C) NMR dataset (D), X-ray dataset. Shown are the data for 12 residues upstream and downstream of the residue with the highest apparent importance.

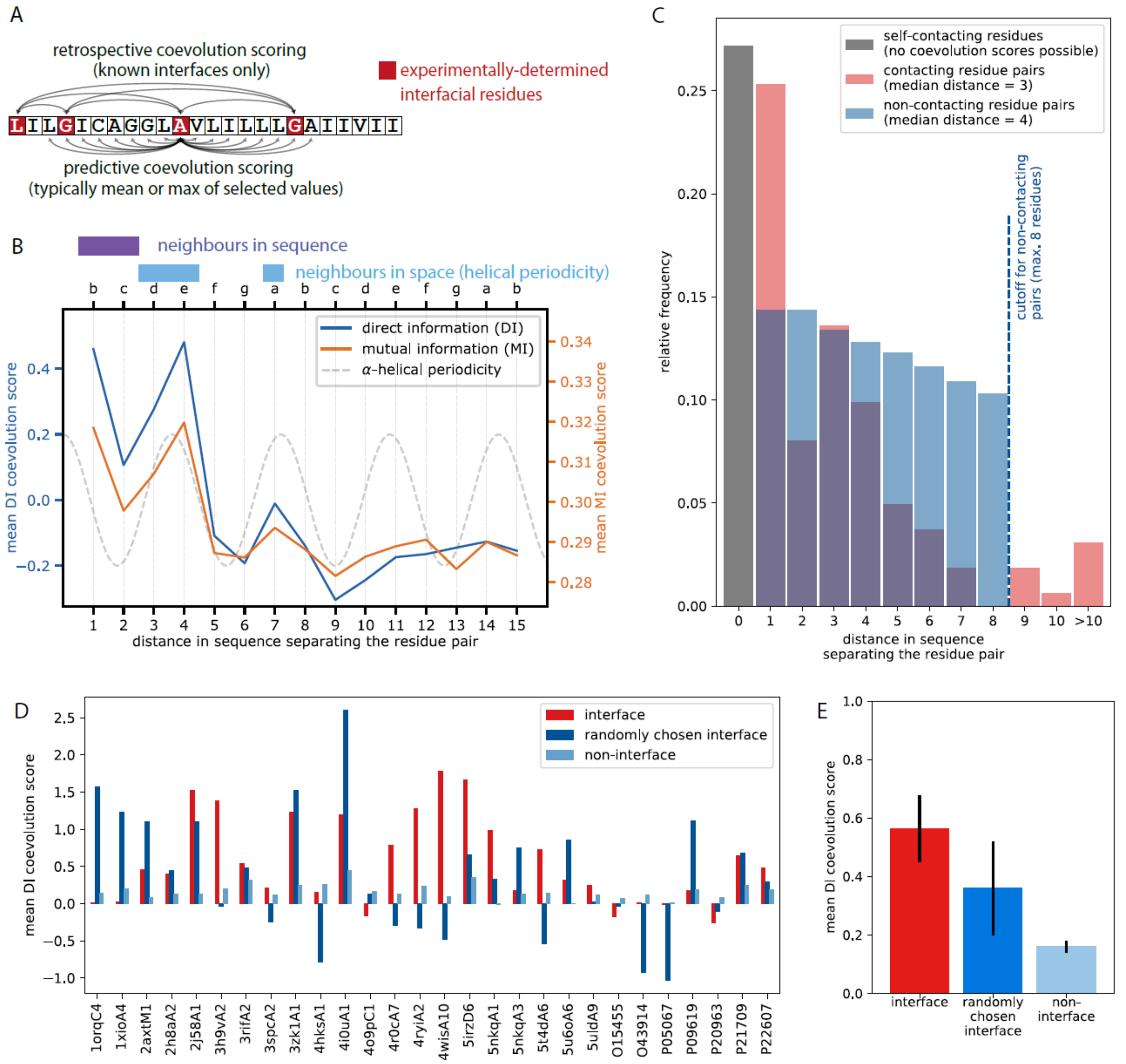

Fig S6. Coevolution of contacting (interface) residues in the NMR and X-ray datasets is biased by the "neighbour effect." (A) Schematic illustration comparing the calculation of retrospective and predictive coevolution values. Retrospective methods require a known interface and thus can compare overall coevolution values between interface and non-interface residues. Predictive methods calculate the mean or maximum of pairwise values, involving any residue of interest [52]. A previous retrospective study [53] had proposed that in TM homodimers, coevolution is higher for interface than non-interface residues. This conclusion was drawn from an analysis comparing mean pairwise DI of contacting residue pairs to the mean pairwise DI of non-contacting residue pairs. In the previous study, non-contacting residues separated by up to 8 residues in sequence were considered. When we applied this method to our own NMR and X-ray dimers, we noticed a few effects and biases that compromised the retrospective evolution approach. (B) The neighbour effect. Coevolution scores are intrinsically higher for residues being close in the protein sequence (i.e., at distances of 1 or 2), or close in space at α-helical periodicity. The relationship between the separation distance and coevolution scores was calculated using the MI and DI scores from the homotypic TMD dataset. The helical pattern followed by both scores shows good correspondence to that previously described for soluble α-helices [54]. Residues are aligned with the classical heptad motif (*abcdefg*), where *a* is the reference residue. Neighbours in sequence of the residue of interest, *a*, occupy positions *b* (direct neighbourhood) and c (separated by one residue). Direct

neighbours of *a* in space comprise positions *d* and *e*, assuming perfect α-helicity. (C) There is a clear difference in the distribution of the distances separating contacting (interface) or non-contacting (non-interface) residue pairs. We noticed that the number of contacting residues was much smaller than the number of non-contacting residues. Thus, the distance in the sequence between contacting residues (median=2) was half the distance between non-contacting residues (median=4). Since the coevolution values are dependent on residue distance (part B) shorter distances between interface residues artificially raise coevolution values of interface residues. Interface residues generally appear more co-evolved, even for dimer configurations that are not found in the organism of interest. D) Mean DI coevolution values for all TMDs in the NMR and X-ray datasets. The mean DI was calculated between all contacting (=interface) and non-contacting (=non-interface) residue pairs as previously described [53]. For each TMD, we also calculated the score for a randomly chosen interface after substituting its contacting amino acids by contacting residue positions of an unrelated TMD. E) Mean values of the data shown in part D. Comparing original and randomly chosen interfaces shows that a proportion of the higher coevolution scores in the original interfaces is due to the fact that average distances between contacting residue pairs are smaller than those of non-contacting ones, i.e., the neighbour effect. Retrospective analyses comparing coevolution of interface and non-interface residues (compare red and light blue bars) are therefore biased. The comparison of real and randomly chosen interfaces leads to a reduction of this bias. However, with only 29 NMR and X-ray TMDs available, it is not certain if such randomisation can completely remove the bias inherent in retrospective analyses. In comparison, analyses based on predictive coevolution measures (Fig 2, Fig S9, Table S1) offer a far more rigorous comparison of residue properties between interface and non-interface residues.

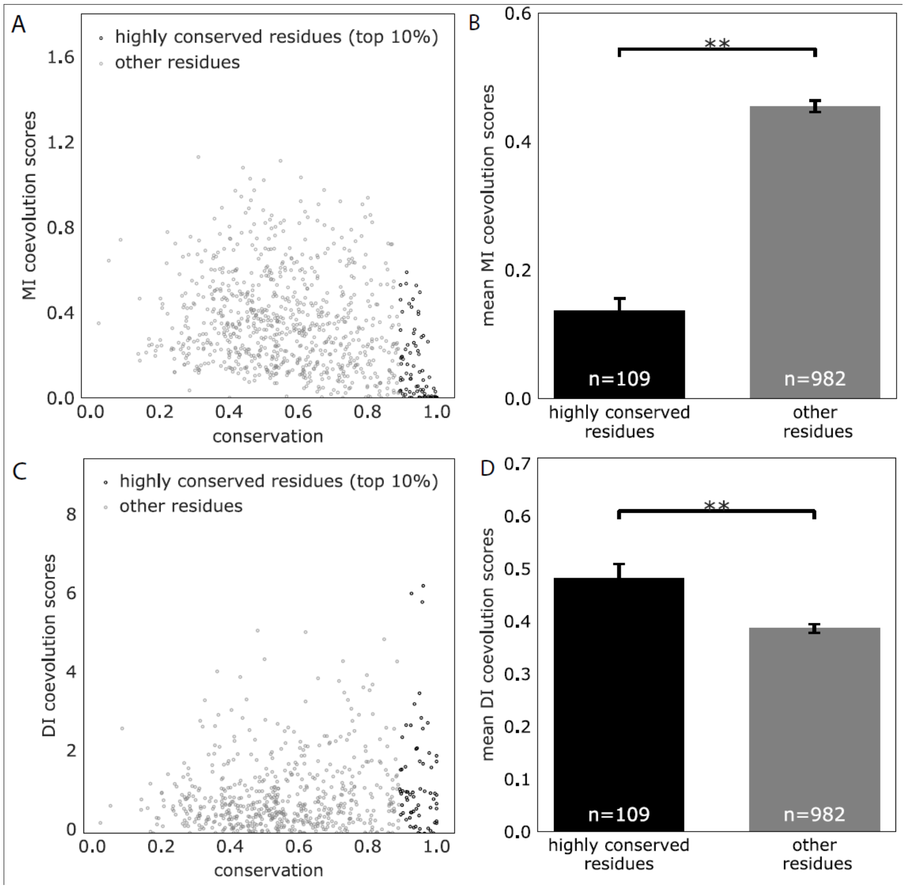

Fig S7. Highly conserved residues are associated with low MI and high DI coevolution scores. For each residue in the homotypic TMD dataset, conservation was plotted against coevolution, represented by MI4mean and DI4mean (see *Text S1. Supporting methods*), before normalisation of values within each TMD. (A) Scatterplot of MI values against conservation. Highly conserved residues (top 10%) are shown in a darker colour. (B) Barchart comparing the most highly conserved residues with all other residues, in respect to MI4mean values. (C) Scatterplot of DI values against conservation. (D) Barchart comparing the most highly conserved residues with all other residues, in respect to DI4mean values. Means ± SEM. Statistical significance was tested using a bootstrapped t-test.

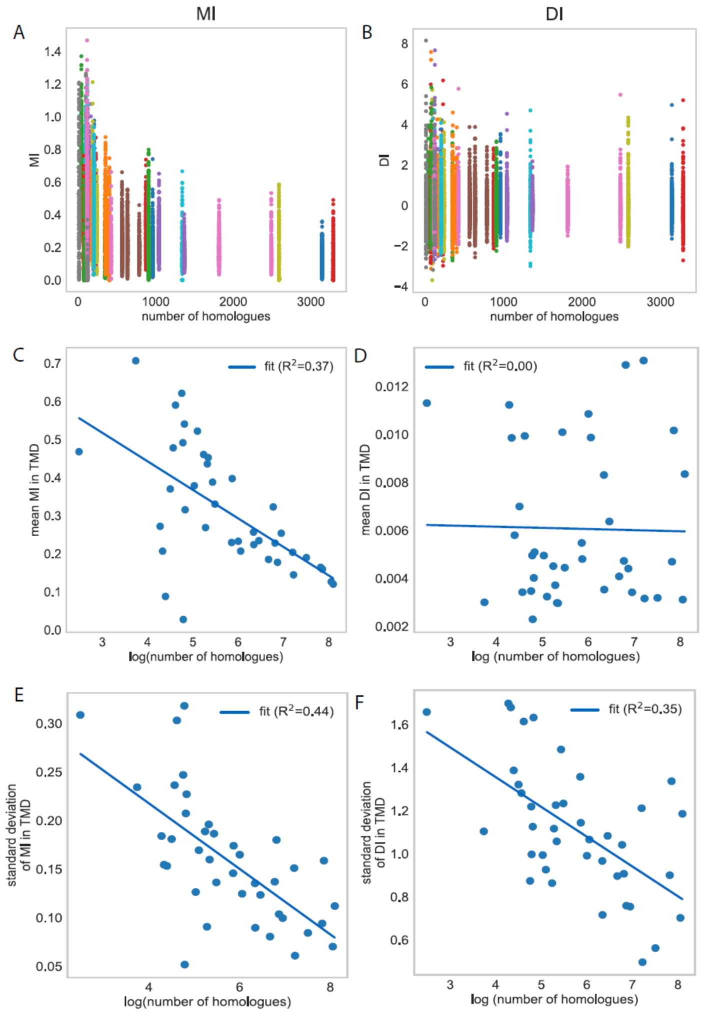

Fig S8 Relationship between coevolution values and the number of homologues. This figure shows the value of normalising MI and DI values within each TMD for statistical and machine-learning approaches. (A, B) The number of homologues is unevenly distributed between the TMDs and has a stronger effect on MI than DI values. MI and DI values at each residue position are plotted against the number of homologues. The number of homologues is a discrete value shared by all residues in a TMD. (C, D) The mean MI values within each TMD are affected by the number of homologues. (E, F) The standard deviation of MI and DI values within each TMD is negatively correlated with the number of homologues.

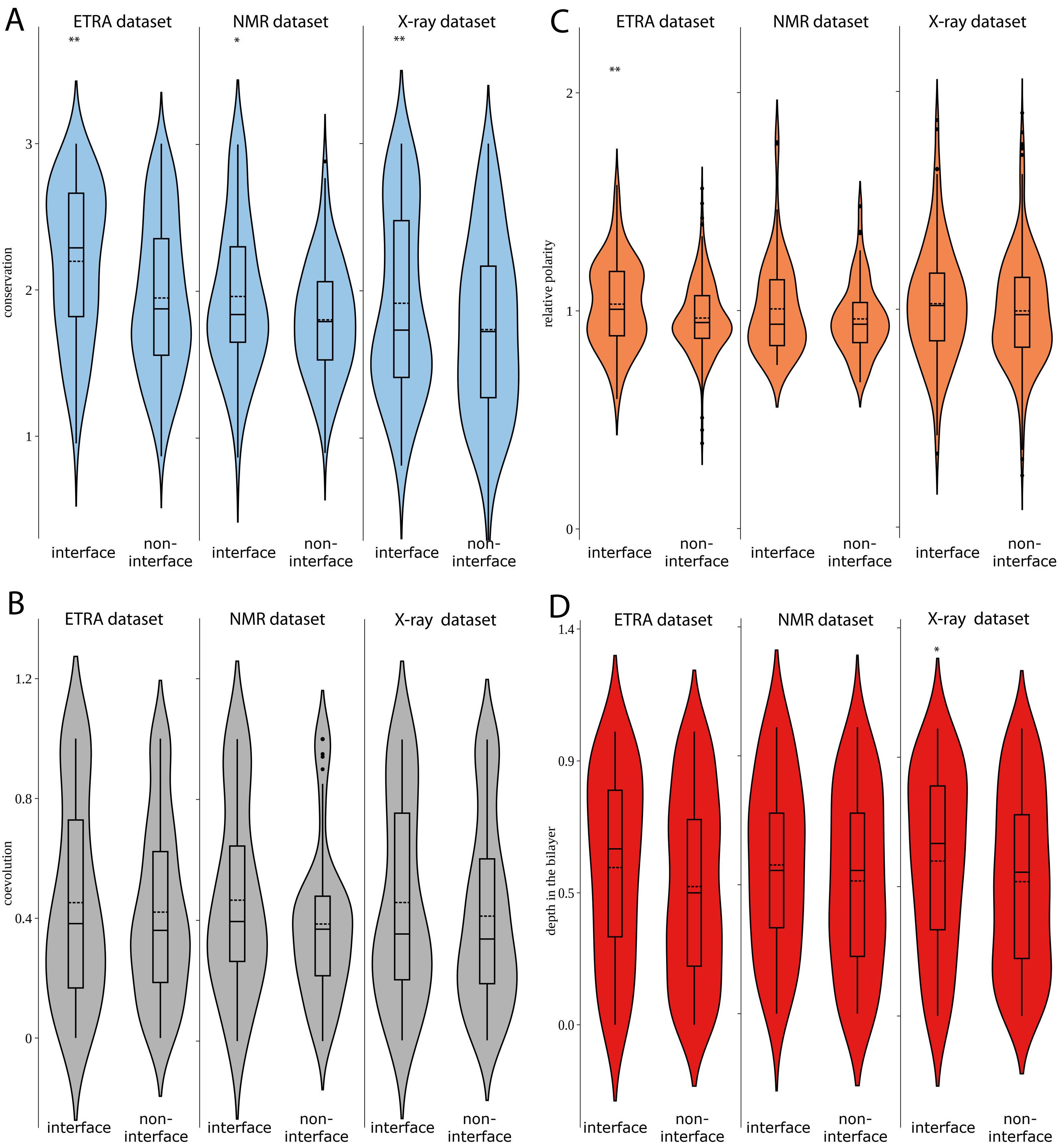

A
ETRA dataset
NMR dataset
X-ray dataset
**
*
**
conservation
3
2
1
interface
non-interface
C
ETRA dataset
NMR dataset
X-ray dataset
**
relative polarity
2
1
0
interface
non-interface
B
ETRA dataset
NMR dataset
X-ray dataset
coevolution
1.2
0.8
0.4
0
interface
non-interface
D
ETRA dataset
NMR dataset
X-ray dataset
*
depth in the bilayer
1.4
0.9
0.5
0.0
interface
non-interface

Fig S9. Analysis of residue features associated with interface residues for each dataset separately.

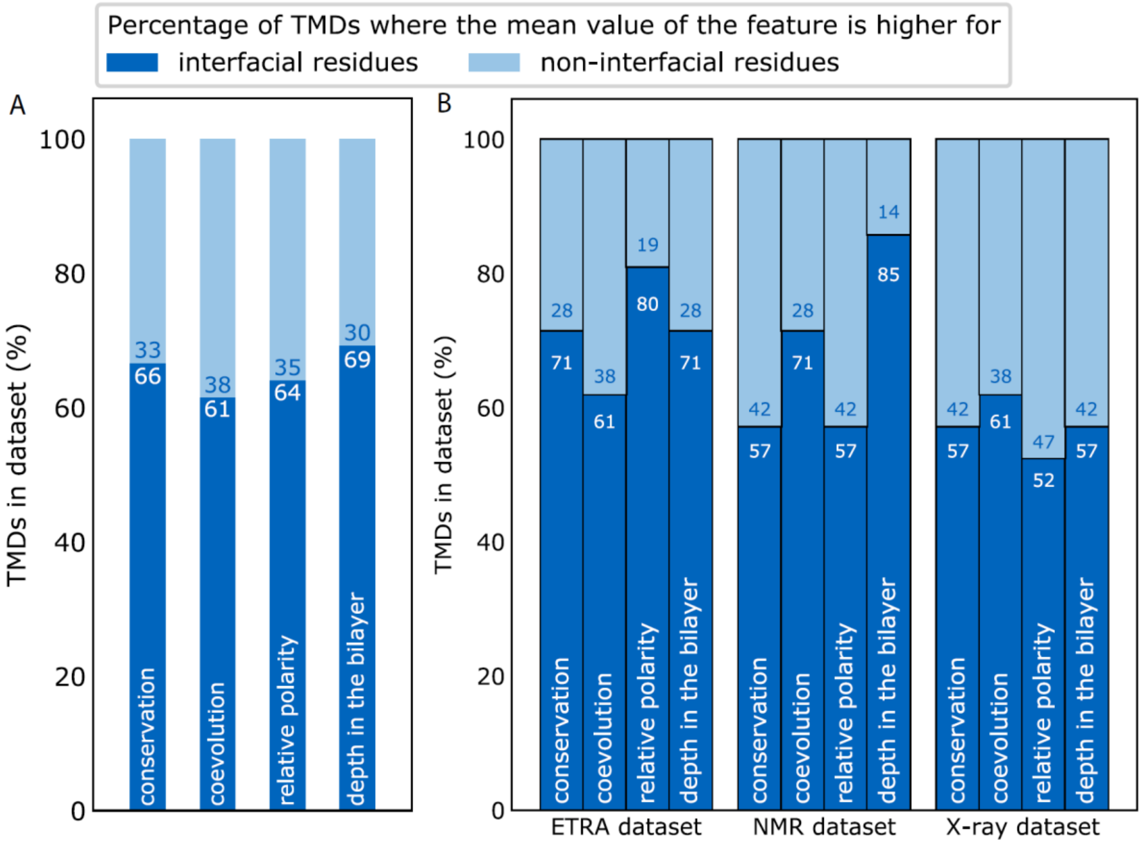

Fig S10. Individual TMDs have unique structural requirements, leading to high variability in residue properties of interfaces. (A) Percentage of TMDs of the homotypic TMD dataset (ETRA, NMR and X-ray) where the mean value of a given residue property is higher for interface or non-interface residues, respectively. (B) Values as in part A but calculated for each dataset separately.

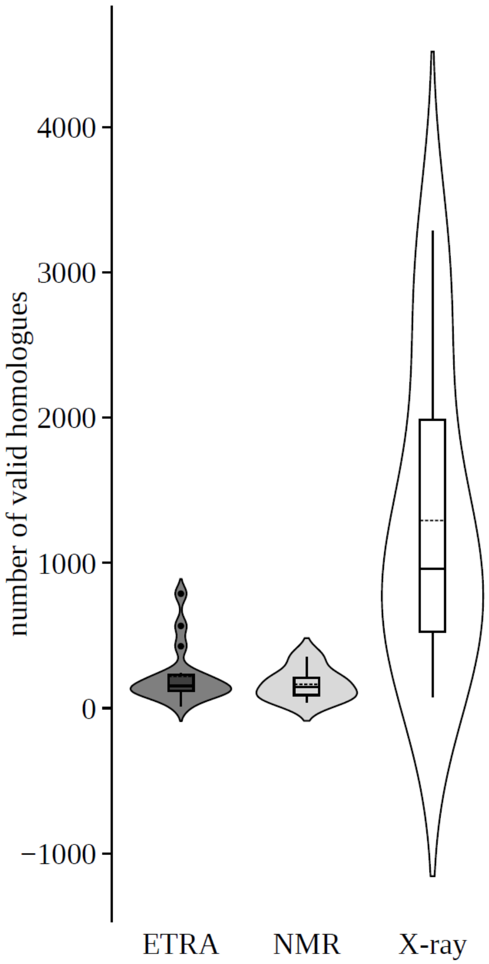

Fig S11. Numbers of valid homologues for TMDs of each dataset. The means are 272, 195 and 1334 for the ETRA, NMR and X-ray datasets, respectively. Filtering and redundancy reduction of homologues was conducted as described in the Methods. Violin plots were constructed as described in **Fig 2**.

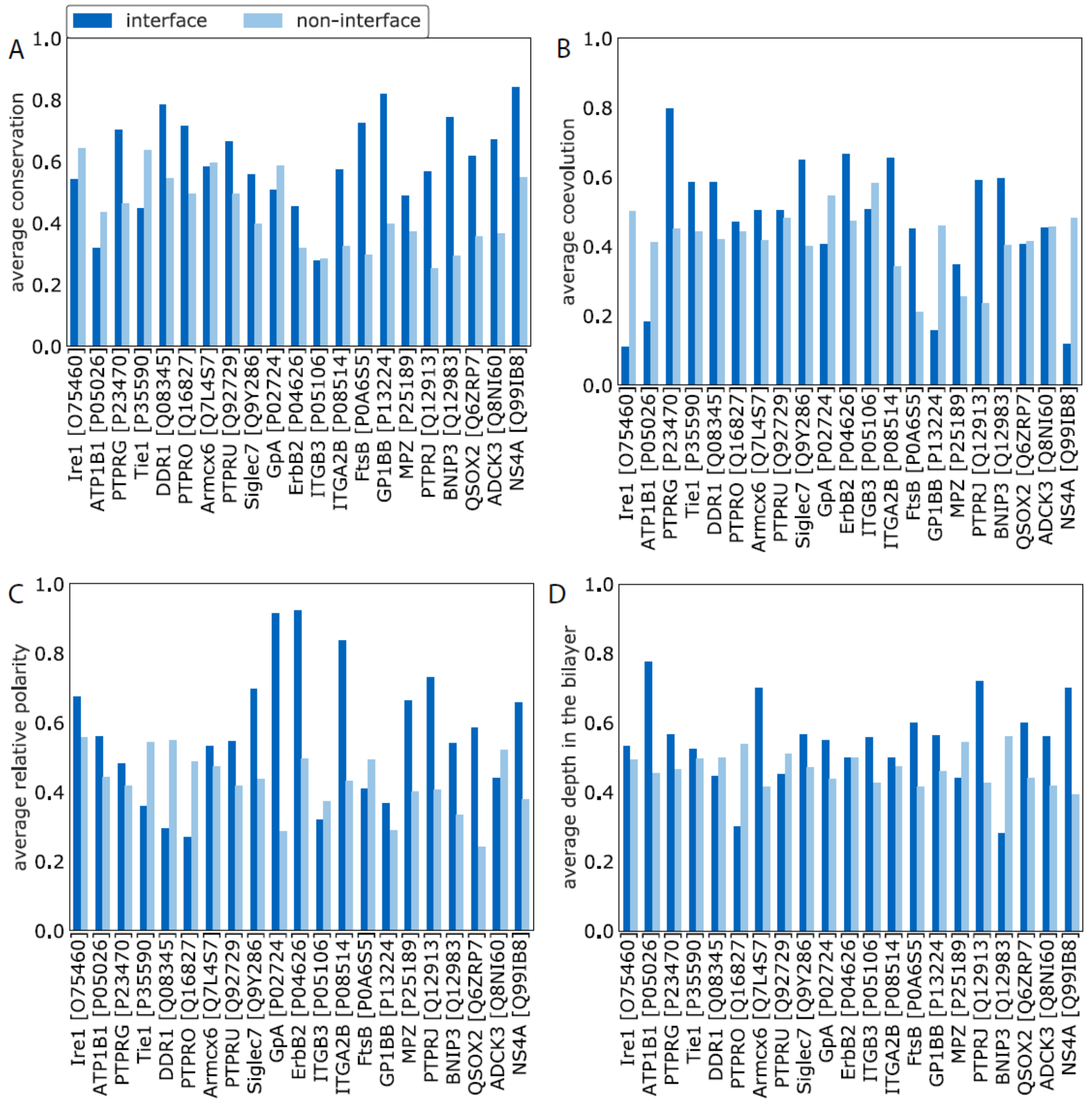

Fig S12. Average residue properties for interface and non-interface residues of the ETRA dataset.

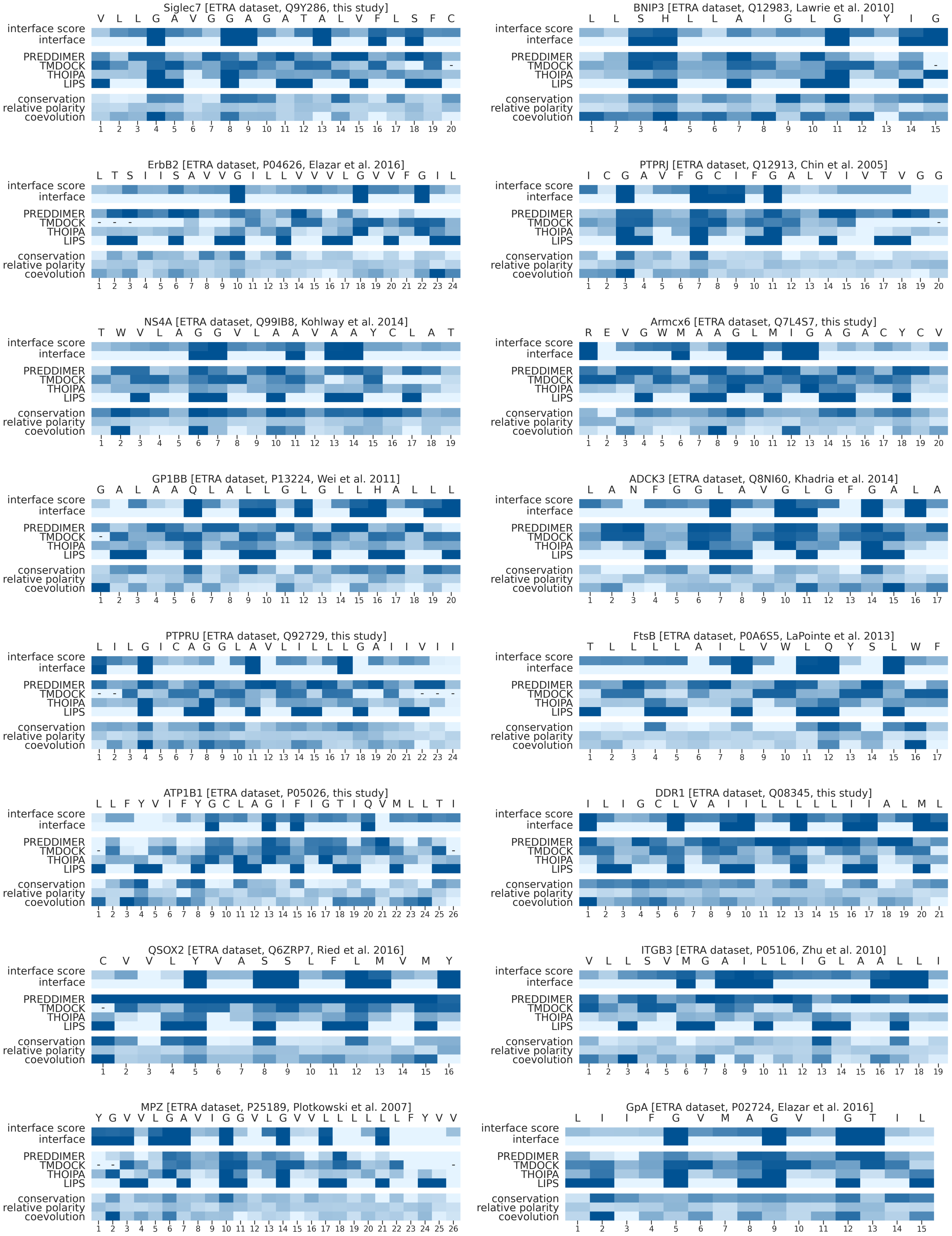
Siglec7 [ETRA dataset, Q9Y286, this study]
V L L G A V G G A G A T A L V F L S F C
BNIP3 [ETRA dataset, Q12983, Lawrie et al. 2010]
L L S H L L A I G L G I Y I G
ErbB2 [ETRA dataset, P04626, Elazar et al. 2016]
L T S I I S A V V G I L L V V V L G V V F G I L
PTPRJ [ETRA dataset, Q12913, Chin et al. 2005]
I C G A V F G C I F G A L V I V T V G G
NS4A [ETRA dataset, Q99IB8, Kohlway et al. 2014]
T W V L A G G V L A A V A A Y C L A T
Armcx6 [ETRA dataset, Q7L4S7, this study]
R E V G W M A A G L M I G A G A C Y C V
GP1BB [ETRA dataset, P13224, Wei et al. 2011]
G A L A A Q L A L L G L G L L H A L L L
ADCK3 [ETRA dataset, Q8NI60, Khadria et al. 2014]
L A N F G G L A V G L G F G A L A
PTPRU [ETRA dataset, Q92729, this study]
L I L G I C A G G L A V L I L L L G A I I V I I
FtsB [ETRA dataset, P0A6S5, LaPointe et al. 2013]
T L L L L A I L V W L Q Y S L W F
ATP1B1 [ETRA dataset, P05026, this study]
L L F Y V I F Y G C L A G I F I G T I Q V M L L T I
DDR1 [ETRA dataset, Q08345, this study]
I L I G C L V A I I L L L L L I I A L M L
QSOX2 [ETRA dataset, Q6ZRP7, Ried et al. 2016]
C V V L Y V A S S L F L M V M Y
ITGB3 [ETRA dataset, P05106, Zhu et al. 2010]
V L L S V M G A I L L I G L A A L L I
MPZ [ETRA dataset, P25189, Plotkowski et al. 2007]
Y G V V L G A V I G G V L G V V L L L L L L F Y V V
GpA [ETRA dataset, P02724, Elazar et al. 2016]
L I I F G V M A G V I G T I L
interface score
interface
PREDDIMER
TMDOCK
THOIPA
LIPS
conservation
relative polarity
coevolution

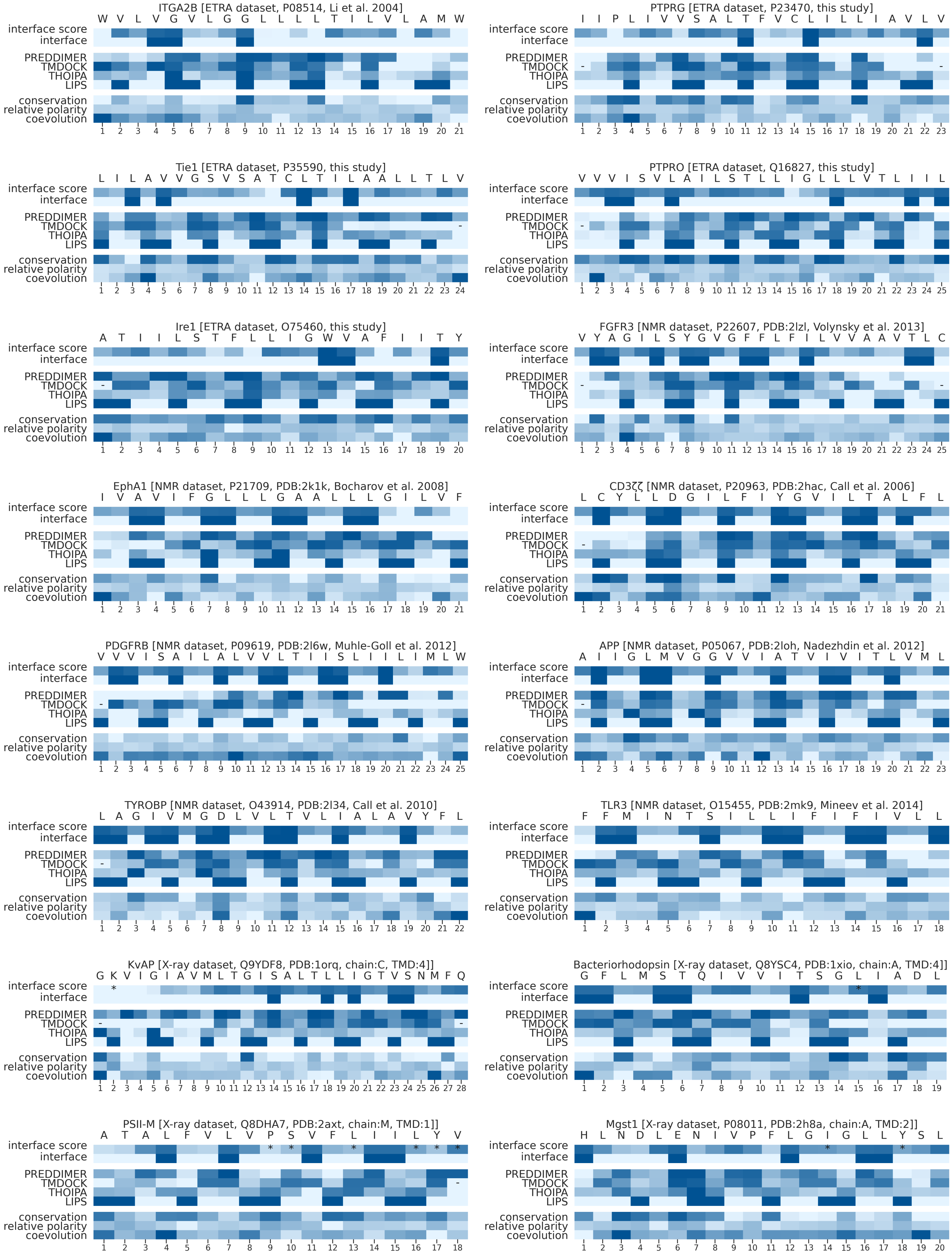
ITGA2B [ETRA dataset, P08514, Li et al. 2004]
W V L V G V L G G L L L L T I L V L A M W
PTPRG [ETRA dataset, P23470, this study]
I I P L I V V S A L T F V C L I L L I A V L V
Tie1 [ETRA dataset, P35590, this study]
L I L A V V G S V S A T C L T I L A A L L T L V
PTPRO [ETRA dataset, Q16827, this study]
V V V I S V L A I L S T L L I G L L L V T L I I L
Ire1 [ETRA dataset, O75460, this study]
A T I I L S T F L L I G W V A F I I T Y
FGFR3 [NMR dataset, P22607, PDB:2lzl, Volynsky et al. 2013]
V Y A G I L S Y G V G F F L F I L V V A A V T L C
EphA1 [NMR dataset, P21709, PDB:2k1k, Bocharov et al. 2008]
I V A V I F G L L L G A A L L L G I L V F
CD3ζζ [NMR dataset, P20963, PDB:2hac, Call et al. 2006]
L C Y L L D G I L F I Y G V I L T A L F L
PDGFRB [NMR dataset, P09619, PDB:2l6w, Muhle-Goll et al. 2012]
V V V I S A I L A L V V L T I I S L I I L I M L W
APP [NMR dataset, P05067, PDB:2loh, Nadezhdin et al. 2012]
A I I G L M V G G V V I A T V I V I T L V M L
TYROBP [NMR dataset, O43914, PDB:2l34, Call et al. 2010]
L A G I V M G D L V L T V L I A L A V Y F L
TLR3 [NMR dataset, O15455, PDB:2mk9, Mineev et al. 2014]
F F M I N T S I L L I F I F I V L L
KvAP [X-ray dataset, Q9YDF8, PDB:1orq, chain:C, TMD:4]]
G K V I G I A V M L T G I S A L T L L I G T V S N M F Q
Bacteriorhodopsin [X-ray dataset, Q8YSC4, PDB:1xio, chain:A, TMD:4]]
G F L M S T Q I V V I T S G L I A D L
PSII-M [X-ray dataset, Q8DHA7, PDB:2axt, chain:M, TMD:1]]
A T A L F V L V P S V F L I L Y V
Mgst1 [X-ray dataset, P08011, PDB:2h8a, chain:A, TMD:2]]
H L N D L E N I V P F L G I G L L Y S L
interface score
interface
PREDDIMER
TMDOCK
THOIPA
LIPS
conservation
relative polarity
coevolution

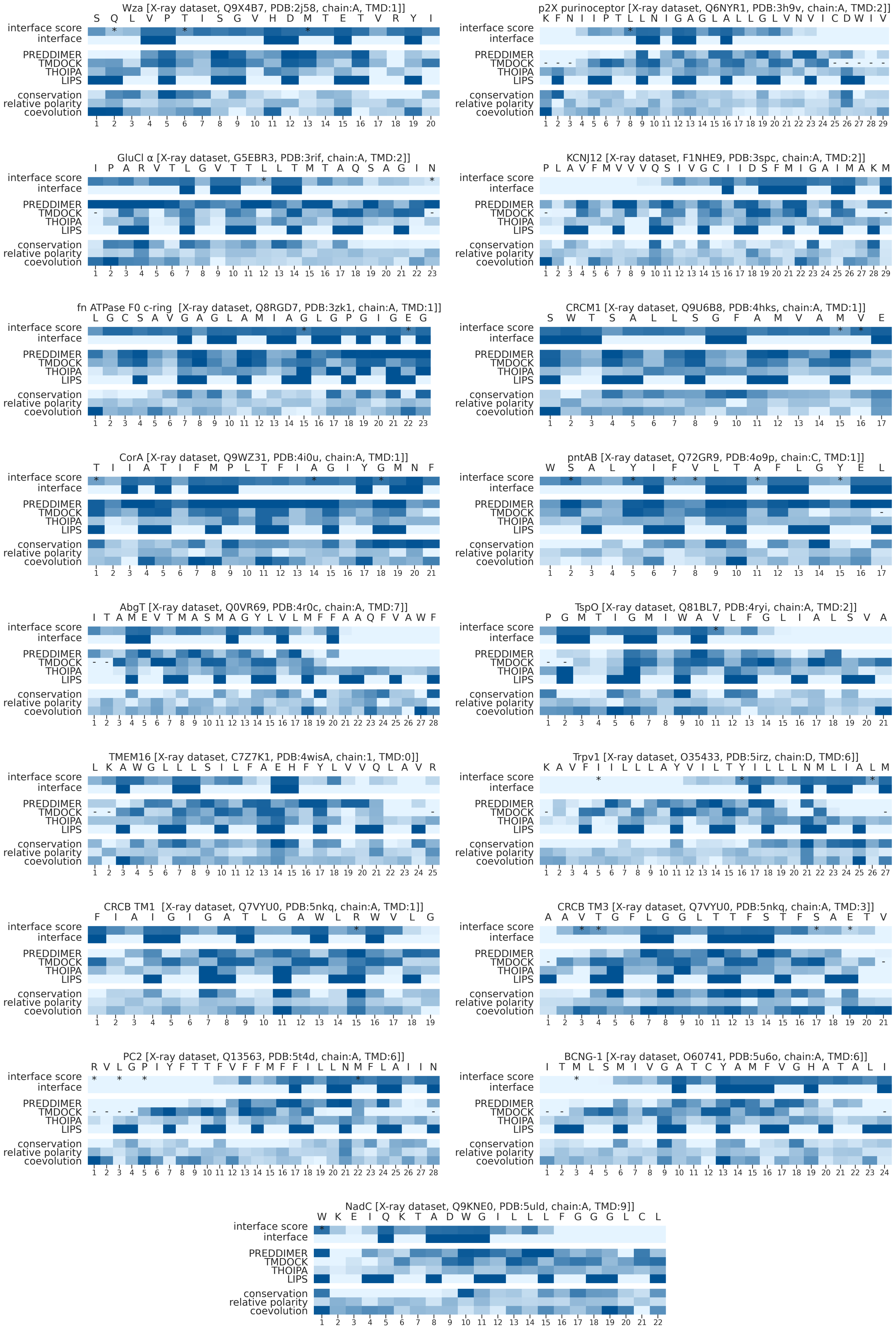
Wza [X-ray dataset, Q9X4B7, PDB:2j58, chain:A, TMD:1]]
p2X purinoceptor [X-ray dataset, Q6NYR1, PDB:3h9v, chain:A, TMD:2]]
GluCl α [X-ray dataset, G5EBR3, PDB:3rif, chain:A, TMD:2]]
KCNJ12 [X-ray dataset, F1NHE9, PDB:3spc, chain:A, TMD:2]]
fn ATPase F0 c-ring [X-ray dataset, Q8RGD7, PDB:3zk1, chain:A, TMD:1]]
CRCM1 [X-ray dataset, Q9U6B8, PDB:4hks, chain:A, TMD:1]]
CorA [X-ray dataset, Q9WZ31, PDB:4i0u, chain:A, TMD:1]]
pntAB [X-ray dataset, Q72GR9, PDB:4o9p, chain:C, TMD:1]]
AbgT [X-ray dataset, Q0VR69, PDB:4r0c, chain:A, TMD:7]]
TspO [X-ray dataset, Q81BL7, PDB:4ryi, chain:A, TMD:2]]
TMEM16 [X-ray dataset, C7Z7K1, PDB:4wisA, chain:1, TMD:0]]
Trpv1 [X-ray dataset, O35433, PDB:5irz, chain:D, TMD:6]]
CRCB TM1 [X-ray dataset, Q7VYU0, PDB:5nkq, chain:A, TMD:1]]
CRCB TM3 [X-ray dataset, Q7VYU0, PDB:5nkq, chain:A, TMD:3]]
PC2 [X-ray dataset, Q13563, PDB:5t4d, chain:A, TMD:6]]
BCNG-1 [X-ray dataset, O60741, PDB:5u6o, chain:A, TMD:6]]
NadC [X-ray dataset, Q9KNE0, PDB:5uld, chain:A, TMD:9]]
interface score
interface
PREDDIMER
TMDOCK
THOIPA
LIPS
conservation
relative polarity
coevolution

Fig S13. Heatmaps for each TMD, indicating residue properties, interface, and interface predictions. Darker shading indicates higher values for the interface score (see *Text S1. Supporting methods*), interface (boolean value based on >0.24 disruption or <3.5 Å heavy atom distance), PREDDIMER and TMDOCK scores (as calculated from closest heavy-atom distances in the top structures and normalised from 8 Å to 2.5 Å; values of 8 Å and above correspond to 0, and values 2.5 Å and below correspond to 1), THOIPA score (derived from leave-one-out validation and normalised from 0.15 to 0.5), LIPS score (boolean value describing participation in the helix face with the highest conservation and polarity), conservation (normalised from 1.5 to 3), relative polarity (normalised from 0.5 to 2.5) and coevolution (DImax, normalised from 0 to 1). A hyphen (-) indicates TMD positions truncated by TMDOCK, for which there was no structural prediction. For TMDs of the X-ray dataset, a star (*) in the interface score indicates residues that were involved in heterotypic TMD interactions (heterotypic contacts).

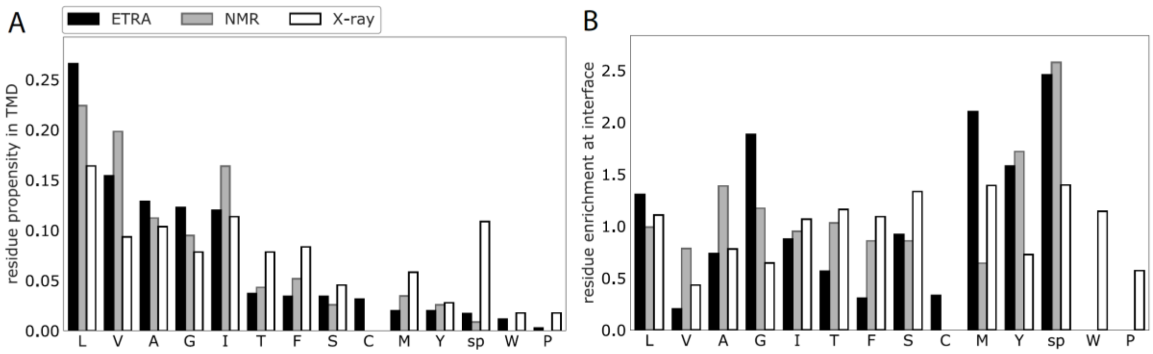

Fig S14. Amino acid propensities and enrichment at interfaces for all datasets. (A) Residue propensity within the full TMD region. (B) Enrichment of residues at interface positions, in comparison to non-interface positions. Residues are ordered according to the frequency of occurrence within the ETRA dataset. A value of 1.0 shows that the residue type is found at interfaces at the expected ratio, based on the total residue propensity. A value above 1.0 shows that the residue type is found more often at interfaces than would be expected based on the total propensity in the TMD (i.e. enriched at interfaces).

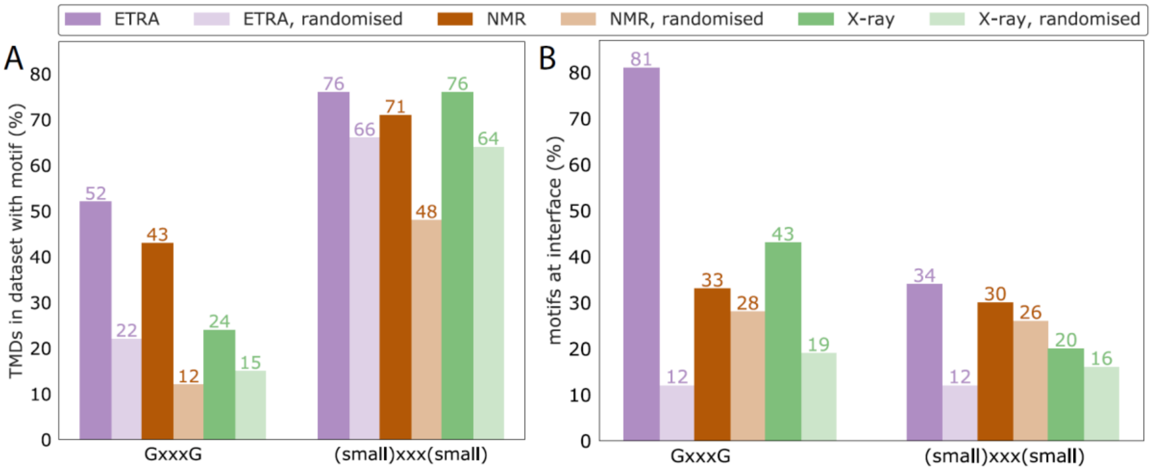

Fig S15. Motif occurrence and enrichment at interfaces for all datasets. (A) Motif abundance in the full TMD sequences. (B) Percentage of motifs where both residues are at the interface. As a comparison, the number of motifs is shown for randomised sequences with the same amino acid propensity. Note that GxxxG motifs are more strongly associated with interfaces than (small)xxx(small) motifs, especially for the ETRA dataset.

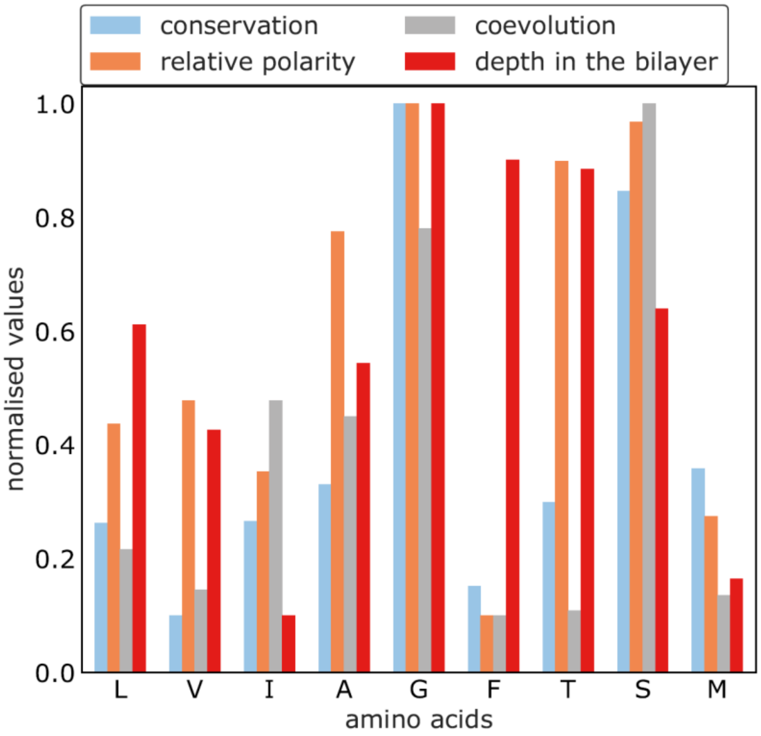

Fig S16. Positions with Gly residues show high conservation, coevolution, polarity, and depth in the bilayer. The data are shown for all residues of that type in the homotypic TMD dataset, regardless as to whether the residue is found at an oligomerisation interface. Only data for the residue types being present more than 40 times in the dataset are shown. Each property was normalised between 0.1 and 1 for comparison. The residue types are ordered according to their propensity in the TMDs (Fig S14).

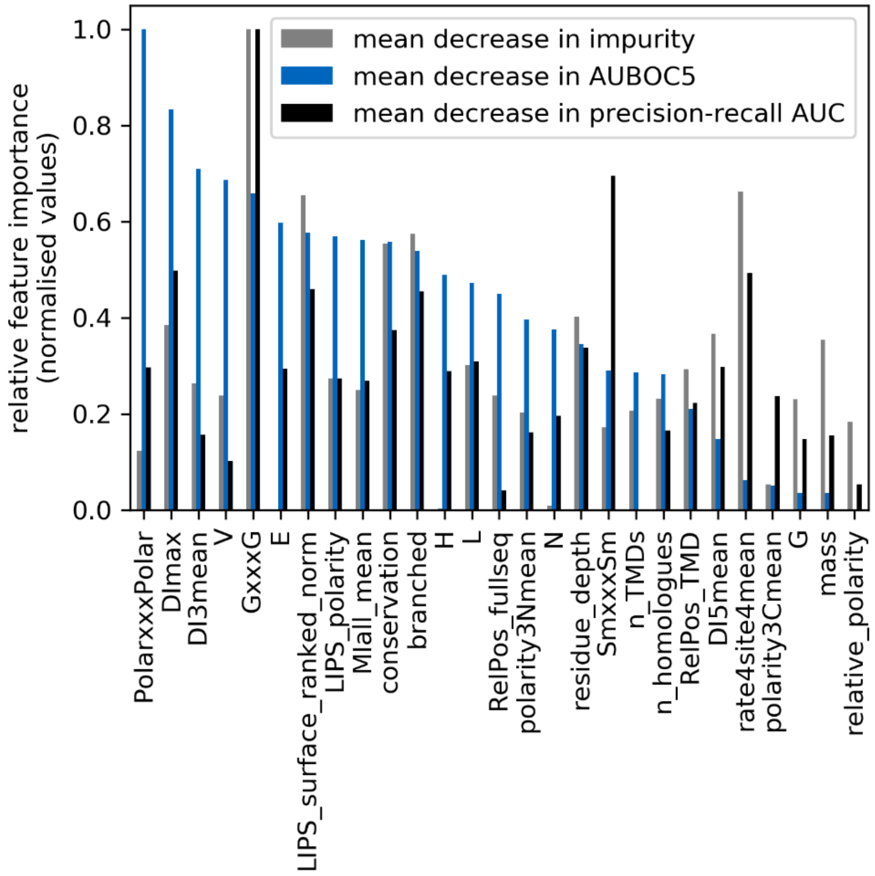

Fig S17. Importance of each feature in THOIPA prediction. The relative importance of each feature in the machine learning prediction was measured according to three different methods. The data were based on the 40 TMDs in the training dataset, after THOIPA feature reduction. The mean decrease in impurity was measured during training based on the entropy method. The "mean decrease in accuracy" was calculated for two different validation metrics, the AUBOC5 and the precision-recall area under the curve (precision-recall AUC). These were measured by randomly shuffling the data for the feature of interest amongst all residues, and repeating the training and cross-validation. Feature importance was taken as the decrease in validation score for in comparison to the original predictor. The values for each validation method were normalised between 0 and 1, and sorted by the mean decrease in AUBOC5.

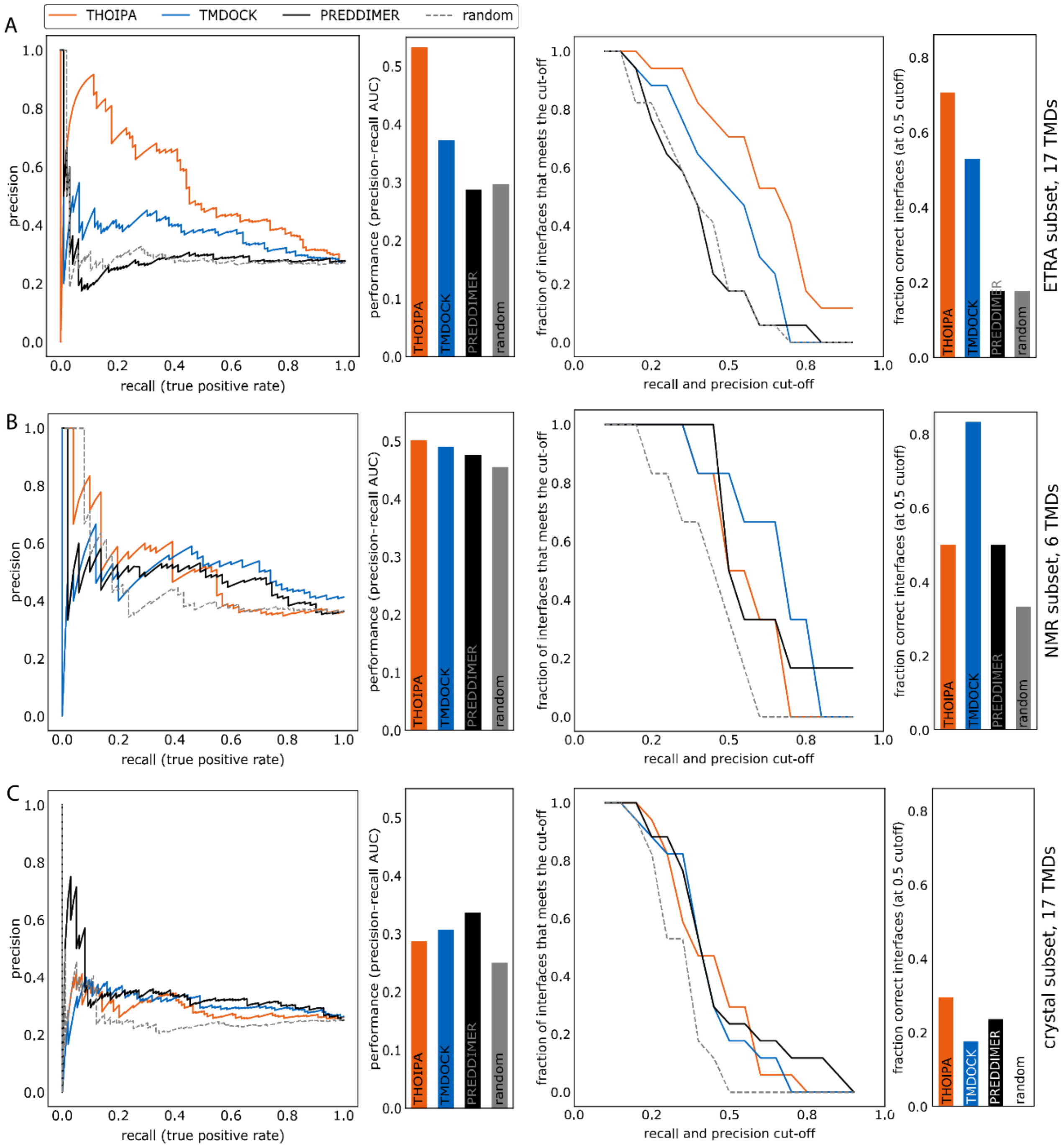

Fig S18. Validation of THOIPA performance towards the ETRA, NMR and X-ray datasets. The cross-validation performance is shown, segregated based on the experimental method used to identify the TMD interface. The underlying residue predictions are therefore identical to those used in **Fig 6A, C, and E**. For each dataset, the plots of precision-recall are shown on the left, and the of the plots of the fraction of correctly predicted interfaces [55] are shown on the right. (A) ETRA TMDs. (B) NMR TMDs. (C) X-ray TMDs.

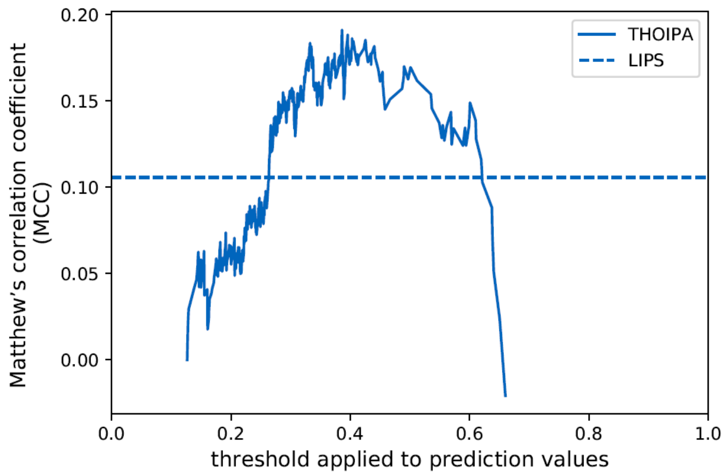

Fig S19. Comparison of THOIPA and LIPS performance. Cross-validation data is shown for the training dataset. LIPS gives binary prediction results (interface or non-interface) that could not be analysed with precision-recall curves. Instead, LIPS predictions were validated against the interface residues from experimental data using the Matthews correlation coefficient (MCC). Higher values indicate stronger prediction. LIPS gives a binary output, and therefore a single MCC. For THOIPA, the MCC depended on the chosen threshold. For most THOIPA thresholds, the THOIPA performance is superior to LIPS.

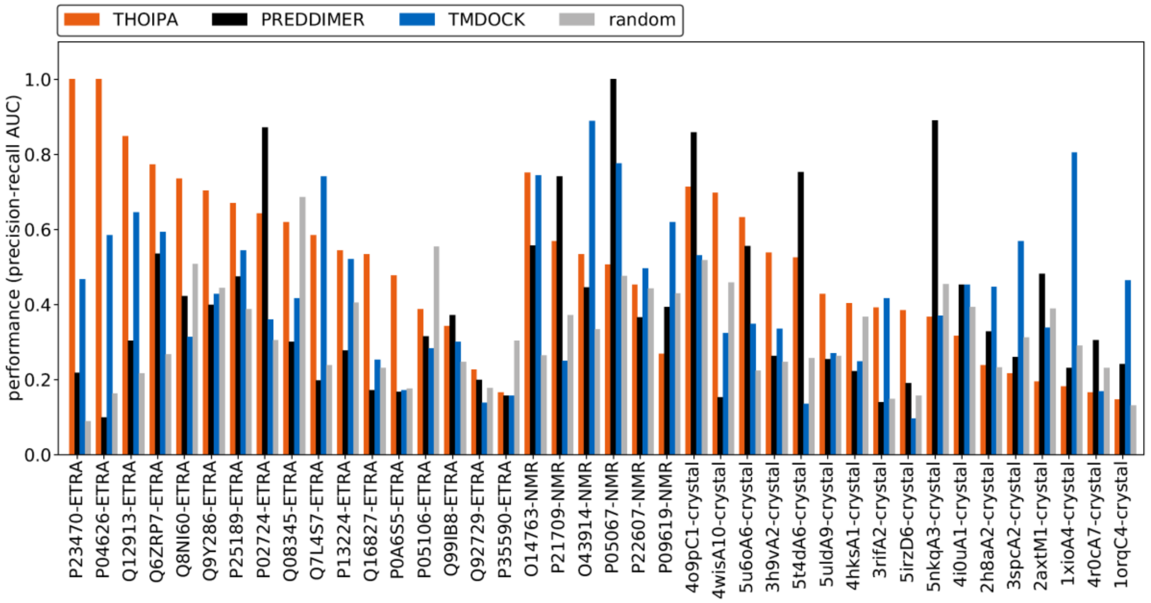

Fig S20. Performance of prediction algorithms validated for each TMD separately. The precision-recall area under the curve for the training dataset is shown. TMDs were ordered according to THOIPA performance within each experimental data type. For TMDs in the ETRA and NMR datasets, the respective UniProt accession is shown. Regarding nomenclature: for TMDs in the X-ray dataset the reference number (e.g. 2h8aA2) is a concatemer of the PDB accession (e.g. 2h8a), the chain (e.g. A), and the TMD number (e.g. 2).

Table S1. *T*-test data for all THOIPA features, comparing interface and non-interface residues

feature	higher for interface residues	t-test p-value (bootstrapped data)	correlated features ($R^2 > 0.6$, linear regression)
GxxxG	True	<0.00001	
cons*polarity	True	<0.00001	conservation
rate4site	False	<0.00001	LIPS_L*E, rate4site4mean
LIPS_L*E	False	<0.00001	rate4site
conservation	True	<0.00001	cons*polarity, cons4mean
rate4site4mean	False	<0.00001	rate4site
branched	False	<0.00001	
V	False	0.00004	
K	False	0.00008	KR
cons4mean	True	0.00022	conservation
LIPS_surface_ranked_norm	True	0.00070	LIPS_surface_ranked, LIPS_surface
relative_polarity	True	0.00076	polarity
PolarxxxPolar	True	0.00078	
G	True	0.00098	
LIPS_surface_ranked	True	0.00102	LIPS_surface_ranked_norm, LIPS_surface
SmxxxSm	True	0.00116	
LIPS_polarity	False	0.00132	LIV
DItop4mean	True	0.00142	DI8cum, DItop4mean_raw, DI4cum, DItop8mean, DImax_raw, DI4_inclusive_max, DImax
MI5mean	False	0.00148	DIall_mean, MIall_mean, MI5mean_raw, MItop8mean, MItop4mean
DItop4mean_raw	True	0.00220	DItop4mean, DItop8mean_raw, DImax_raw, DI4_inclusive_max_raw
LIPS_surface	True	0.00232	LIPS_surface_ranked_norm, LIPS_surface_ranked
residue_depth	True	0.00380	
DIall_mean_raw	False	0.00392	MI3mean, MI4_inclusive_mean, DIall_mean, MIall_mean, MI4mean, MI1mean, MI4_inclusive_max, MImax, MItop8mean, MItop4mean
MI4_inclusive_max	False	0.00400	MI3mean, MI4_inclusive_mean, MI2mean, MI4value, DIall_mean, MIall_mean, MI4mean, MI1mean, DIall_mean_raw, MImax, MItop8mean, MI4_inclusive_max_raw, MItop4mean
DI4cum	True	0.00408	DI8cum, DItop4mean, DImax_raw, DImax
MI4mean	False	0.00430	MI4_inclusive_mean, MI4value, DIall_mean, MIall_mean, MI4_inclusive_max, DIall_mean_raw, MI4value_raw, MI4mean_raw, MImax, MItop8mean, MItop4mean
MI3mean	False	0.00534	MI4_inclusive_mean, DIall_mean, MIall_mean, MI3mean_raw, MI4_inclusive_max, DIall_mean_raw, MImax, MItop8mean, MItop4mean
MImax	False	0.00600	MI3mean, MI4_inclusive_mean, MI2mean, MI4value, DIall_mean, MIall_mean, MItop4mean_raw, MI4mean, MI1mean, MI4_inclusive_max, DIall_mean_raw, MI4_inclusive_mean_raw, MImax_raw, MItop8mean, MItop8mean_raw, MI4_inclusive_max_raw, MItop4mean
MItop8mean	False	0.00622	MI3mean, MI4_inclusive_mean, MI2mean, MI4value, DIall_mean, MIall_mean,

			MItop4mean_raw, MI4mean, MI1mean, MI4_inclusive_max, DIall_mean_raw, MImax, MI4_inclusive_mean_raw, MItop8mean_raw, MI4_inclusive_max_raw, MI5mean, MItop4mean
MI4value	False	0.00634	MI4_inclusive_mean, DIall_mean, MIall_mean, MI4mean, MI4_inclusive_max, MI4value_raw, MI4mean_raw, MImax, MItop8mean, MItop4mean
MItop4mean	False	0.00756	MI3mean, MI4_inclusive_mean, MI2mean, MI4value, DIall_mean, MIall_mean, MItop4mean_raw, MI4mean, MI1mean, MI4_inclusive_max, DIall_mean_raw, MImax, MI4_inclusive_mean_raw, MImax_raw, MItop8mean, MItop8mean_raw, MI4_inclusive_max_raw, MI5mean
H	True	0.00834	
MIall_mean	False	0.00870	MI3mean, MI4_inclusive_mean, MI2mean, MI4value, DIall_mean, MI4mean, MI1mean, MI4_inclusive_max, DIall_mean_raw, MImax, MItop8mean, MI4_inclusive_max_raw, MI5mean, MItop4mean
MI4_inclusive_mean	False	0.00880	MI3mean, MI2mean, MI4value, DIall_mean, MIall_mean, MI4mean, MI1mean, MI4_inclusive_max, DIall_mean_raw, MImax, MI4_inclusive_mean_raw, MItop8mean, MI4_inclusive_max_raw, MItop4mean
DIall_mean	False	0.01366	MI3mean, MI4_inclusive_mean, MI2mean, MI4value, MIall_mean, MI4mean, MI1mean, MI4_inclusive_max, DIall_mean_raw, MImax, MItop8mean, MI5mean, MItop4mean
MI4mean_raw	False	0.01460	MI1mean_raw, MI2mean_raw, MI4value, MI3mean_raw, MItop4mean_raw, MI4mean, MI4value_raw, MI5mean_raw, MI4_inclusive_mean_raw, MImax_raw, MItop8mean_raw, MI4_inclusive_max_raw, MIall_mean_raw
MI2mean	False	0.01622	MI4_inclusive_mean, MI2mean_raw, DIall_mean, MIall_mean, MI4_inclusive_max, MImax, MItop8mean, MItop4mean
MI4_inclusive_max_raw	False	0.01624	MI4_inclusive_mean, MI1mean_raw, MI2mean_raw, MIall_mean, MI3mean_raw, MItop4mean_raw, MI4_inclusive_max, MI4value_raw, MI5mean_raw, MI4mean_raw, MImax, MI4_inclusive_mean_raw, MImax_raw, MItop8mean, MItop8mean_raw, MIall_mean_raw, MItop4mean
DImax_raw	True	0.01740	DI8cum, DItop4mean, DItop4mean_raw, DI4cum, DI4_inclusive_max_raw, DImax
MImax_raw	False	0.01862	MI1mean_raw, MI2mean_raw, MI3mean_raw, MItop4mean_raw, MI4value_raw, MI5mean_raw, MI4mean_raw, MImax, MI4_inclusive_mean_raw, MItop8mean_raw, MI4_inclusive_max_raw, MIall_mean_raw, MItop4mean
MItop8mean_raw	False	0.01950	MI1mean_raw, MI2mean_raw, MI3mean_raw, MItop4mean_raw, MI4value_raw, MI5mean_raw, MI4mean_raw, MImax, MI4_inclusive_mean_raw, MImax_raw, MItop8mean, MI4_inclusive_max_raw, MIall_mean_raw, MItop4mean
polarity	True	0.02058	relative_polarity, polarity1mean
MI4_inclusive_mean_raw	False	0.02130	MI4_inclusive_mean, MI1mean_raw, MI2mean_raw, MI3mean_raw, MItop4mean_raw, MI4value_raw, MI5mean_raw, MI4mean_raw, MImax, MImax_raw, MItop8mean, MItop8mean_raw, MI4_inclusive_max_raw, MIall_mean_raw, MItop4mean
MI4value_raw	False	0.02214	MI1mean_raw, MI2mean_raw, MI4value, MI3mean_raw, MItop4mean_raw, MI4mean, MI5mean_raw, MI4mean_raw, MI4_inclusive_mean_raw, MImax_raw, MItop8mean_raw, MI4_inclusive_max_raw, MIall_mean_raw
MI5mean_raw	False	0.02262	MI1mean_raw, MI2mean_raw, MI3mean_raw, MItop4mean_raw, MI4value_raw, MI4mean_raw,

			MI4_inclusive_mean_raw, MImax_raw, MItop8mean_raw, MI4_inclusive_max_raw, MI5mean, MIall_mean_raw
MItop4mean_raw	False	0.02416	MI1mean_raw, MI2mean_raw, MI3mean_raw, MI4value_raw, MI5mean_raw, MI4mean_raw, MImax, MI4_inclusive_mean_raw, MImax_raw, MItop8mean, MItop8mean_raw, MI4_inclusive_max_raw, MIall_mean_raw, MItop4mean
MI1mean	False	0.02434	MI4_inclusive_mean, MI1mean_raw, DIall_mean, MIall_mean, MI4_inclusive_max, DIall_mean_raw, MImax, MItop8mean, MItop4mean
DItop8mean_raw	True	0.02456	DItop4mean_raw, DItop8mean
MI3mean_raw	False	0.02662	MI3mean, MI1mean_raw, MI2mean_raw, MItop4mean_raw, MI4value_raw, MI5mean_raw, MI4mean_raw, MI4_inclusive_mean_raw, MImax_raw, MItop8mean_raw, MI4_inclusive_max_raw, MIall_mean_raw
E	True	0.02714	DE
DItop8mean	True	0.02850	DItop4mean, DItop8mean_raw
DImax	True	0.03160	DI8cum, DItop4mean, DI4cum, DImax_raw, DI4_inclusive_max
LIV	False	0.03438	LIPS_polarity, L
DI5mean	False	0.03508	DI5mean_raw
QN	True	0.03536	N, Q
MI2mean_raw	False	0.03836	MI1mean_raw, MI2mean, MI3mean_raw, MItop4mean_raw, MI4value_raw, MI5mean_raw, MI4mean_raw, MI4_inclusive_mean_raw, MImax_raw, MItop8mean_raw, MI4_inclusive_max_raw, MIall_mean_raw
DI5mean_raw	False	0.04266	DI5mean
MIall_mean_raw	False	0.04578	MI1mean_raw, MI2mean_raw, MI3mean_raw, MItop4mean_raw, MI4value_raw, MI5mean_raw, MI4mean_raw, MI4_inclusive_mean_raw, MImax_raw, MItop8mean_raw, MI4_inclusive_max_raw
polarity3Cmean	False	0.04618	
N	True	0.05058	QN
KR	False	0.05714	K, R
DI4_inclusive_max_raw	True	0.05722	DItop4mean_raw, DImax_raw, DI4_inclusive_max
polarity3Nmean	False	0.05742	
DE	True	0.10054	E, D
DI8cum	True	0.10276	DItop4mean, DI4cum, DImax_raw, DImax
polarity4mean	True	0.10436	
MI1mean_raw	False	0.11004	MI2mean_raw, MI3mean_raw, MItop4mean_raw, MI1mean, MI4value_raw, MI5mean_raw, MI4mean_raw, MI4_inclusive_mean_raw, MImax_raw, MItop8mean_raw, MI4_inclusive_max_raw, MIall_mean_raw
F	False	0.12724	
A	False	0.12960	
C	False	0.13260	CS
I	False	0.18050	
RelPos_fullseq	False	0.25548	
MI_highest_face	False	0.26594	DI_highest_face
n_TMDs	False	0.28932	
Q	True	0.30112	QN

CS	False	0.32702	S, C
DI4_inclusive_mean	False	0.32932	DI4_inclusive_mean_raw
DI3mean	False	0.34640	DI3mean_raw
DI1mean_raw	True	0.35354	DI1mean
DI4mean	True	0.47138	DI4value_raw, DI4value, DI4mean_raw
n_homologues	False	0.54044	
L	True	0.60208	LIV
W	True	0.65002	
DI2mean	True	0.65194	DI2mean_raw
D	True	0.67888	DE
DI4_inclusive_max	True	0.69020	DItop4mean, DI4_inclusive_max_raw, DImax
RelPos_TMD	True	0.71416	
DI4value	True	0.73712	DI4value_raw, DI4mean_raw, DI4mean
polarity1mean	True	0.75132	polarity
DI4_inclusive_mean_raw	True	0.75494	DI4_inclusive_mean
T	True	0.76118	
mass	False	0.76240	
DI_highest_face	False	0.78984	MI_highest_face
DI4value_raw	False	0.79416	DI4value, DI4mean_raw, DI4mean
DI4mean_raw	True	0.80826	DI4value_raw, DI4value, DI4mean
S	False	0.83534	CS
R	False	0.84656	KR
M	False	0.87000	
Y	True	0.88406	
P	True	0.89010	
DI3mean_raw	True	0.92916	DI3mean
DI1mean	False	0.93352	DI1mean_raw
DI2mean_raw	False	0.99648	DI2mean

References for supporting information

1. Langosch D, Brosig B, Kolmar H, Fritz HJ (1996) Dimerisation of the glycophorin A transmembrane segment in membranes probed with the ToxR transcription activator. J Mol Biol 263(4):525-30. http://dx.doi.org/10.1006/jmbi.1996.0595
2. Brosig B, Langosch D (1998) The dimerization motif of the glycophorin A transmembrane segment in membranes: Importance of glycine residues. Protein Sci 7(4):1052-6. http://dx.doi.org/10.1002/pro.5560070423
3. Schanzenbach C, Schmidt FC, Breckner P, Teese MG, Langosch D (2017) Identifying ionic interactions within a membrane using BLaTM, a genetic tool to measure homo-and heterotypic transmembrane helix-helix interactions. Scientific Reports 7(7):43476. http://dx.doi.org/10.1038/srep43476
4. Ried CL, Kube S, Kirrbach J, Langosch D (2012) Homotypic interaction and amino acid distribution of unilaterally conserved transmembrane helices. J Mol Biol 420(3):251-7. http://dx.doi.org/10.1016/j.jmb.2012.04.008
5. Li W, Godzik A (2006) CD-HIT: A fast program for clustering and comparing large sets of protein or nucleotide sequences. Bioinformatics 22(13):1658-9. http://dx.doi.org/10.1093/bioinformatics/btl158
6. Kohlway A, Pirakitikulr N, Barrera FN, Potapova O, Engelman DM, et al. (2014) Hepatitis C virus RNA replication and virus particle assembly require specific dimerization of the NS4A protein transmembrane domain. J Virol 88(1):628-42. http://dx.doi.org/10.1128/JVI.02052-13
7. Elazar A, Weinstein J, Biran I, Fridman Y, Bibi E, et al. (2016) Mutational scanning reveals the determinants of protein insertion and association energetics in the plasma membrane. eLife 5:e12125. http://dx.doi.org/10.7554/eLife.12125
8. Lawrie CM, Sulistijo ES, MacKenzie KR (2010) Intermonomer hydrogen bonds enhance GxxxG-driven dimerization of the BNIP3 transmembrane domain: Roles for sequence context in helix-helix association in membranes. J Mol Biol 396(4):924-36. http://dx.doi.org/10.1016/j.jmb.2009.12.023
9. Khadria AS, Mueller BK, Stefely JA, Tan CH, Pagliarini DJ, et al. (2014) A gly-zipper motif mediates homodimerization of the transmembrane domain of the mitochondrial kinase ADCK3. J Am Chem Soc 136(40):14068-77. http://dx.doi.org/10.1021/ja505017f
10. Ried CL, Scharnagl C, Langosch D (2016) Entrapment of water at the transmembrane helix-helix Interface of Quiescin Sulfhydryl Oxidase 2. Biochemistry 55(9):1287-90. http://dx.doi.org/10.1021/acs.biochem.5b01239
11. Khadria AS, Senes A (2013) The transmembrane domains of the bacterial cell division proteins FtsB and ftsL form a stable high-order oligomer. Biochemistry 52(43):7542-50.
12. Li R, Gorelik R, Nanda V, Law PB, Lear JD, et al. (2004) Dimerization of the transmembrane domain of integrin αIIb subunit in cell membranes. J Biol Chem 279(25):26666-73. http://dx.doi.org/10.1074/jbc.M314168200
13. Zhu H, Metcalf DG, Streu CN, Billings PC, DeGrado WF, et al. (2010) Specificity for homooligomer versus heterooligomer formation in integrin transmembrane helices. J Mol Biol 401(5):882-91. http://dx.doi.org/10.1016/j.jmb.2010.06.062
14. Wei P, Liu X, Hu MH, Zuo LM, Kai M, et al. (2011) The dimerization interface of the glycoprotein Ibβ transmembrane domain corresponds to polar residues within a leucine zipper motif. Protein Sci 20(11):1814-23. http://dx.doi.org/10.1002/pro.713
15. Plotkowski ML, Kim S, Phillips ML, Partridge AW, Deber CM, et al. (2007) Transmembrane domain of myelin protein zero can form dimers: Possible implications for myelin construction. Biochemistry 46(43):12164-73. http://dx.doi.org/10.1021/bi701066h

16. Chin CN, Sachs JN, Engelman DM (2005) Transmembrane homodimerization of receptor-like protein tyrosine phosphatases. FEBS Lett 579(17):3855-8. http://dx.doi.org/10.1016/j.febslet.2005.05.071
17. Beuming T, Weinstein H (2004) A knowledge-based scale for the analysis and prediction of buried and exposed faces of transmembrane domain proteins. Bioinformatics 20(12):1822-35. http://dx.doi.org/10.1093/bioinformatics/bth143
18. Adamian L, Liang J (2006) Prediction of buried helices in multispan alpha helical membrane proteins. Proteins Struct Funct Genet 63(1):1-5. http://dx.doi.org/10.1002/prot.20874
19. Walters RFS, DeGrado WF (2006) Helix-packing motifs in membrane proteins. Proc Natl Acad Sci USA 103(37):13658-63. http://dx.doi.org/10.1073/pnas.0605878103
20. Zhang SQ, Kulp DW, Schramm CA, Mravic M, Samish I, et al. (2015) The membrane- and soluble-protein helix-helix interactome: Similar geometry via different interactions. Structure 23(3):527-41. http://dx.doi.org/10.1016/j.str.2015.01.009
21. Kaján L, Hopf TA, Kalaš M, Marks DS, Rost B (2014) FreeContact: Fast and free software for protein contact prediction from residue co-evolution. BMC Bioinformatics 15(1):85. http://dx.doi.org/10.1186/1471-2105-15-85
22. Morcos F, Pagnani A, Lunt B, Bertolino A, Marks DS, et al. (2011) Direct-coupling analysis of residue coevolution captures native contacts across many protein families. Proc Natl Acad Sci USA 108(49):E1293-E301. http://dx.doi.org/10.1073/pnas.1111471108
23. Jones DT, Buchan DWA, Cozzetto D, Pontil M (2012) PSICOV: precise structural contact prediction using sparse inverse covariance estimation on large multiple sequence alignments. Bioinformatics 28(2):184-90. http://dx.doi.org/10.1093/bioinformatics/btr638
24. Hopf TA, Colwell LJ, Sheridan R, Rost B, Sander C, et al. (2012) Three-dimensional structures of membrane proteins from genomic sequencing. Cell 149(7):1607-21. http://dx.doi.org/10.1016/j.cell.2012.04.012
25. Marks DS, Colwell LJ, Sheridan R, Hopf TA, Pagnani A, et al. (2011) Protein 3D structure computed from evolutionary sequence variation. PLoS ONE 6(12):e28766. http://dx.doi.org/10.1371/journal.pone.0028766
26. Pupko T, Bell RE, Mayrose I, Glaser F, Ben-Tal N (2002) Rate4Site: An algorithmic tool for the identification of functional regions in proteins by surface mapping of evolutionary determinants within their homologues. Bioinformatics 18(SUPPL. 1):S71-S7. http://dx.doi.org/10.1093/bioinformatics/18.suppl_1.s71
27. Adamian L, Liang J (2006) Prediction of transmembrane helix orientation in polytopic membrane proteins. BMC Struct Biol 6:13. http://dx.doi.org/10.1186/1472-6807-6-13
28. Engelman DM, Steitz TA, Goldman A (1986) Identifying nonpolar transbilayer helices in amino acid sequences of membrane proteins. Annu Rev Biophys Biophys Chem 15:321-53. http://dx.doi.org/10.1146/annurev.bb.15.060186.001541
29. Kawashima S, Kanehisa M (2000) AAindex: Amino acid index database. Nucleic Acids Res 28(1):374.
30. Käll L, Krogh A, Sonnhammer ELL (2004) A combined transmembrane topology and signal peptide prediction method. J Mol Biol 338(5):1027-36. http://dx.doi.org/https://doi.org/10.1016/j.jmb.2004.03.016
31. Geurts P, Ernst D, Wehenkel L (2006) Extremely randomized trees. Machine Learning 63(1):3-42. http://dx.doi.org/10.1007/s10994-006-6226-1
32. Polyansky AA, Volynsky PE, Efremov RG (2012) Multistate organization of transmembrane helical protein dimers governed by the host membrane. J Am Chem Soc 134(35):14390-400. http://dx.doi.org/10.1021/ja303483k
33. Polyansky AA, Chugunov AO, Volynsky PE, Krylov NA, Nolde DE, et al. (2014) PREDDIMER: A web server for prediction of transmembrane helical dimers. Bioinformatics 30(6):889-90. http://dx.doi.org/10.1093/bioinformatics/btt645

34. Lomize AL, Pogozheva ID (2017) TMDOCK: An energy-based method for modeling α-helical dimers in membranes. J Mol Biol 429(3):390-8. http://dx.doi.org/https://doi.org/10.1016/j.jmb.2016.09.005
35. Finger C, Escher C, Schneider D (2009) The single transmembrane domains of human receptor tyrosine kinases encode self-interactions. Science Signaling 2:89. http://dx.doi.org/10.1126/scisignal.2000547
36. Noordeen NA, Carafoli F, Hohenester E, Horton MA, Leitinger B (2006) A transmembrane leucine zipper is required for activation of the dimeric receptor tyrosine kinase DDR1. J Biol Chem 281(32):22744-51. http://dx.doi.org/10.1074/jbc.M603233200
37. Barwe SP, Kim S, Rajasekaran SA, Bowie JU, Rajasekaran AK (2007) Janus model of the Na,K-ATPase β-subunit transmembrane domain: distinct faces mediate α/β assembly and β-β homo-oligomerization. J Mol Biol 365(3):706-14. http://dx.doi.org/10.1016/j.jmb.2006.10.029
38. Allegretti AS, Ortiz G, Kalim S, Wibecan J, Zhang D, et al. (2016) Siglec-7 as a Novel Biomarker to Predict Mortality in Decompensated Cirrhosis and Acute Kidney Injury. Dig Dis Sci 61(12):3609-20. http://dx.doi.org/10.1007/s10620-016-4316-x
39. Kirrbach J, Krugliak M, Ried CL, Pagel P, Arkin IT, et al. (2013) Self-interaction of transmembrane helices representing pre-clusters from the human single-span membrane proteins. Bioinformatics 29(13):1623-30. http://dx.doi.org/10.1093/bioinformatics/btt247
40. Siddiqui S, Schwarz F, Springer S, Khedri Z, Yu H, et al. (2017) Studies on the detection, expression, glycosylation, dimerization, and ligand binding properties of mouse Siglec-E. J Biol Chem 292(3):1029-37. http://dx.doi.org/10.1074/jbc.M116.738351
41. López-Doménech G, Serrat R, Mirra S, D'Aniello S, Somorjai I, et al. (2012) The Eutherian Armcx genes regulate mitochondrial trafficking in neurons and interact with Miro and Trak2. 3:814. http://dx.doi.org/10.1038/ncomms1829
42. Tirasophon W, Welihinda AA, Kaufman RJ (1998) A stress response pathway from the endoplasmic reticulum to the nucleus requires a novel bifunctional protein kinase/endoribonuclease (Ire1p) in mammalian cells. Genes Dev 12(12):1812-24. http://dx.doi.org/10.1101/gad.12.12.1812
43. Liu CY, Schröder M, Kaufman RJ (2000) Ligand-independent dimerization activates the stress response kinases IRE1 and PERK in the lumen of the endoplasmic reticulum. J Biol Chem 275(32):24881-5. http://dx.doi.org/10.1074/jbc.M004454200
44. Ali MMU, Bagratuni T, Davenport EL, Nowak PR, Silva-Santisteban MC, et al. (2011) Structure of the Ire1 autophosphorylation complex and implications for the unfolded protein response. EMBO J 30(5):894-905. http://dx.doi.org/10.1038/emboj.2011.18
45. Yoshida H, Matsui T, Yamamoto A, Okada T, Mori K (2001) XBP1 mRNA is induced by ATF6 and spliced by IRE1 in response to ER stress to produce a highly active transcription factor. Cell 107(7):881-91. http://dx.doi.org/10.1016/S0092-8674(01)00611-0
46. Casas-Tinto S, Zhang Y, Sanchez-Garcia J, Gomez-Velazquez M, Rincon-Limas DE, et al. (2011) The ER stress factor XBP1s prevents amyloid-β neurotoxicity. Hum Mol Genet 20(11):2144-60. http://dx.doi.org/10.1093/hmg/ddr100
47. Duran-Aniotz C, Cornejo VH, Espinoza S, Ardiles ÁO, Medinas DB, et al. (2017) IRE1 signaling exacerbates Alzheimer's disease pathogenesis. Acta Neuropathol 134(3):489-506. http://dx.doi.org/10.1007/s00401-017-1694-x
48. Cho H, Stanzione F, Oak A, Kim GH, Yerneni S, et al. (2019) Intrinsic structural features of the human IRE1α transmembrane domain sense membrane lipid saturation. Cell Reports 27(1):307-20.e5. http://dx.doi.org/10.1016/j.celrep.2019.03.017
49. Seegar TCM, Eller B, Tzvetkova-Robev D, Kolev MV, Henderson SC, et al. (2010) TIE1-TIE2 interactions mediate functional differences between angiopoietin ligands. Mol Cell 37(5):643-55. http://dx.doi.org/10.1016/j.molcel.2010.02.007
50. Tsiamis AC, Hayes P, Box H, Goodall AH, Bell PRF, et al. (2000) Characterization and regulation of the receptor tyrosine kinase Tie-1 in platelets. J Vasc Res 37(6):437-42. http://dx.doi.org/10.1159/000054075

51. Gerber D, Sal-Man N, Shai Y (2004) Two motifs within a transmembrane domain, one for homodimerization and the other for heterodimerization. J Biol Chem 279(20):21177-82. http://dx.doi.org/10.1074/jbc.M400847200
52. Teixeira PL, Mendenhall JL, Heinze S, Weiner B, Skwark MJ, et al. (2017) Membrane protein contact and structure prediction using co-evolution in conjunction with machine learning. PLOS ONE 12(5):e0177866. http://dx.doi.org/10.1371/journal.pone.0177866
53. Wang Y, Barth P (2015) Evolutionary-guided de novo structure prediction of self-associated transmembrane helical proteins with near-atomic accuracy. Nat Comms 6:7196. http://dx.doi.org/10.1038/ncomms8196
54. Caporaso JG, Smit S, Easton BC, Hunter L, Huttley GA, et al. (2008) Detecting coevolution without phylogenetic trees? Tree-ignorant metrics of coevolution perform as well as tree-aware metrics. BMC Evol Biol 8(1):327. http://dx.doi.org/10.1186/1471-2148-8-327
55. Lensink MF, Wodak SJ (2010) Blind predictions of protein interfaces by docking calculations in CAPRI. Proteins Struct Funct Bioinformat 78(15):3085-95. http://dx.doi.org/10.1002/prot.22850
